# Design, Synthesis and Biological Evaluation of *N*-phenylindole Derivatives as Pks13 Inhibitors against*Mycobacterium tuberculosis*

**DOI:** 10.3390/molecules27092844

**Published:** 2022-04-29

**Authors:** Yanpeng Cai, Wei Zhang, Shichun Lun, Tongtong Zhu, Weijun Xu, Fan Yang, Jie Tang, William R. Bishai, Lifang Yu

**Affiliations:** 1 Shanghai Engineering Research Center of Molecular Therapeutics and New Drug Development, School of Chemistry and Molecular Engineering, East China Normal University, 3663 North Zhongshan Road, Shanghai 200062, China; caiyanpengdl@163.com (Y.C.); zhangwei@chem.ecnu.edu.cn (W.Z.); zhutongtong816@163.com (T.Z.); xuweijundl@163.com (W.X.); fyang@chem.ecnu.edu.cn (F.Y.); 2 Center for Tuberculosis Research, Department of Medicine, Division of Infectious Disease, Johns Hopkins School of Medicine, Baltimore, MD 21231, USA; slun1@jhmi.edu; 3 Shanghai Key Laboratory of Green Chemistry and Chemical Process, School of Chemistry and Molecular Engineering, East China Normal University, 3663 North Zhongshan Road, Shanghai 200062, China; jtang@chem.ecnu.edu.cn

**Keywords:** tuberculosis, polyketide synthase 13, *N*-phenylindole derivatives, structure–activity relationship

## Abstract

Polyketide synthase 13 (Pks13), an essential enzyme for the survival of *Mycobacterium tuberculosis* (*Mtb*), is an attractive target for new anti-TB agents. In our previous work, we have identified 2-phenylindole derivatives against *Mtb*. The crystallography studies demonstrated that the two-position phenol was solvent-exposed in the Pks13-TE crystal structure and a crucial hydrogen bond was lost while introducing bulkier hydrophobic groups at indole *N* moieties. Thirty-six *N*-phenylindole derivatives were synthesized and evaluated for antitubercular activity using a structure-guided approach. The structure–activity relationship (SAR) studies resulted in the discovery of the potent Compounds **45** and **58** against *Mtb* H37Rv, with an MIC value of 0.0625 μg/mL and 0.125 μg/mL, respectively. The thermal stability analysis showed that they bind with high affinity to the Pks13-TE domain. Preliminary ADME evaluation showed that Compound **58** displayed modest human microsomal stability. This report further validates that targeting Pks13 is a valid strategy for the inhibition of *Mtb* and provides a novel scaffold for developing leading anti-TB compounds.

## 1. Introduction

**Figure 1:**
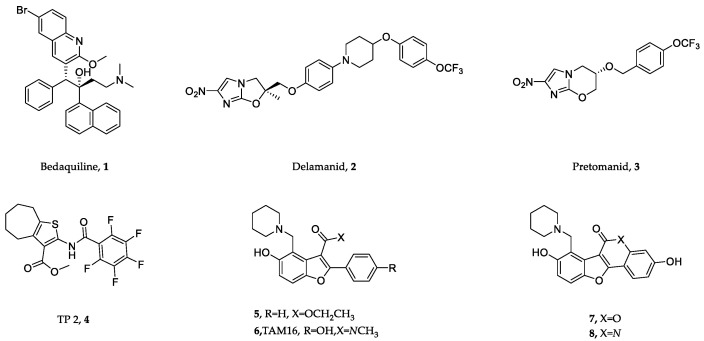
Anti-TB drugs: bedaquiline (**1**), delamanid (**2**), pretomanid (**3**), and selected Pks13 inhibitors (**4–8**).

Tuberculosis (TB) is a chronic infectious disease caused by *Mtb*, the second leading infectious killer after COVID-19. According to the 2021 World Health Organization (WHO) report, there were 1.5 million people who died from TB worldwide in 2020, including over 0.2 million co-infected with human immunodeficiency viruses (HIV) [1]. Multidrug-resistant (MDR) and extensively drug-resistant (XDR)-TB remain a public health crisis globally. In more than half a century, only three new anti-TB drugs, bedaquiline (**1**, Figure 1) [2], delamanid (**2**) [3], and pretomanid (**3**) [4], have entered the market. Recently, an inspiring outcome showed that a novel regimen of bedaquiline, pretomanid, and linezolid for treating highly drug-resistant pulmonary TB cured 90% of patients and cut the treatment period to 6 months [5]. Therefore, it is an urgent development of novel anti-TB drugs.

Blocking the biosynthesis pathway of the mycobacterial cell wall is an effective strategy for anti-TB. In *Mtb*, mycolic acids are C_60_-C_90_ long-chain fatty acids used to structure the unique cell wall, crucial for its persistence and pathogenesis [6]. Pks13 has been proved as the key enzyme that catalyzes the final step of mycolic acid synthesis [7]. Pks13, belonging to the type-I PKS family, comprises 1733 amino acid residues encoded by the *Pks13* gene [8]. Its topological structure is ACP (*N*-terminus acyl carrier protein), KS (ketoacyl synthase), AT (acyl transferase), ACP (C-terminus acyl carrier protein), and TE (thioesterase). The TE domain of Pks13 firstly exerts hydrolase activity, and ester bonds form after hydrolysis thioester between the mycolic β-ketoester and the hydroxyl group of Ser1533 [9]. Then, the domain acts as an acyltransferase to transfer mycolic β-ketoester onto trehalose to form the trehalose monomycolate precursor. Therefore, inhibiting Pks13 disturbs the biosynthesis of mycolic acids and kills *Mtb*. In recent years, as an emerging and attractive target, its inhibitors have been reported in succession: thiophene-based Pks13 inhibitors targeting the ACP domain (**4**) [10], benzofuran derivatives inhibitors Pks13-TE (**5**, **6**) [11], β-lactone-based compounds [12], and 4*H*-chromen-4-one derivatives [13], and our group reported conformational restricted tetracyclic compounds (**7**, **8**) [14,15,16].

**Figure 2:**
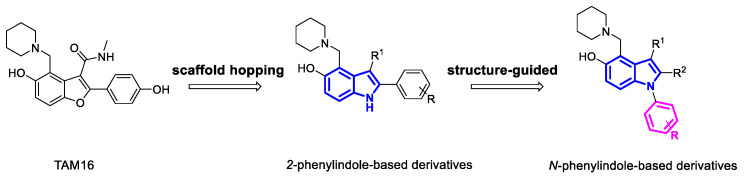
Design strategies of *N*-phenylindole derivatives.

**Figure 3:**
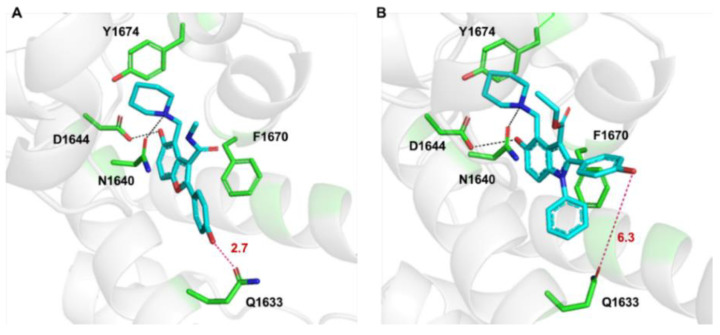
Crystal structures of Pks13-TE with TAM16 (PDB ID 5V3Y) (**A**) and proposed binding modes of Compound **18** (**B**). The key amino acid residues are colored green in the active site of Pks13-TE, and compounds are colored cyan.

Indole represents a privileged scaffold in various marine or terrestrial natural products with pharmacological and medical potential for developing novel and effective medications [17]. Indole derivatives have attracted considerable interest in medicinal scientists due to their broad, interesting bioactivities, including being anticancer [18], anti-inflammatory [19], antiviral [20], antimicrobial [21], and antitubercular [22,23,24], etc. In our previous work [14], adopting a scaffold hopping strategy, we have replaced the benzofuran core with the indole to identify novel anti-TB compounds. Unfortunately, 2-phenylindole-based derivatives (Figure 2) were deleterious for the activity compared with the corresponding benzofuran derivatives. The co-crystal structure between ligand TAM16 and the Pks13-thioesterase (TE) domain have been reported previously [11]. Similar to the binding mode of TAM16 reported previously (Figure 3A), the indole derivative **18** formed various hydrogen bonds interactions with key residues D1644 and N1640. The van der Waals and stacking interactions were observed between the piperidine ring and the side chain of the residue Y1674. However, a crucial hydrogen bond was lost between the hydroxyl group of the para position of 2-phenyl and Q1633 residue (Figure 3B). Herein, based on the structure-guided strategy, our continuous efforts developed *N*-phenylindole-based derivatives and evaluated their activity against *Mtb*.

## 2. Chemistry

**Scheme 1:**
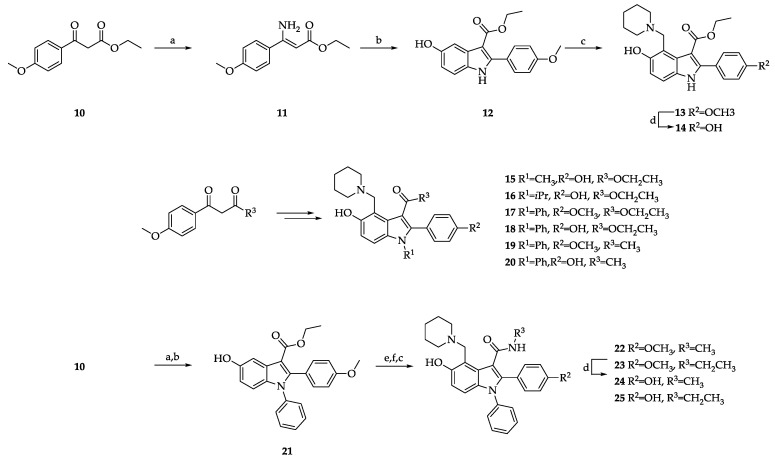
Synthesis of Compounds **13–20** and **22–25**. Reagents and conditions: (a) ammonium formate or aniline, CH_3_COOH, EtOH, reflux, 6–10 h; (b) benzoquinone, ZnBr_2_, rt, THF, 8–10 h; (c) formaldehyde (37% aq), piperidine, EtOH, reflux, 8–12 h; (d) BBr_3_ in CH_2_Cl_2_(1 M), CH_2_Cl_2_, rt, overnight; (e) NaOH(1N), EtOH, 90 °C, 2–6 h; (f) EDCI·HCl, HOBt, corresponding amines, DIPEA, DMF, rt, 5–8 h.

The compounds were synthesized in three to five steps by utilizing the synthetic routes shown in Scheme 1. Ethyl (*Z*)-3-amino-3-(4-methoxyphenyl) acrylate (**11**) was obtained from ethyl 3-(4-methoxyphenyl)-3-oxopropanoate (**10**) with ammonium formate in refluxing ethanol. The indole derivative (**12**) was formed from Compound **11** through a Nenitzescu reaction with 1,4-benzoquinone catalyzed by ZnBr_2_ and the subsequent Mannich reaction with 37% aqueous formaldehyde and piperidine to yield Compound **13** [25,26]. Compound **14** was obtained from **13** via demethylation by boron tribromide. Similarly, Compounds **15–20** were prepared in similar methods described above. The key intermediate (**21**) was synthesized following similar procedures as Compound **12**. The hydrolysis of Compound **21** was subsequently subjected to amide coupling and the Mannich reaction to afford Compounds **22** and **23**, and then demethylation by boron tribromide to give Compounds **24** and **25**.

**Scheme 2:**
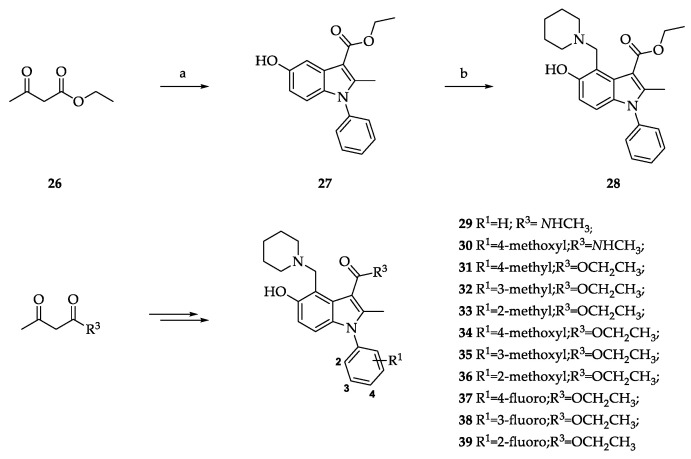
Synthesis of Compounds **28–39.** Reagents and conditions: (a) benzoquinone, aniline, montmorillonite, 1,2-dichloroethane, reflux, 7–12 h; (b) formaldehyde (37% aq), piperidine, EtOH, reflux, 8–12 h.

The synthesis route is shown in Scheme 2. Starting Material **26** with aniline and 1,4-benzoquinone three-component catalyzed by montmorillonite one-pot directly synthesized the 5-hydroxy indole derivative (**27**) [27] and the subsequent via formaldehyde and piperidine to give Compound **28**. Compounds **29–39** were prepared in a similar manner to Compound **28**.

**Scheme 3:**
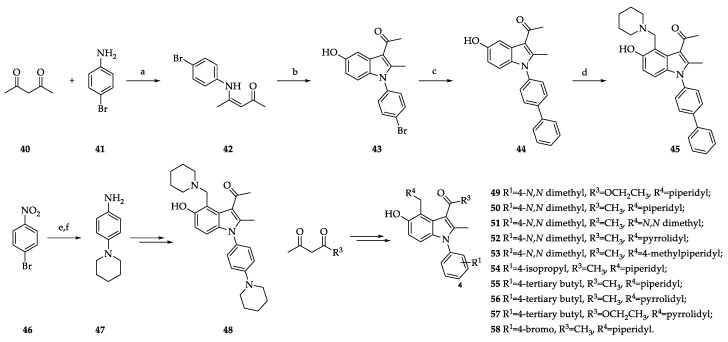
Synthesis of Compounds **45**, **48–58**. Reagents and conditions: (a) CH_3_COOH, EtOH, reflux, 6–10 h; (b) benzoquinone, ZnBr_2_, rt, THF, 8–10 h; (c) Pd(PPh_3_)_4_, Phenylboronic acid, K_2_CO_3_, toluene/EtOH/H_2_O = 3/2/1, 100 °C, 12 h; (d) formaldehyde (37% aq), piperidine, EtOH, reflux, 8–12 h; (e) K_2_CO_3_, piperidine, DMSO, 90 °C, 5 h; (f) Pd/C, H_2_, CH_3_OH, rt, 3 h.

The synthesis of Compounds **45** and **48–58** was shown in Scheme 3. Pentane-2,4-dione (**40**) and 4-bromoaniline (**41**) were refluxed in EtOH for 8 h to give Compound **42**. The indole derivative (**43**) was formed from Compound **42** through the Nenitzescu reaction, then, Suzuki coupling afforded **44**, which was subjected to the Mannich reaction to give the desired Compound **45**. Following a similar procedure, Compound **48** was formed from 4-(piperidin-1-yl) aniline (**47**), which was prepared via sequential nucleophilic substitution and reduction using piperidine and Pd/C, respectively, from 1-bromo-4-nitrobenzene (**46**). Synthesis of Compounds **49**–**58** was similar to Compound **45**, starting from pentane-2,4-dione (**40**) and ethyl 3-oxobutanoate (**26**).

## 3. Results and Discussion

All final compounds for anti-TB activity were initially evaluated for their minimal concentration of the 90% growth inhibitory against the *Mtb* H37Rv strain as their MIC values in a microplate alamar blue assay (MABA) [23]. Firstly, we explored substitutions at the nitrogen of indole with H, methyl, isopropyl, and phenyl, which resulted in Compounds **14–16** and **18**, with MIC values from 32 µg/mL to 2 µg/mL (Table 1). Surprisingly, a bulkier aromatic derivative (**18**) (MIC = 2 µg/mL) was favorable in activity against *Mtb*. Next, the ester group of 3-position on indole, which may be prone to metabolic liability, was assessed for the effects of replacement with acetyl and amides to give Compounds **20**, **24**, and **25**. Compared with Compound **18** (MIC = 2 µg/mL), the replacement with a methyl amide (**24**, MIC = 4 µg/mL) or ethyl amide (**25**, MIC = 8 µg/mL) at R^3^ resulted in a slight drop in activity against *Mtb*, whereas acetyl substituent (**20**) had an eight-fold decrease in activity. The antitubercular activity of methoxyl substitution on the two-position phenyl derivatives slightly decreased when compared to the corresponding hydroxyl substitution derivatives, e.g., **13** (MIC = 32 µg/mL) vs. **14** (MIC = 16 µg/mL), **17** (MIC = 4 µg/mL) vs. **18** (MIC = 2 µg/mL), **22** (MIC = 16 µg/mL) vs. **24** (MIC = 4 µg/mL), and **23** (MIC = 16 µg/mL) vs. **25** (MIC = 8 µg/mL), except **19** (MIC = 4 µg/mL) vs. **20** (MIC = 16 µg/mL).

Subsequently, we investigated 2-methyl-*N*-phenylindole derivatives. As shown in Table 2, the deletion of the two-position benzene ring resulted in Compound **28**, with an MIC value of 8 µg/mL. Among the series, the amide of three-position on indole resulted in Compounds **29** (MIC > 64 µg/mL) and **30** (MIC = 16 µg/mL), which were not favorable for activity. The *N*-phenyl of the indole was substituted with different groups, such as methoxyl, methyl, fluorine, *N*, *N*-dimethyl, and *t-*butyl to form Compounds **31–39**, **49**, and **57**, with MIC values ranging from 0.5 to 8 µg/mL. However, whether the substituent groups were electron-withdrawing or electron-donating, the para-position substituents of *N*-phenylindole were optimal. The activity of acetyl group substitution on the three-positions derivatives had a slight increase when compared to the corresponding ester group substitutions, e.g., **50** (MIC = 0.5 µg/mL) vs. **49** (MIC = 1 µg/mL) and **55** (MIC = 0.125 µg/mL) vs. **57** (MIC = 0.5 µg/mL). We kept investigating the effect of the para-position substituent at the *N*-phenyl of the indole. Gratifyingly, introducing hydrophobic groups (phenyl, piperidyl, *i*-propyl, *t-*butyl, and Br) resulted in compounds **45**, **48**, **54**, **55**, and **58** having considerable anti-TB activity against the *Mtb* strain, with MIC values of 0.125–0.0625 µg/mL. Compound **45** was the most potent, with an MIC value of 0.0625 µg/mL. Therefore, the loss of hydrogen bonds with Gln1633 seems to have little effect on the activity. Next, different Mannich substructures with *N*, *N*-dimethyl (**51**), pyrrolidinyl (**52** and **56**), and 4-methylpiperidyl (**53**) were placed at the four-position of the indole compounds. The substituent by the piperidine was the most potent, such as **55** (MIC = 0.125 µg/mL) vs. **56** (MIC = 0.25 µg/mL), **50** (MIC = 0.5 µg/mL) vs. **51**(MIC = 2 µg/mL), and **53** (MIC = 1 µg/mL), which were consistent with the SAR trend that we reported previously [11].

To demonstrate whether the indole compounds were binding to the Pks13-TE protein, selected representative compounds were evaluated for thermal stability in the presence of Pks13-TE by utilizing the nano-differential scanning fluorimetry (nano DSF) method [14]. The stabilization of the Pks13-TE protein at a 30 µM concentration upon binding of high-affinity ligands (at 150 µM and 300 µM concentration, respectively) was evaluated, while the *T*_m_ value of apo-Pks13-TE was 56.2 ± 0.03 °C. TAM16 and Compound **7** were used as a positive control. As shown in Table 3, it was observed that following the addition of the five-fold or 10-fold compounds (Δ*T*_m_ > 4.5 °C), there was a significant increase in the thermal stability of Pks13-TE, which indicated a high-affinity binding of the compounds to the Pks13-TE. Meanwhile, we observed that the extent of the Δ*T*_m_ values tends to be accompanied by the antitubercular activity of the corresponding compounds. These data implied that the antitubercular effect of *N*-phenylindole derivatives might be due to the targeting of the Pks13-TE.

Two compounds, **48** and **58**, were selected for the initial assessment of microsomal stability based on their potency. As shown in Table 4, the piperidinyl-substituted Compound **48** displayed a half-life of 20.7 min, while the bromine-substituted Compound **58** improved the metabolic stability profile, with a half-life of 58.0 min vs. 20.7 min for **48**.

## 4. Materials and Methods

### 4.1. General

All the solvents, starting materials, and chemical reagents were purchased from commercial sources and used without further purification, unless otherwise stated. Anhydrous tetrahydrofuran, dichloromethane, and 1,2-dichloroethane (DCE) were obtained by distillation over sodium wire or calcium hydride, respectively. TLC was performed on silica gel plates (GF254) to visualize components by UV light (254 nm). Column chromatography was carried out on silica gel (200–300 mesh). All non-aqueous reactions are carried out under a nitrogen atmosphere, the reagents do not contain water, and all reaction vessels are dried. ^1^H NMR spectra were obtained on Bruker at 400 MHz. ^13^C NMR spectra were obtained at 101 MHz (see Supplementary Materials). High-resolution mass spectra (HRMS) were performed using a Bruker ESI-TOF high-resolution mass spectrometer. NMR chemical shifts were reported in δ (ppm) using the δ 0 signal of tetramethylsilane, or the residual non-deuterated solvent signal (δ 7.26 signal for CDCl_3_, δ 3.31 signal for CD_3_OD, or δ 2.50 signal for (CD_3_)_2_SO, or in case of a mixed solvent, the CD_3_OD signal, as internal standards). The following multiplicity abbreviations are used: singlet (s), doublet (d), triplet (t), quartet (q), multiplet (m), and broads (br s). High-resolution mass spectra (HRMS) were performed using a Bruker ESI-TOF high-resolution mass spectrometer. The purity of all final compounds (≥95%) was established by high-performance liquid chromatography (HPLC), which was carried out on a Waters HPLC system using InertSustain-C18 column (5 µm, 250 × 4.6 mm), with a column temperature of 30 °C, detection wavelength at 254 and 280 nm, flow rate = 1 mL/min, and gradient of 5–95% CH_3_CN in water (containing 0.5 vol % of HCOOH) in 18 min.

*General procedure for the preparation of ethyl (Z)-3-amino-3-(4-methoxyphenyl) acrylate from ethyl 3-(4-methoxyphenyl)-3-oxopropanoate (method A)*

A commercially available mixture of **10** (1.0 mmol), ammonium formate (5.0 mmol), and molecular sieves (4 Å, 0.2 g) in 10 mL ethanol was refluxed for 8 h under N_2_ and then cooled to room temperature. The reaction mixture was filtered through celite. The filtrate was evaporated. The residue was extracted with ethyl acetate (3 × 20 mL), washed with NaCl aqueous solution, the combined organic layer was dried with anhydrous Na_2_SO_4,_ and the residues were purified by flash chromatography (petroleum ether: ethyl acetate = 20:1) to give **11**.

*General procedure for the preparation of indole construction (method B)*

To a solution of **11** (1.0 mmol) and ZnBr_2_ (1.0 mmol) in 3 mL, anhydrous THF has added a solution of benzoquinone (1.0 mmol) in THF (2 mL) under N_2_. After stirring for 6 h at room temperature. The reaction mixture was quenched with saturated NH_4_Cl aqueous solution and extracted with EtOAc (3 × 10mL). The combined organic phases were dried over anhydrous Na_2_SO_4_ and evaporated. The residue was purified by flash chromatography (petroleum ether: ethyl acetate = 10:1) to give **12**.

*General procedure for the Mannich reaction of 5-hydroxy indole derivatives with amine and formaldehyde (method C)*

To a solution of indole analogues (1 mmol) in ethanol (3 mL) were added formaldehyde (37% in water, 4 mmol) and the appropriate amine (4 mmol) at room temperature under N_2_. The reaction mixture was allowed to reflux for 8–12 h and then cooled to rt. The reaction mixture was evaporated, and the residue was purified by flash chromatography (dichloromethane: methanol = 60:1).

*General procedure for the One-pot reaction of 5-hydroxy indole derivatives (method D)*

Under the catalysis of montmorillonite (5 mmol), the corresponding amine (1 mmol) and ethyl acetoacetate (1 mmol) was dissolved in refluxing anhydrous DCE under N_2_ for 30 min and then to it was added benzoquinone (1 mmol) DCE solution dropwise. After reacting for 8 h, it was cooled to room temperature. The reaction mixture was filtered through celite; the filtrate was evaporated; the residue was purified by flash chromatography (petroleum ether: ethyl acetate = 5:1) to give the product.

*Ethyl (Z)-3-amino-3-(4-methoxyphenyl) acrylate* (**11**).

This compound was obtained from ethyl 3-(4-methoxyphenyl)-3-oxopropanoate **10** by employing Method A. Yield 92%; yellow oil.^1^H NMR (400 MHz, CDCl_3_) δ 7.49 (d, *J* = 8.0 Hz, 2H), 6.91 (d, *J* = 8.0 Hz, 2H), 4.93 (s, 1H), 4.20 (q, *J* = 7.0 Hz, 2H), 3.93 (s, 1H), 3.87 (s, 2H), 3.83 (s, 3H), and 1.25 (q, *J* = 7.0 Hz, 3H). ^13^C NMR (101 MHz, CDCl_3_) δ 191.1, 131.1, 127.6, 114.3, 114.1, 83.8, 61.6, 59.0, 55.7, 55.5, and 46.0.

*Ethyl 5-hydroxy-2-(4-methoxyphenyl)-1H-indole-3-*carboxylate (**12**).

This compound was obtained from Compound **11** by employing Method B. Yield 41%; pale yellow solid. ^1^H NMR (400 MHz, DMSO-*d*_6_) δ 11.70 (s, 1H), 8.93 (s, 1H), 7.60 (d, *J* = 8.2 Hz, 2H), 7.41 (s, 1H), 7.20 (d, *J* = 8.6 Hz, 1H), 7.03 (d, *J* = 8.2 Hz, 2H), 6.68 (d, *J* = 8.4 Hz, 1H), 4.17 (q, *J* = 7.0 Hz, 2H), 3.82 (s, 3H), and 1.24 (t, *J* = 7.0 Hz, 3H).

*Ethyl 5-hydroxy-2-(4-methoxyphenyl)-4-(piperidin-1-ylmethyl)-1H-indole-3-carboxylate* (**13**).

This compound was obtained from Compound **12** by employing Method C. Yield 82%; pale yellow solid. ^1^H NMR (400 MHz, CDCl_3_) δ 10.93 (s, 1H), 7.47–7.37 (m, 3H), 7.07 (d, *J* = 8.5 Hz, 1H), 6.85 (d, *J* = 7.8 Hz, 2H), 4.65 (s, 2H), 4.10 (q, *J* = 6.9 Hz, 2H), 3.79 (s, 3H), 3.54–3.22 (m, 2H), 3.04–2.72 (m, 2H), 1.89–1.77 (m, 4H), 1.53–1.24 (m, 2H), and 1.01 (t, *J* = 6.9 Hz, 3H). ^13^C NMR (101 MHz, CDCl_3_) δ 168.8, 160.1, 153.3, 146.2, 131.4, 131.0, 126.8, 125.1, 115.5, 115.3, 113.4, 106.9, 103.5, 60.8, 55.5, 53.9, 52.7, 23.9, 22.4, and 13.9. HRMS (ESI) *m/z*: Calcd for C_24_H_29_N_2_O_4_ (M + H)^+^, 409.2122, found 409.2141.

*Ethyl 5-hydroxy-2-(4-hydroxyphenyl)-4-(piperidin-1-ylmethyl)-1H-indole-3-carboxylate* (**14**).

To a solution of Compound **13** (1.0 mmol) in anhydrous CH_2_Cl_2_ (4 mL), BBr_3_ (1 M in CH_2_Cl_2_, 4.0 mmol) was added at room temperature under N_2_. After being stirred overnight, the reaction mixture was quenched with EtOH. The residue was purified by flash chromatography (dichloromethane: methanol = 50:1). Yield 89%; pale yellow solid. ^1^H NMR (400 MHz, CD_3_OD:CDCl_3_ = 1:2) δ 7.33 (d, *J* = 8.7 Hz, 1H), 7.28 (d, *J* = 7.8 Hz, 2H), 6.89–6.79 (m, 3H), 4.56 (s, 2H), 4.09 (q, *J* = 7.0 Hz, 2H), 3.57–3.33 (m, 2H), 3.21–2.86 (m, 2H), 2.06–1.47 (m, 6H), and 0.99 (t, *J* = 7.0 Hz, 3H). ^13^C NMR (101 MHz, CD_3_OD:CDCl_3_ = 1:2) δ 169.7, 158.6, 153.7, 147.9, 131.6, 131.4, 127.6, 124.6, 115.5, 115.3, 112.8, 105.9, 104.1, 61.3, 54.0, 53.2, 24.4, 22.6, and 14.0. HRMS (ESI) *m/z*: Calcd for C_23_H_27_N_2_O_4_ (M + H)^+^, 395.1965, found 395.1980.

*Ethyl 5-hydroxy-2-(4-hydroxyphenyl)-1-methyl-4-(piperidin-1-ylmethyl)-1H-indole-3-carboxylate* (**15**).

This compound was obtained from Compound **10**, methylamine, according to the methodology described for **14**. Overall yield 22%; yellow solid. ^1^H NMR (400 MHz, CD_3_OD:CDCl_3_ = 1:2) δ 7.38 (d, *J* = 8.8 Hz, 1H), 7.11 (d, *J* = 7.7 Hz, 2H), 6.99 (d, *J* = 8.8 Hz, 1H), 6.91 (d, *J* = 7.8 Hz, 2H), 4.62 (s, 2H), 3.98 (q, *J* = 7.0 Hz, 2H), 3.50 (s, 4H), 3.50–3.43 (m, 2H), 3.15–2.99 (m, 2H), 2.09–1.52 (m, 6H), and 0.83 (t, *J* = 7.0 Hz, 3H). ^13^C NMR (101 MHz, CD_3_OD:CDCl_3_ = 1:2) δ 169.2, 158.6, 154.2, 149.6, 132.6, 131.7, 126.8, 123.3, 115.5, 114.3, 113.0, 105.8, 105.5, 61.2, 53.6, 53.0, 31.5, 24.2, 22.4, and 13.7. HRMS (ESI) *m/z*: Calcd for C_24_H_29_N_2_O_4_ (M + H)^+^, 409.2122, found 409.2153.

*Ethyl 5-hydroxy-2-(4-hydroxyphenyl)-1-isopropyl-4-(piperidin-1-ylmethyl)-1H-indole-3-carboxylate* (**16**).

This compound was obtained from Compound **10**, isopropylamine, according to the methodology described for **14**. Overall yield 21%; pale yellow solid. ^1^H NMR (400 MHz, CD_3_OD:CDCl_3_ = 1:3) δ 7.55 (d, *J* = 9.0 Hz, 1H), 7.02 (d, *J* = 8.0 Hz, 2H), 6.95 (d, *J* = 9.0 Hz, 1H), 6.88 (d, *J* = 8.0 Hz, 2H), 4.50 (s, 2H), 4.40–4.37 (m, 1H), 3.88 (q, *J* = 7.0 Hz, 2H), 3.51–3.42 (m, 2H), 3.10–2.98 (m, 2H), 2.06–1.55 (m, 6H), 1.44 (d, *J* = 7.0 Hz, 6H), and 0.73 (t, *J* = 7.0 Hz, 3H). ^13^C NMR (101 MHz, CD_3_OD:CDCl_3_ = 1:3) δ 169.3, 158.3, 153.5, 148.9, 130.9, 129.7, 127.5, 123.6, 116.5, 115.5, 112.4, 105.4, 105.2, 61.1, 53.3, 52.8, 49.1, 24.1, 22.2, 21.4, and 13.5. HRMS (ESI) *m/z*: Calcd for C_26_H_33_N_2_O_4_ (M + H)^+^, 437.2435, found 437.2459.

*Ethyl 5-hydroxy-2-(4-methoxyphenyl)-1-phenyl-4-(piperidin-1-ylmethyl)-1H-indole-3-carboxylate* (**17**).

This compound was obtained from Compound **10**, aniline, by employing Methods A, B, and C. Overall yield 19%; pale yellow solid. ^1^H NMR (400 MHz, CDCl_3_) δ 7.35–7.28 (m, 3H), 7.14–7.11(m, 4H), 6.96 (d, *J* = 8.8 Hz, 1H), 6.79 (d, *J* = 8.8 Hz, 1H), 6.75 (d, *J* = 8.1 Hz, 2H), 4.17 (s, 2H), 4.14 (q, 7.0 Hz, 2H), 3.76 (s, 3H), 3.44–2.08 (m, 4H), 1.75–1.42 (m, 6H), and 1.05 (t, *J* = 7.0 Hz, 3H). ^13^C NMR (101 MHz, CDCl_3_) δ 167.2, 159.3, 154.6, 143.7, 137.2, 132.4, 131.9, 129.1, 128.4, 127.7, 124.6, 123.7, 114.2, 112.9, 110.9, 110.7, 107.5, 60.3, 58.6, 55.1, 53.8, 25.8, 23.9, and 13.9. HRMS (ESI) *m/z*: Calcd for C_30_H_33_N_2_O_4_ (M + H)^+^, 485.2435, found 485.2447.

*Ethyl 5-hydroxy-2-(4-hydroxyphenyl)-1-phenyl-4-(piperidin-1-ylmethyl)-1H-indole-3-carboxylate* (**18**).

This compound was obtained from Compound **17**, according to the methodology described for **13–14.** Yield 84%; pale yellow solid. ^1^H NMR (400 MHz, CD_3_OD:CDCl_3_ = 1:3) δ 7.40–7.29 (m, 3H), 7.11 (d, *J* = 6.6 Hz, 2H), 7.04 (d, *J* = 8.8 Hz, 1H), 6.99 (d, *J* = 8.0 Hz, 2H), 6.89 (d, *J* = 8.8 Hz, 1H), 6.67 (d, *J* = 8.0 Hz, 2H), 4.69 (s, 2H), 4.05 (q, *J* = 7.0 Hz, 2H), 3.67–3.48 (m, 2H), 3.25–3.01 (m, 2H), 2.10–1.53 (m, 6H), and 0.88 (t, *J* = 7.0 Hz, 3H). ^13^C NMR (101 MHz, CD_3_OD:CDCl_3_ = 1:3) δ 169.4, 158.3, 154.5, 149.2, 137.1, 133.8, 132.5, 130.0, 129.2, 129.1, 126.8, 123.3, 115.4, 115.1, 113.4, 106.9, 105.9, 61.5, 53.6, 53.3, 24.2, 22.6, and 13.8. HRMS (ESI) *m/z*: Calcd for C_29_H_31_N_2_O_4_ (M + H)^+^, 471.2278, found 471.2285.

*1-(5-hydroxy-2-(4-methoxyphenyl)-1-phenyl-4-(piperidin-1-ylmethyl)-1H-indol-3-yl) ethan-1-one* (**19**).

This compound was obtained from 1-(4-methoxyphenyl) butane-1,3-dione, aniline, by employing Methods A, B, and C. Overall yield 14%; pale yellow solid. ^1^H NMR (400 MHz, CDCl_3_) δ 7.85 (d, *J* = 7.6 Hz, 2H), 7.61–7.48 (m, 3H), 7.32 (d, *J* = 7.2 Hz, 2H), 7.08 (d, *J* = 8.4 Hz, 1H), 6.97 (d, *J* = 7.6 Hz, 2H), 6.93 (d, *J* = 8.8 Hz, 1H), 4.21 (s, 2H), 3.89 (s, 3H), 3.29–2.41 (m, 4H), 1.99 (s, 3H), and 1.86–1.39 (m, 6H). ^13^C NMR (101 MHz, CDCl_3_) δ 194.8, 163.8, 154.2, 136.6, 133.5, 133.4, 132.2, 130.0, 129.1, 128.3, 126.4, 115.8, 114.8, 114.2, 112.7, 109.1, 55.7, 53.3, 24.4, 22.9, and 14.1. HRMS (ESI) *m/z*: Calcd for C_29_H_31_N_2_O_3_ (M + H)^+^, 455.2329, found 455.2346.

*1-(5-hydroxy-2-(4-hydroxyphenyl)-1-phenyl-4-(piperidin-1-ylmethyl)-1H-indol-3-yl) ethan-1-one* (**20**).

This compound was obtained from Compound **19**, according to the methodology described for **13–14**. Yield 90%; brown solid. ^1^H NMR (400 MHz, CD_3_OD:CDCl_3_ = 1:1) δ 7.78 (d, *J* = 7.6 Hz, 2H), 7.61–7.52 (m, 3H), 7.32 (d, *J* = 7.4 Hz, 2H), 6.93 (d, *J* = 9.0 Hz, 1H), 6.89 (d, *J* = 7.8 Hz, 2H), 6.79 (d, *J* = 8.8 Hz, 1H), 4.20 (s, 2H), 3.28–2.36 (m, 4H), 1.95 (s, 3H), and 1.94–1.73 (m, 6H). ^13^C NMR (101 MHz, CD_3_OD:CDCl_3_ = 1:1) δ 196.5, 163.6, 154.1, 136.6, 133.8, 133.3, 131.8, 130.5, 129.8, 128.5, 126.8, 116.2, 115.1, 114.0, 113.0, 53.5, 25.0, 24.8, 22.8, and 14.6. HRMS (ESI) *m/z*: Calcd for C_28_H_29_N_2_O_3_ (M + H)^+^, 441.2173, found 441.2186.

*5-hydroxy-2-(4-methoxyphenyl)-N-methyl-1-phenyl-4-(piperidin-1-ylmethyl)-1H-indole-3-carboxamide* (**22**).

This compound was obtained from Compound **10**, aniline, employing Methods A–B to give Compound **21**. To a solution of Compound **21** (1.0 mmol) in ethanol (5 mL) was added NaOH aqueous solution (1N 10.0 mL) at rt. After stirring at refluxing for 4–6 h, the reaction mixture was acidified with 2 M aqueous HCl to pH 5–6. the residue was purified by flash chromatography (dichloromethane: methanol = 60:1) to give the product. Then, the result (1 mmol) in anhydrous DMF (5 mL) was added EDC·HCl (1.3 mmol), HOBt (1.3 mmol), *N, N*-diisopropylethylamine (2.1 mmol), and the methylamine hydrochloride (1.2 mmol) under N_2_ at rt. After stirring overnight at rt, the reaction mixture was quenched with saturated NH_4_Cl aqueous solution and extracted with EtOAc (2 × 30 mL). The combined organic phases were washed with NaCl aqueous solution, dried over anhydrous Na_2_SO_4_, and evaporated. The residue was purified by flash chromatography (dichloromethane: methanol = 40:1) to give the amide product. Next, we used Method C to give Compound **22**. Overall yield 12%; white solid. ^1^H NMR (400 MHz, CD_3_OD:CDCl_3_ = 1:2) δ 7.38–7.28 (m, 3H), 7.11–6.99 (m, 5H), 6.84 (d, *J* = 9.0 Hz, 1H), 6.75 (d, *J* = 8.2 Hz, 2H), 4.40 (s, 2H), 3.72 (s, 3H), 3.48–3.35 (m, 2H), 3.08–2.90 (m, 2H), 2.66 (s, 3H), and 2.07–1.60 (m, 6H). ^13^C NMR (101 MHz, CD_3_OD:CDCl_3_ = 1:2) δ 170.2, 160.5, 153.5, 142.7, 137.0, 133.3, 132.1, 129.9, 128.9, 128.8, 125.4, 122.2, 114.9, 114.4, 113.3, 110.4, 104.6, 55.5, 52.8, 27.0, 24.3, 22.8, and 22.5. HRMS (ESI) *m/z*: Calcd for C_29_H_32_N_3_O_3_ (M + H)^+^, 470.2438, found 470.2467.

*N-ethyl-5-hydroxy-2-(4-methoxyphenyl)-1-phenyl-4-(piperidin-1-ylmethyl)-1H-indole-3-carboxamide* (**23**).

This compound was obtained from Compound **22**, ethylamine, according to the methodology described for **13**. Overall yield 17%; pale yellow solid. ^1^H NMR (400 MHz, CD_3_OD:CDCl_3_ = 1:2) δ 7.39–7.27 (m, 3H), 7.16–7.08 (m, 4H), 6.95 (d, *J* = 8.8 Hz, 1H), 6.76 (d, *J* = 8.6 Hz, 2H), 6.68 (d, *J* = 8.8 Hz, 1H), 4.08 (s, 2H), 3.74 (s, 3H), 3.24 (q, *J* = 7.2 Hz, 2H), 2.92–2.41 (m, 4H), 1.76–1.50 (m, 6H), and 0.97 (t, *J* = 7.2 Hz, 3H). ^13^C NMR (101 MHz, CD_3_OD:CDCl_3_ = 1:2) δ 170.1, 160.4, 153.8, 140.2, 138.3, 133.2, 132.1, 129.9, 129.0, 128.4, 125.4, 123.6, 114.2, 114.0, 112.3, 111.5, 110.4, 57.7, 55.5, 54.2, 35.4, 26.2, 24.3, and 14.4. HRMS (ESI) *m/z*: Calcd for C_30_H_34_N_3_O_3_ (M + H)^+^, 484.2595, found 484.2623.

*5-hydroxy-2-(4-hydroxyphenyl)-N-methyl-1-phenyl-4-(piperidin-1-ylmethyl)-1H-indole-3-carboxamide* (**24**).

This compound was obtained from Compound **22**, according to the methodology described for **14**. Yield 88%; white solid. ^1^H NMR (400 MHz, CD_3_OD:CDCl_3_ = 1:2) δ 7.38–7.27 (m, 3H), 7.08 (d, *J* = 6.6 Hz, 2H), 7.03 (d, *J* = 8.8 Hz, 1H), 6.95 (d, *J* = 8.6 Hz, 2H), 6.81 (d, *J* = 8.8 Hz, 1H), 6.66 (d, *J* = 8.6 Hz, 2H), 4.41 (s, 2H), 3.53–3.30 (m, 2H), 3.10–2.88 (m, 2H), 2.67 (s, 3H), and 2.04–1.50 (m, 6H). ^13^C NMR (101 MHz, CD_3_OD:CDCl_3_ = 1:2) δ 170.5, 158.6, 153.6, 143.3, 137.4, 133.5, 132.4, 123.0, 129.0, 128.9, 125.7, 121.3, 115.9, 114.9, 113.2, 110.5, 105.0, 54.1, 53.0, 27.0, 24.5, and 22.7. HRMS (ESI) *m/z*: Calcd for C_28_H_30_N_3_O_3_ (M + H)^+^, 456.2282, found 456.2292.

*N-ethyl-5-hydroxy-2-(4-hydroxyphenyl)-1-phenyl-4-(piperidin-1-ylmethyl)-1H-indole-3-carboxamide* (**25**).

This compound was obtained from Compound **23**, according to the methodology described for **14**. Yield 83%; pale yellow solid. ^1^H NMR (400 MHz, CD_3_OD:CDCl_3_ = 1:3) δ 7.41–7.32 (m, 3H), 7.12 (d, *J* = 6.5 Hz, 2H), 7.07 (d, *J* = 8.9 Hz, 1H), 7.01 (d, *J* = 7.9 Hz, 2H), 6.86 (d, *J* = 8.9 Hz, 1H), 6.76–6.68 (m, 2H), 4.44 (s, 2H), 3.53–3.34 (m, 2H), 3.21 (q, *J* = 7.1 Hz, 2H), 3.07–2.96 (m, 2H), 2.15–1.71 (m, 6H), and 0.87 (t, *J* = 7.2 Hz, 3H). ^13^C NMR (101 MHz, CD_3_OD:CDCl_3_ = 1:3) δ 169.3, 158.6, 153.5, 143.3, 137.0, 133.3, 132.3, 131.3 129.8, 128.8, 125.5, 121.0, 115.9, 114.8, 113.2, 110.4, 104.8, 52.9, 52.8, 35.3, 24.4, 22.5, and 14.1. HRMS (ESI) *m/z*: Calcd for C_29_H_32_N_3_O_3_ (M + H)^+^, 470.2438, found 470.2458.

*Ethyl 5-hydroxy-2-methyl-1-phenyl-1H-indole-3-carboxylate* (**27**).

This compound was obtained from ethyl acetoacetate (**26**), aniline, employing Method D. Yield 47%; dark brown solid. ^1^H NMR (400 MHz, DMSO-*d*_6_) δ 9.29 (s, 1H), 7.67–7.54 (m, 3H), 7.48–7.41 (m, 2H), 7.19 (s, 1H), 6.90 (d, *J* = 7.8 Hz, 1H), 6.65 (d, *J* = 7.8 Hz, 1H), 4.31 (q, *J* = 6.8 Hz, 2H), 2.48 (s, 3H), and 1.38 (t, *J* = 6.8 Hz, 3H). ^13^C NMR (101 MHz, DMSO-*d6*) δ 165.1, 153.1, 145.4 144.8, 143.0, 136.1, 129.9, 128.9, 127.9, 115.6, 110.7, 105.5, 103.6, 58.9, 14.6, and 12.9.

*Ethyl 5-hydroxy-2-methyl-1-phenyl-4-(piperidin-1-ylmethyl)-1H-indole-3-carboxylate* (**28**).

This compound was obtained from Compound **27**, employing Method C. Yield 88%; brown solid. ^1^H NMR (400 MHz, CDCl_3_) δ 7.62–7.51 (m, 3H), 7.29–7.24 (m, 2H), 7.18 (d, *J* = 8.8 Hz, 1H), 6.87 (d, *J* = 8.8 Hz, 1H), 4.98 (s, 2H), 4.41 (q, *J* = 7.1 Hz, 2H), 3.60–3.31 (m, 2H), 3.16–2.86 (m, 2H), 2.46 (s, 3H), 2.16–1.54 (m, 6H), and 1.43 (t, *J* = 7.1 Hz, 3H). ^13^C NMR (101 MHz, CDCl_3_) δ 167.7, 154.0, 145.9, 136.2, 133.7, 130.0, 129.4, 128.5, 126.4, 116.5, 113.8, 108.9, 105.5, 60.8, 53.9, 52.7, 23.6, 22.5, 14.7, and 14.7. HRMS (ESI) *m/z*: Calcd for C_24_H_29_N_2_O_3_ (M + H)^+^, 393.2173, found 393.2190.

*5-hydroxy-N,2-dimethyl-1-phenyl-4-(piperidin-1-ylmethyl)-1H-indole-3-carboxamide* (**29**).

This compound was obtained from *N*-methyl-3-oxobutanamide and aniline, employing Methods D and C. Overall yield 41%; pale brown solid. ^1^H NMR (400 MHz, CD_3_OD:CDCl_3_ = 1:1) δ 7.60–7.51 (m, 3H), 7.29 (d, *J* = 7.4 Hz, 2H), 6.91 (d, *J* = 8.8 Hz, 1H), 6.76 (d, *J* = 8.8 Hz, 1H), 4.38 (s, 2H), 3.49–3.35 (m, 2H), 3.12–3.04 (m, 2H), 2.99 (s, 3H), 2.33 (s, 3H), and 2.03–1.72 (m, 6H). ^13^C NMR (101 MHz, CD_3_OD:CDCl_3_ = 1:1) δ 170.5, 153.2, 140.1, 136.8, 133.3, 130.4, 129.6, 128.6, 125.6, 114.3, 112.3, 110.2, 104.7, 52.9, 52.8, 27.0, 24.3, 23.0, and 13.0. HRMS (ESI) *m/z*: Calcd for C_23_H_28_N_3_O_2_ (M + H)^+^, 378.2176, found 378.2199.

*5-hydroxy-1-(4-methoxyphenyl)-N,2-dimethyl-4-(piperidin-1-ylmethyl)-1H-indole-3-carboxamide* (**30**).

This compound was obtained from *N*-methyl-3-oxobutanamide and 4-methoxyaniline, employing Methods D and C. Overall yield 35%; pale brown solid. ^1^H NMR (400 MHz, CD_3_OD:CDCl_3_ = 1:1) δ 7.15 (d, *J* = 8.2 Hz, 2H), 6.99 (d, *J* = 8.2 Hz, 2H), 6.82 (d, *J* = 8.8 Hz, 1H), 6.75 (d, *J* = 8.8 Hz, 1H), 4.27 (s, 2H), 3.82 (s, 3H), 3.42–3.30 (m, 2H), 2.94 (s, 3H), 2.93–2.83 (m, 2H), 2.29 (s, 3H), and 1.98–1.48 (m, 6H). ^13^C NMR (101 MHz, CD_3_OD:CDCl_3_ = 1:1) δ 170.3, 160.1, 152.8, 140.3, 133.2, 129.5, 129.1, 125.2, 115.2, 114.0, 112.1, 109.5, 104.2, 55.8, 52.6, 52.4, 26.8, 24.0, 22.3, and 13.0. HRMS (ESI) *m/z*: Calcd for C_24_H_30_N_3_O_3_ (M + H)^+^, 408.2282, found 408.2311.

*Ethyl 5-hydroxy-2-methyl-4-(piperidin-1-ylmethyl)-1-(p-tolyl)-1H-indole-3-carboxylate (***31**).

This compound was obtained from ethyl acetoacetate (**26**) and 4-methylaniline, employing Methods D and C. Overall yield 41%; pale yellow solid. ^1^H NMR (400 MHz, CDCl_3_) δ 7.34 (d, *J* = 7.6 Hz, 2H), 7.16 (d, *J* = 8.8 Hz, 1H), 7.11 (d, *J* = 7.6 Hz, 2H), 6.84 (d, *J* = 8.8 Hz, 1H), 4.93 (s, 2H), 4.38 (q, *J* = 7.0 Hz, 2H), 3.55–3.29 (m, 2H), 3.10–2.86 (m, 2H), 2.45 (s, 3H), 2.43 (s, 3H), 2.04–1.88 (br, 6H), and 1.41 (t, *J* = 7.0 Hz, 3H). ^13^C NMR (101 MHz, CDCl_3_) δ 167.8, 154.0, 146.0, 139.5, 133.7, 133.5, 130.6, 128.1, 126.3, 116.2, 113.8, 108.6, 105.2, 60.7, 53.9, 52.6, 23.7, 22.5, 21.4, and 14.6. HRMS (ESI) *m/z*: Calcd for C_25_H_31_N_2_O_3_ (M + H)^+^, 407.2329, found 407.2303.

*Ethyl 5-hydroxy-2-methyl-4-(piperidin-1-ylmethyl)-1-(m-tolyl)-1H-indole-3-carboxylate* (**32**).

This compound was obtained from ethyl acetoacetate (**26**) and 3-methylaniline, employing Methods D and C. Overall yield 35%; pale yellow solid. ^1^H NMR (400 MHz, CDCl_3_) δ 7.45 (t, *J* = 7.6 Hz, 1H), 7.34 (d, *J* = 7.4 Hz, 1H), 7.15 (d, *J* = 8.8 Hz, 1H), 7.09–7.03 (m, 2H), 6.88 (d, *J* = 8.8 Hz, 1H), 5.00 (s, 2H), 4.39 (q, *J* = 7.0 Hz, 2H), 3.52–3.33 (m, 2H), 3.11–2.92 (m, 2H), 2.46 (s, 3H), 2.44 (s, 3H), 2.09–1.80 (m, 6H), and 1.43 (t, *J* = 7.1 Hz, 3H). ^13^C NMR (101 MHz, CDCl_3_) δ 167.7, 153.9, 146.0, 140.3, 136.2, 133.8, 130.2, 129.8, 129.0, 126.4, 125.4, 116.9, 114.0, 109.3, 105.3, 60.7, 52.7, 31.6, 23.6, 22.5, 21.5 14.8, and 14.7. HRMS (ESI) *m/z*: Calcd for C_25_H_31_N_2_O_3_ (M + H)^+^, 407.2329, found 407.2351.

*Ethyl 5-hydroxy-2-methyl-4-(piperidin-1-ylmethyl)-1-(o-tolyl)-1H-indole-3-carboxylate* (**33**).

This compound was obtained from ethyl acetoacetate (**26**) and 2-methylaniline, employing Methods D and C. Overall yield 33%; yellow solid. ^1^H NMR (400 MHz, CDCl_3_) δ 7.48–7.39 (m, 2H), 7.36 (t, *J* = 7.4 Hz, 1H), 7.16–7.09 (m, 2H), 6.66 (d, *J* = 8.8 Hz, 1H), 4.96–4.92 (m, 2H), 4.39 (q, *J* = 7.0 Hz, 2H), 3.53–3.26 (m, 2H), 3.11–2.81 (m, 2H), 2.36 (s, 3H), 2.07–1.65 (m, 9H), and 1.42 (t, *J* = 7.0 Hz, 3H). ^13^C NMR (101 MHz, CDCl_3_) δ 167.6, 154.0, 145.9, 137.0, 135.1, 132.9, 131.6, 129.9, 129.0, 127.6, 126.3, 116.5, 113.4, 109.4, 105.1, 60.7, 54.4, 52.8, 29.8, 23.8, 22.6, 17.3, 14.7, and 14.2. HRMS (ESI) *m/z*: Calcd for C_25_H_31_N_2_O_3_ (M + H)^+^, 407.2329, found 407.2355.

*Ethyl 5-hydroxy-1-(4-methoxyphenyl)-2-methyl-4-(piperidin-1-ylmethyl)-1H-indole-3-carboxylate* (**34**).

This compound was obtained from ethyl acetoacetate (**26**) and 4-methoxyaniline, employing Methods D and C. Overall yield 41%; yellow solid. ^1^H NMR (400 MHz, CD_3_OD:CDCl_3_ = 1:2) δ 7.17 (d, *J* = 8.5 Hz, 2H), 7.08 (d, *J* = 8.5 Hz, 2H), 6.88 (d, *J* = 8.8 Hz, 1H), 6.82 (d, *J* = 8.8 Hz, 1H), 4.67 (s, 2H), 4.41 (q, *J* = 7.1 Hz, 2H), 3.89 (s, 3H), 3.63–3.45 (m, 2H), 3.19–3.00 (m, 2H), 2.46 (s, 3H), 2.10–1.53 (m, 6H), and 1.42 (t, *J* = 7.1 Hz, 3H). ^13^C NMR (101 MHz, CD_3_OD:CDCl_3_ = 1:2) δ 169.2, 160.8, 154.3, 147.5, 134.0, 129.8, 128.8, 126.8, 115.7, 114.9, 112.5, 105.5, 105.3, 61.6, 55.9, 53.6, 53.0, 24.2, 22.4, 14.8, and 14.6. HRMS (ESI) *m/z*: Calcd for C_25_H_31_N_2_O_4_ (M + H)^+^, 423.2278, found 423.2300.

*Ethyl 5-hydroxy-1-(3-methoxyphenyl)-2-methyl-4-(piperidin-1-ylmethyl)-1H-indole-3-carboxylate* (**35**).

This compound was obtained from ethyl acetoacetate (**26**) and 3-methoxyaniline, employing Methods D and C. Overall yield 47%; yellow oil. ^1^H NMR (400 MHz, CDCl_3_) δ 7.46 (t, *J* = 7.8 Hz, 1H), 7.13 (d, *J* = 8.8 Hz, 1H), 7.06 (d, *J* = 8.6 Hz, 1H), 6.90 (d, *J* = 8.8 Hz, 1H), 6.84 (d, *J* = 7.7 Hz, 1H), 6.77 (s, 1H), 4.92 (s, 2H), 4.39 (q, *J* = 7.0 Hz, 2H), 3.83 (s, 3H), 3.56–3.22 (m, 2H), 3.13–2.76 (m, 2H), 2.46 (s, 3H), 2.06–1.53 (m, 6H), and 1.42 (t, *J* = 7.0 Hz, 3H). ^13^C NMR (101 MHz, CDCl_3_) δ 167.6, 160.8, 154.0, 145.7, 137.3, 133.6, 130.7, 126.3, 120.6, 116.5, 115.1, 114.1, 113.7, 109.3, 105.5, 60.7, 55.7, 54.3, 52.8, 23.8, 22.6, 14.7, and 14.6. HRMS (ESI) *m/z*: Calcd for C_25_H_31_N_2_O_4_ (M + H)^+^, 423.2278, found 423.2308.

*Ethyl 5-hydroxy-1-(2-methoxyphenyl)-2-methyl-4-(piperidin-1-ylmethyl)-1H-indole-3-carboxylate* (**36**).

This compound was obtained from ethyl acetoacetate (**26**) and 2-methoxyaniline, employing Methods D and C. Overall yield 43%; yellow oil. ^1^H NMR (400 MHz, CDCl_3_) δ 7.47 (t, *J* = 7.8 Hz, 1H), 7.22 (d, *J* = 7.7 Hz, 1H), 7.13–7.05 (m, 2H), 6.67 (q, *J* = 8.7 Hz, 2H), 4.39 (q, *J* = 7.0 Hz, 2H), 4.32–4.17 (m, 2H), 3.72 (s, 3H), 3.10–1.82 (m, 7H), 1.65 (br, 6H), and 1.42 (t, *J* = 7.0 Hz, 3H). ^13^C NMR (101 MHz, CDCl_3_) δ 167.0, 156.0, 154.8, 144.7, 132.5, 130.5, 130.3, 125.3, 125.0, 121.1, 113.2, 112.4, 111.4, 110.1, 105.6, 60.1, 59.4, 55.8, 53.9, 26.1, 24.2, 14.8, and 13.2. HRMS (ESI) *m/z*: Calcd for C_25_H_31_N_2_O_4_ (M + H)^+^, 423.2278, found 423.2305.

*Ethyl 1-(4-fluorophenyl)-5-hydroxy-2-methyl-4-(piperidin-1-ylmethyl)-1H-indole-3-carboxylate* (**37**).

This compound was obtained from ethyl acetoacetate (**26**) and 4-fluoroaniline, employing Methods D and C. Overall yield 47%; pale brown solid. ^1^H NMR (400 MHz, CDCl_3_) δ 7.28–7.25 (m, 4H), 7.08 (d, *J* = 8.6 Hz, 1H), 6.83 (d, *J* = 8.8 Hz, 1H), 4.84 (s, 2H), 4.40 (q, *J* = 7.0 Hz, 2H), 3.49–3.16 (m, 2H), 3.02–2.59 (m, 2H), 2.44 (s, 3H), 1.96–1.57 (m, 6H), and 1.43 (t, *J* = 7.0 Hz, 3H). ^13^C NMR (101 MHz, CDCl_3_) δ 167.4, 162.8 (d, *J_C-F_* = 251.0 Hz), 154.3, 145.3, 133.6, 132.4 (d, *J_C-F_* = 3.0 Hz), 130.3 (d, *J_C-F_* = 8.0 Hz), 126.1, 117.1 (d, *J_C-F_* = 23.0 Hz), 116.4, 112.9, 110.0, 105.8, 60.7, 55.1, 53.0, 24.1, 22.9, 14.7, and 14.4. HRMS (ESI) *m/z*: Calcd for C_24_H_28_FN_2_O_3_ (M + H)^+^, 411.2078, found 411.2102.

*Ethyl 1-(3-fluorophenyl)-5-hydroxy-2-methyl-4-(piperidin-1-ylmethyl)-1H-indole-3-carboxylate* (**38**).

This compound was obtained from ethyl acetoacetate (**26**) and 3-fluoroaniline, employing Methods D and C. Overall yield 36%; brown oil. ^1^H NMR (400 MHz, CDCl_3_) δ 7.52 (q, *J* = 7.3 Hz, 1H), 7.21 (t, *J* = 8.3 Hz, 1H), 7.10 (d, *J* = 7.8 Hz, 1H), 7.03 (d, *J* = 9.0 Hz, 1H), 6.79 (d, *J* = 8.7 Hz, 1H), 6.72 (d, *J* = 8.7 Hz, 1H), 4.40 (q, *J* = 6.9 Hz, 2H), 4.19 (s, 2H), 3.17–1.89 (m, 7H), 1.66 (br, 6H), and 1.43 (t, *J* = 7.0 Hz, 3H). ^13^C NMR (101 MHz, CDCl_3_) δ 166.8, 163.0 (d, *J_C-F_* = 250.6 Hz), 155.0, 142.7, 138.3 (d, *J_C-F_* = 9.6 Hz), 132.3, 130.9 (d, *J_C-F_* = 9.2 Hz), 124.8, 124.3 (d, *J_C-F_* = 2.6 Hz), 116.0 (d, *J_C-F_* = 2.6 Hz), 115.8, 113.7, 111.3, 110.0, 106.5, 60.2, 59.2, 53.8, 25.9, 24.0, 14.6, and 13.3. HRMS (ESI) *m/z*: Calcd for C_24_H_28_FN_2_O_3_ (M + H)^+^, 411.2078, found 411.2091.

*Ethyl 1-(2-fluorophenyl)-5-hydroxy-2-methyl-4-(piperidin-1-ylmethyl)-1H-indole-3-carboxylate* (**39**).

This compound was obtained from ethyl acetoacetate (**26**) and 2-fluoroaniline, employing Methods D and C. Overall yield 39%; brown oil. ^1^H NMR (400 MHz, CDCl_3_) δ 7.50 (q, *J* = 6.1 Hz, 1H), 7.38–7.27 (m, 3H), 6.74–6.68 (m, 2H), 4.40 (q, *J* = 7.0 Hz, 2H), 4.28–4.14 (m, 2H), 3.18–1.82 (m, 7H), 1.65 (br, 6H), and 1.43 (t, *J* = 7.0 Hz, 3H). ^13^C NMR (101 MHz, CDCl_3_) δ 166.9, 158.6 (d, *J_C-F_* = 253.6 Hz), 155.1, 143.8, 132.2, 130.9 (d, *J_C-F_* = 7.8 Hz), 130.7, 125.1 (d, *J_C-F_* = 4.2 Hz), 125.1, 124.5 (d, *J_C-F_* = 13.2 Hz), 117.3 (d, *J_C-F_* = 19.2 Hz), 113.7, 111.6, 109.9, 106.7, 60.3, 59.3, 53.9, 26.1, 24.2, 14.7, and 13.1. HRMS (ESI) *m/z*: Calcd for C_24_H_28_FN_2_O_3_ (M + H)^+^, 411.2078, found 411.2108.

*(Z)-4-((4-bromophenyl)amino)pent-3-en-2-one* (**42**).

This compound was obtained from pentane-2,4-dione (**40**) and 4-bromoaniline (**41**), employing Method A. Overall yield 72%; yellow oil. ^1^H NMR (400 MHz, CDCl_3_) δ 12.43 (s, 1H), 7.45 (d, *J* = 8.6 Hz, 2H), 6.98 (d, *J* = 8.6Hz, 2H), 5.21 (s, 1H), 2.10 (s, 3H), and 1.99 (s, 3H). ^13^C NMR (101 MHz, CDCl_3_) δ 191.3, 156.1, 137.5, 132.2, 126.2, 118.8, 98.3, 29.3, and 19.7.

*1-(1-(4-bromophenyl)-5-hydroxy-2-methyl-1H-indol-3-yl)ethan-1-one*(**43**).

This compound was obtained from Compound **42**, employing Method B. Overall yield 67%; brown solid.^1^H NMR (400 MHz, DMSO-*d_6_*) δ 9.12 (s, 1H), 7.83 (d, *J* = 8.2 Hz, 2H), 7.50 (s, 1H), 7.42 (d, *J* = 8.2 Hz, 2H), 6.80 (d, *J* = 8.8 Hz, 1H), 6.65 (d, *J* = 8.8 Hz, 1H), 2.55 (s, 3H), and 2.49 (s, 4H). ^13^C NMR (101 MHz, DMSO-*d_6_*) δ 193.4, 153.5, 144.2, 135.3, 133.0, 131.3, 130.4, 127.1, 122.0, 114.4, 112.1, 110.9, 105.7, 39.9, 39.7, 39.5, 39.3, 39.1, 31.3, and 13.8.

*1-(1-([1,1′-biphenyl]-4-yl)-5-hydroxy-2-methyl-1H-indol-3-yl)ethan-1-one* (**44**).

To a solution of Compound **43** (1mmol) in toluene: EtOH: H_2_O (3:2:1) (12 mL) under N_2_, were added 3 M aqueous solution of K_2_CO_3_ (18.88 mmol), phenylboronic acid (11.62 mmol), and Pd(PPh_3_)_4_ (0.29 mmol). The mixture was stirred at 100 °C for 4 h. After cooling to r.t. The resulting suspension was diluted with EtOAc (20 mL), the aqueous layer was extracted with EtOAc (30mL), and the combined organic layers washed with NaCl aqueous solution. The residue was purified by flash chromatography (dichloromethane: methanol = 40:1) to afford **44**. Yield 42%; pale yellow solid. ^1^H NMR (400 MHz, DMSO-*d*_6_) δ 9.11 (s, 1H), 7.92 (d, *J* = 7.6 Hz, 2H), 7.79 (d, *J* = 7.6 Hz, 2H), 7.67–7.59 (m, 3H), 7.57–7.49 (m, 6H), 7.43 (t, *J* = 7.2 Hz, 1H), 6.85 (d, *J* = 8.8 Hz, 1H), 6.66 (d, *J* = 8.8 Hz, 1H), 2.56 (s, 3H), and 2.54 (s, 3H). ^13^C NMR (101 MHz, DMSO-*d*_6_) δ 193.0, 153.0, 144.0, 140.1, 138.6, 134.7, 131.7, 131.1, 128.7, 128.3, 128.2, 127.7, 126.5, 115.2, 113.8, 111.6, 105.2, 30.8, and 13.5.

*1-(1-([1,1′-biphenyl]-4-yl)-5-hydroxy-2-methyl-4-(piperidin-1-ylmethyl)-1H-indol-3-yl)ethan-1-one* (**45**).

This compound was obtained from Compound **44**, employing Method C. Overall yield 83%; pale yellow solid. ^1^H NMR (400 MHz, CD_3_OD:CDCl_3_ = 1:2) δ 7.82 (d, *J* = 7.8 Hz, 2H), 7.65 (d, *J* = 7.6 Hz, 2H), 7.50–7.47 (m, 3H), 7.35 (d, *J* = 7.8 Hz, 2H), 6.95 (d, *J* = 8.9 Hz, 1H), 6.84 (d, *J* = 8.7 Hz, 1H), 4.48 (s, 2H), 3.68–3.50 (m, 2H), 3.18–3.03 (m, 2H), 2.68 (s, 3H), 2.58 (s, 3H), 2.14–2.01 (m, 2H), 1.87–1.73 (m, 2H), and 1.68–1.51 (m, 2H). ^13^C NMR (101 MHz, CD_3_OD:CDCl_3_ = 1:2) δ 199.8, 154.8, 146.7, 143.5, 139.9, 135.0, 134.0, 129.5, 129.3, 129.1, 128.7, 127.6, 126.2, 117.7, 115.1, 113.3, 106.4, 53.5, 53.1, 31.9, 24.4, 22.4, and 15.6. HRMS (ESI) *m/z*: Calcd for C_29_H_31_N_2_O_2_ (M + H)^+^, 439.2380, found 439.2386.

*4-(piperidin-1-yl) aniline* (**47**).

To a solution of indole *P*-nitroaniline (1 mol) in DMSO (30 mL) was added K_2_CO_3_ and piperidine (4 mol) at room temperature under N_2_. The reaction mixture was allowed to 90 °C for 8–12 h and then cooled to rt. The reaction mixture was extracted with ethyl acetate (3 × 20 mL), washed with NaCl aqueous solution, and the combined organic layer was dried with anhydrous Na_2_SO_4_. The residue was evaporated and purified by flash chromatography (petroleum ether: ethyl acetate = 20:1). Then, performed catalytic hydrogenation without further purification. Overall yield 90%; yellow solid. ^1^H NMR (400 MHz, CDCl_3_) δ 6.83 (d, *J* = 8.0 Hz, 2H), 6.64 (d, *J* = 8.0 Hz, 2H), 3.32 (s, 2H), 3.01–2.95 (m, 4H), 1.77–1.65 (m, 4H), and 1.58–1.47 (m, 2H). ^13^C NMR (101 MHz, CDCl_3_) δ 146.0, 139.9, 119.3, 116.3, 52.7, 26.3, and 24.3.

*1-(5-hydroxy-2-methyl-1-(4-(piperidin-1-yl) phenyl)-4-(piperidin-1-ylmethyl)-1H-indol-3-yl) ethan-1-one* (**48**).

This compound was obtained from pentane-2,4-dione (**40**) and Compound **47**, employing Methods A, B, and C. Overall yield 41%; brown solid. ^1^H NMR (400 MHz, CDCl_3_) δ 7.30 (d, *J* = 8.7 Hz, 1H), 7.13–6.95 (m, 4H), 6.79 (d, *J* = 8.7 Hz, 1H), 4.63 (s, 2H), 3.56–3.41 (m, 2H), 3.35–3.20 (m, 4H), 3.14–2.95 (m, 2H), 2.60 (s, 3H), 2.45 (s, 3H), and 2.00–1.55 (m, 12H). ^13^C NMR (101 MHz, DMSO-*d_6_)* δ 196.2, 152.8, 144.9, 132.2, 128.1, 125.3, 116.2, 115.3, 113.0, 111.7, 110.4, 110.1, 106.3, 51.7, 51.6, 48.2, 31.3, 24.5, 23.2, 21.9, 20.7, and 14.2. HRMS (ESI) *m/z*: Calcd for C_28_H_36_N_3_O_2_ (M + H)^+^, 446.2802, found 446.2835.

*Ethyl 1-(4-(dimethylamino)phenyl)-5-hydroxy-2-methyl-4-(piperidin-1-ylmethyl)-1H-indole-3-carboxylate* (**49**).

This compound was obtained from ethyl acetoacetate (**26**) and *N,N-*dimethyl*-1,4-*phenylenediamine, employing Methods A, B, and C. Overall yield 33%; brown solid. ^1^H NMR (400 MHz, CD_3_OD:CDCl_3_ = 1:3) δ 6.98 (d, *J* = 8.0 Hz, 2H), 6.85–6.80 (m, 2H), 6.73 (d, *J* = 8.1 Hz, 2H), 4.58 (s, 2H), 4.32 (q, *J* = 7.0 Hz, 2H), 3.40 (s, 2H), 3.11–2.87 (m, 8H), 2.38 (s, 3H), 2.04–1.56 (m, 6H), and 1.35 (t, *J* = 7.0 Hz, 3H). ^13^C NMR (101 MHz, CD_3_OD:CDCl_3_ = 1:3) δ 168.9, 153.8, 150.9, 147.4, 133.8, 128.8, 126.1, 124.0, 114.5, 112.7, 112.5, 105.4, 104.4, 61.1, 53.8, 52.6, 40.5, 24.0, 22.2, and 14.5. HRMS (ESI) *m/z*: Calcd for C_26_H_34_N_3_O_3_ (M + H)^+^, 436.2595, found 436.2625.

*1-(1-(4-(dimethylamino)phenyl)-5-hydroxy-2-methyl-4-(piperidin-1-ylmethyl)-1H-indol-3-yl)ethan-1-one* (**50**).

This compound was obtained from pentane-2,4-dione (**40**) and *N,N-*dimethyl*-1,4-*phenylenediamine, employing Methods A, B, and C. Overall yield 37%; pale brown solid. ^1^H NMR (400 MHz, CD_3_OD:CDCl_3_ = 1:1) δ 7.06 (d, *J* = 8.0 Hz, 2H), 6.80 (d, *J* = 8.0 Hz, 2H), 6.74 (d, *J* = 8.6 Hz, 1H), 6.64 (d, *J* = 8.6 Hz, 1H), 4.05 (s, 2H), 3.01 (s, 6H), 2.81–2.53 (m, 4H), 2.37 (s, 3H), and 1.72–1.45 (m, 6H). ^13^C NMR (101 MHz, CD_3_OD:CDCl_3_ = 1:1) δ 199.2, 155.0, 151.3, 143.9, 134.1, 129.3, 125.3, 125.1, 117.4, 113.8, 113.2, 111.7, 111.3, 59.4, 54.0, 40.7, 26.2, 24.2, and 14.3. HRMS (ESI) *m/z*: Calcd for C_25_H_32_N_3_O_2_ (M + H)^+^, 406.2489, found 406.2523.

*1-(4-((dimethylamino)methyl)-1-(4-(dimethylamino)phenyl)-5-hydroxy-2-methyl-1H-indol-3-yl)ethan-1-one* (**51**).

This compound was obtained from pentane-2,4-dione (**40**), *N,N-*dimethyl*-1,4-*phenylenediamine, and *N, N*-dimethylamine, employing Methods A, B, and C. Overall yield 34%; dark brown solid. ^1^H NMR (400 MHz, CD_3_OD:CDCl_3_ = 1:2) δ 7.05 (d, *J* = 8.3 Hz, 2H), 6.87 (d, *J* = 8.8 Hz, 1H), 6.83–6.77 (m, 3H), 4.62 (s, 2H), 3.03 (s, 6H), 2.92 (s, 6H), 2.61 (s, 3H), and 2.49 (s, 3H). ^13^C NMR (101 MHz, CD_3_OD:CDCl_3_ = 1:2) δ 198.0, 154.1, 151.2, 147.0, 134.3, 128.9, 125.9, 123.7, 116.8, 114.9, 112.9, 112.8, 107.1, 54.8, 42.71, 40.5, 31.8, and 15.4. HRMS (ESI) *m/z*: Calcd for C_22_H_28_N_3_O_2_ (M + H)^+^, 366.2176, found 366.2199.

*1-(1-(4-(dimethylamino)phenyl)-5-hydroxy-2-methyl-4-(pyrrolidin-1-ylmethyl)-1H-indol-3-yl)ethan-1-one* (**52**).

This compound was obtained from pentane-2,4-dione (**40**), *N,N*-dimethyl-1,4-phenylenediamine, and pyrrolidine, employing Methods A, B, and C. Overall yield 37%; brown solid. ^1^H NMR (400 MHz, CDCl_3_) δ 7.08 (d, *J* = 8.5 Hz, 2H), 7.00 (d, *J* = 8.7 Hz, 1H), 6.85–6.77 (m, 3H), 4.66 (s, 2H), 3.23–3.08 (m, 4H), 3.05 (s, 6H), 2.60 (s, 3H), 2.45 (s, 3H), and 2.06–1.97 (m, 4H). ^13^C NMR (101 MHz, CDCl_3_) δ 197.5, 154.2, 150.7, 144.3, 134.0, 129.0, 125.0, 124.3, 117.0, 115.2, 112.8, 112.7, 111.1, 53.4, 53.3, 40.6, 32.3, 23.7, and 14.8. HRMS (ESI) *m/z*: Calcd for C_24_H_30_N_3_O_2_ (M + H)^+^, 392.2333, found 392.2361.

*1-(1-(4-(dimethylamino)phenyl)-5-hydroxy-2-methyl-4-((4-methylpiperidin-1-yl)methyl)-1H-indol-3-yl)ethan-1-one* (**53**).

This compound was obtained from pentane-2,4-dione (**40**), *N,N-*dimethyl*-1,4-*phenylenediamine, and 4-methylpiperidine, employing Methods A, B, and C. Overall yield 31%; brown solid. ^1^H NMR (400 MHz, CD_3_OD:CDCl_3_ = 1:2) δ 7.08 (d, *J* = 8.0 Hz, 2H), 6.90–6.82 (m, 3H), 6.77 (d, *J* = 8.7 Hz, 1H), 4.45 (s, 2H), 3.54–3.42 (m, 2H), 3.14–2.88 (m, 9H), 2.68–2.60 (m, 2H), 2.50 (s, 3H), 2.01–1.90 (m, 2H), 1.81–1.67 (m, 1H), 1.57–1.39 (m, 2H), and 1.02 (d, *J* = 6.0 Hz, 3H). ^13^C NMR (101 MHz, CD_3_OD:CDCl_3_ = 1:2) δ 199.3, 155.1, 151.4, 143.9, 134.2, 129.4, 125.3, 125.2, 117.6, 113.9, 113.3, 111.7, 111.6, 59.3, 53.6, 40.8, 34.7, 31.1, 31.0, 22.0, and 14.4. HRMS (ESI) *m/z*: Calcd for C_26_H_34_N_3_O_2_ (M + H)^+^, 420.2669, found 420.2646.

*1-(5-hydroxy-1-(4-isopropylphenyl)-2-methyl-4-(piperidin-1-ylmethyl)-1H-indol-3-yl)ethan-1-one* (**54**).

This compound was obtained from pentane-2,4-dione (**40**) and 4-isopropylaniline, employing Methods A, B, and C. Overall yield 41%; brown solid. ^1^H NMR (400 MHz, CDCl_3_) δ 7.39 (d, *J* = 7.8 Hz, 2H), 7.17 (d, *J* = 7.8 Hz, 2H), 6.94 (d, *J* = 8.5 Hz, 1H), 6.80 (d, *J* = 8.7 Hz, 1H), 4.29 (s, 2H), 3.24–2.69 (m, 5H), 2.60 (s, 3H), 2.41 (s, 3H), 1.83–1.56 (m, 6H), and 1.32 (d, *J* = 6.8 Hz, 6H). ^13^C NMR (101 MHz, CDCl_3_) δ 198.3, 154.9, 150.1, 142.6, 133.9, 133.2, 128.2, 127.9, 125.1, 117.5, 114.7, 112.1, 109.8, 57.2, 53.3, 34.02, 32.27, 25.12, 24.02, 23.36, and 14.42. HRMS (ESI) *m/z*: Calcd for C_26_H_33_N_2_O_2_ (M + H)^+^, 405.2537, found 405.2564.

*1-(1-(4-(tert-butyl)phenyl)-5-hydroxy-2-methyl-4-(piperidin-1-ylmethyl)-1H-indol-3-yl)ethan-1-one* (**55**).

This compound was obtained from pentane-2,4-dione (**40**) and 4-(*tert*-butyl)aniline, employing Methods A, B, and C. Overall yield 45%; brown solid. ^1^H NMR (400 MHz, CDCl_3_) δ 7.54 (d, *J* = 8.0 Hz, 2H), 7.20 (d, *J* = 8.3 Hz, 2H), 6.79 (d, *J* = 8.8 Hz, 1H), 6.71 (d, *J* = 8.7 Hz, 1H), 4.00 (s, 2H), 2.83–2.20 (m, 10H), 1.64 (s, 6H), and 1.39 (s, 9H). ^13^C NMR (101 MHz, CDCl_3_) δ 198.0, 154.8, 151.7, 140.6, 133.8, 132.6, 127.7, 126.5, 124.6, 117.5, 113.5, 111.1, 110.3, 59.4, 53.5, 34.7, 32.2, 31.3, 25.8, 23.9, and 13.5. HRMS (ESI) *m/z*: Calcd for C_27_H_35_N_2_O_2_ (M + H)^+^, 419.2693, found 419.2714.

*1-(1-(4-(tert-butyl)phenyl)-5-hydroxy-2-methyl-4-(pyrrolidin-1-ylmethyl)-1H-indol-3-yl)ethan-1-one* (**56**).

This compound was obtained from pentane-2,4-dione (**40**), 4-(*tert*-butyl)aniline, and pyrrolidine, employing Methods A, B and C. Overall yield 42%; brown solid. ^1^H NMR (400 MHz, CD_3_OD:CDCl_3_ = 1:3) δ 7.53 (d, *J* = 7.6 Hz, 2H), 7.12 (d, *J* = 7.6 Hz, 2H), 6.84–6.79 (m, 2H), 4.66 (s, 2H), 3.58–3.31 (m, 4H), 2.59 (s, 3H), 2.46 (s, 3H), 2.25–2.02 (m, 4H), and 1.33 (s, 9H). ^13^C NMR (101 MHz, CD_3_OD:CDCl_3_ = 1:3) δ 198.4, 153.8, 153.3, 146.2, 133.8, 133.1, 128.0, 127.3, 125.8, 117.2, 114.6, 113.1, 107.8, 53.6, 51.5, 35.2, 31.9, 31.4, 23.5, and 15.5. HRMS (ESI) *m/z*: Calcd for C_26_H_33_N_2_O_2_ (M + H)^+^, 405.2537, found 405.2563.

*Ethyl 1-(4-(tert-butyl)phenyl)-5-hydroxy-2-methyl-4-(piperidin-1-ylmethyl)-1H-indole-3-carboxylate* (**57**).

This compound was obtained from ethyl acetoacetate (**26**) and 4-(*tert*-butyl)aniline, employing Methods A, B, and C. Overall yield 33%; brown solid. ^1^H NMR (400 MHz, CDCl_3_) δ 7.53 (d, *J* = 7.8 Hz, 2H), 7.19 (d, *J* = 7.8 Hz, 2H), 6.79 (d, *J* = 8.7 Hz, 1H), 6.70 (d, *J* = 8.7 Hz, 1H), 4.40 (q, *J* = 7.0 Hz, 2H), 4.22 (s, 2H), 3.01–1.99 (m, 7H), 1.60 (br, 6H), and 1.39 (s, 9H). ^13^C NMR (101 MHz, CDCl_3_) δ 167.0, 154.8, 151.8, 143.5, 134.1, 132.7, 127.8, 126.6, 124.8, 113.3, 111.2, 110.4, 105.8, 60.1, 59.3, 53.8, 34.9, 31.4, 26.0, 24.1, 14.7, and 13.5. HRMS (ESI) *m/z*: Calcd for C_28_H_37_N_2_O_3_ (M + H)^+^, 449.2799, found 449.2814.

*1-(1-(4-bromophenyl)-5-hydroxy-2-methyl-4-(piperidin-1-ylmethyl)-1H-indol-3-yl)ethan-1-one* (**58**).

This compound was obtained from Compound **43**, employing Method C. Overall yield 84%; pale yellow solid. ^1^H NMR (400 MHz, CD_3_OD:CDCl_3_ = 1:1) δ 7.73 (d, *J* = 7.7 Hz, 2H), 7.17 (d, *J* = 7.7 Hz, 2H), 6.86–6.81 (m, 2H), 4.41 (s, 2H), 3.68–3.34 (m, 2H), 3.26–2.83 (m, 2H), 2.64 (s, 3H), 2.49 (s, 3H), and 2.08–1.57 (m, 6H). ^13^C NMR (101 MHz, CD_3_OD:CDCl_3_ = 1:1) δ 199.7, 154.8, 145.3, 135.1, 133.8, 133.5, 130.3, 125.9, 124.2, 117.9, 114.1, 113.5, 107.0, 54.2, 53.1, 32.0, 24.5, 22.5, and 15.1. HRMS (ESI) *m/z*: Calcd for C_23_H_26_BrN_2_O_2_ (M + H)^+^, 441.1172, found 441.1194.

### 4.2. General Procedures for Biological Studies

As reported in the literature, MIC was determined using the MABA assay [23]. *Nano differential scanning fluorimetry (nano DSF) assay*. *T*_m_ was measured by using nano DSF, as described previously [14]. Diluted Pks13-TE protein samples to 30 µM were incubated with tested compounds (in DMSO) in a 30 µL reaction volume for 30 min using a heating gradient of 2 °C per minute over a temperature range from 30 °C to 90 °C. The changes in intrinsic fluorescence intensity ratio (350:330 nm) were measured, plotting the fluorescence intensity ratio (or the first derivative of the ratio) as nano DSF thermogram. The thermal transition temperature (*T*_m_) is obtained in the post-run data analysis. *Liver microsome stability assay.* The assay was performed with liver microsomes from human. The incubation was performed as follows: microsomes in 0.01 M phosphate buffer pH 7.4 (0.56 mg/mL microsomal protein), tested compounds (final concentration 0.1 µM, cosolvent (DMSO)), and then NADPH (1 mM) at 37 °C with constant shaking for 60 min. The reaction can be started by adding NADPH or the same volume buffer. Aliquots were sampled at 5, 15, 30, 45, and 60 min incubation, and enzymatic reaction was quenched by addition of acetonitrile. After centrifugation, samples were then analyzed by LC-MS/MS. The assay evaluated the metabolic stability of compounds by measuring the substrate remaining with or without NADPH cofactor.

## 5. Conclusions

Pks13 is a promising target for the development of novel anti-TB drugs. This report has synthesized a series of *N*-phenylindole derivatives targeting Pks13-TE based on a structure-guided strategy. The SAR studies showed that the nitrogen of indole substituted by phenyl was favorable for activity. Further exploration demonstrated that introducing hydrophobic groups at the para position of the benzene ring resulted in a significant improvement for antitubercular activity against *Mtb*. At the three-position of *N*-phenylindole derivatives, acetyl substituents are better than the ester group and amide substituents for activity. The rational drug design on the *N*-phenylindole series resulted in the discovery of the potent Compounds **45** and **58**, with an MIC value of 0.0625 µg/mL and 0.125μg/mL, respectively. We further verified that *N*-phenylindole derivatives are bound to the Pks13-TE domain using the nano DSF method, consistent with the observed MIC trends. The preliminary metabolism evaluation of Compound **58** revealed moderate human microsomal stability. Taken together, *N*-phenylindole derivatives as a novel anti-TB scaffold have the promise to warrant the further development of lead compounds.

## Figures and Tables

**Table 1:** Antitubercular activity of Compounds **9**, **13–20**, and **22–25** against the *Mtb* Strain H37Rv ^a^.

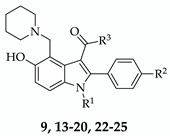					
**ID**	**R^1^**	**R^2^**	**R^3^**	**MIC ^a^ µg/mL**	**CLogP ^b^**
**9** ^c^	H	H	OCH_2_CH_3_	32	5.57
**13**	H	OCH_3_	OCH_2_CH_3_	32	5.50
**14**	H	OH	OCH_2_CH_3_	16	4.91
**15**	CH_3_	OH	OCH_2_CH_3_	32	5.37
**16**	*i*Pr	OH	OCH_2_CH_3_	16	6.21
**17**	Ph	OCH_3_	OCH_2_CH_3_	4	8.03
**18**	Ph	OH	OCH_2_CH_3_	2	7.45
**19**	Ph	OCH_3_	CH_3_	4	6.99
**20**	Ph	OH	CH_3_	16	6.41
**22**	Ph	OCH_3_	*N*HCH_3_	16	5.69
**23**	Ph	OCH_3_	*N*HCH_2_CH_3_	16	6.22
**24**	Ph	OH	*N*HCH_3_	4	5.11
**25**	Ph	OH	*N*HCH_2_CH_3_	8	5.64

^a^ The lowest concentration of compounds leading to at least 90% inhibition of bacterial growth signal by the MABA. MIC values are reported as an average of three individual measurements. ^b^ CLogP was calculated using ChemBioDraw Ultra 16.0. ^c^ Compound **9** was a reference compound for comparison [14].

**Table 2:** Antitubercular activity of Compounds **28–39**, **45**, and **48–58** against the *Mtb* Strain H37Rv ^a^.

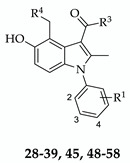					
**ID**	**R^1^**	**R^3^**	**R^4^**	**MIC ^a^ µg/mL**	**CLogP ^b^**
**28**	H	OCH_2_CH_3_	piperidyl	8	6.50
**29**	H	*N*HCH_3_	piperidyl	>64	4.29
**30**	4-OCH_3_	*N*HCH_3_	piperidyl	16	4.23
**31**	4-CH_3_	OCH_2_CH_3_	piperidyl	1	7.00
**32**	3-CH_3_	OCH_2_CH_3_	piperidyl	4	7.00
**33**	2-CH_3_	OCH_2_CH_3_	piperidyl	4	7.00
**34**	4-OCH_3_	OCH_2_CH_3_	piperidyl	1	6.48
**35**	3-OCH_3_	OCH_2_CH_3_	piperidyl	8	6.48
**36**	2-OCH_3_	OCH_2_CH_3_	piperidyl	8	6.48
**37**	4-F	OCH_2_CH_3_	piperidyl	1	6.66
**38**	3-F	OCH_2_CH_3_	piperidyl	8	6.66
**39**	2-F	OCH_2_CH_3_	piperidyl	4	6.66
**45**	4-phenyl	CH_3_	piperidyl	0.0625	7.36
**48**	4-piperidyl	CH_3_	piperidyl	0.125	6.35
**49**	4-*N, N-*dimethyl	OCH_2_CH_3_	piperidyl	1	6.73
**50**	4-*N, N-*dimethyl	CH_3_	piperidyl	0.5	5.67
**51**	4-*N, N-*dimethyl	CH_3_	*N, N-*dimethyl	2	4.48
**52**	4-*N, N-*dimethyl	CH_3_	pyrrolidinyl	0.5	5.12
**53**	4-*N, N-*dimethyl	CH_3_	4-methylpiperidyl	1	6.19
**54**	4-*i-*propyl	CH_3_	piperidyl	0.125	6.90
**55**	4-*t-*butyl	CH_3_	piperidyl	0.125	7.30
**56**	4-*t*-butyl	CH_3_	pyrrolidinyl	0.25	6.74
**57**	4-*t*-butyl	OCH_2_CH_3_	piperidyl	0.5	8.33
**58**	4-Br	CH_3_	piperidyl	0.125	6.35

^a^ The lowest concentration of compounds leading to at least 90% inhibition of bacterial growth signal by the MABA. MIC values are reported as an average of three individual measurements. ^b^ CLogP were calculated using ChemBioDraw Ultra 16.0.

**Table 3:** Thermal stabilization of Pks13-TE upon binding with compounds measured by nano DSF.

ID	Δ*T*_m_ (°C) ^a^		MIC ^b^, µg/mL
	5×	10×	
DMSO	−1.3 ± 0.01		-
TAM16 ^c^	8.7 ± 0.02	7.7 ± 0.10	0.0313
**7 ^c^**	11.4 ± 0.03	10.5 ± 0.01	0.0039
**14**	5.5 ± 0.04	4.9 ± 0.02	16
**28**	8.6 ± 0.01	7.8 ± 0.04	8
**45**	14.0 ± 0.04	13.2 ± 0.65	0.0625
**48**	14.6 ± 0.04	14.0 ± 0.01	0.125
**52**	11.4 ± 0.02	10.3 ± 0.04	0.5
**58**	9.5 ± 0.04	7.6 ± 0.01	0.125

^a^ Δ*T*_m_ was calculated as *T*_m_ (Pks13-TE) in the presence of compounds (3% DMSO final concentration) minus *T*_m_ (Pks13-TE) with DMSO only. The final concentration ratios of Pks-TE and compounds were 1:5 and 1:10. *T*_m_ values were calculated as an average of three individual measurements. ^b^ The lowest concentration of compounds leading to at least 90% inhibition of bacterial growth signal by the MABA. MIC values were reported as an average of three individual measurements. ^c^ Compounds TAM 16 and **7** were reference compounds for comparison [11,16].

**Table 4:** Microsomal Stability of Compounds **48** and **58** in Human Liver Microsomes.

Compounds	T_1/2_^a^ (min)	CL_int(mic)_^b^ (µL/min/mg)	CL_int(liver)_^c^ (mL/min/kg)	Remaining (T = 60 min)
**48**	20.7	66.9	60.2	11.3%
**58**	58.0	23.9	21.5	47.3%
Testosterone	13.1	105.8	95.2	4.1%
Diclofenac	5.6	247.9	223.1	0.1%
Propafenone	5.7	244.8	220.3	0.0%

^a^ T_1/2_ is half-life. ^b^ CL_int_(mic) is the intrinsic clearance; CL_int(mic)_ = 0.693 /half-life/mg of microsome protein per mL. ^c^ CL_int(liver)_ = CL_int(mic)_ × mg of microsomal protein/g liver weight × g of liver weight/kg body weight; mg of microsomal protein/g of liver weight: 45 mg/g; liver weight: 20 g/kg.

## Data Availability

Not applicable.

## Supplementary Materials

The following supporting information can be downloaded at: https://www.mdpi.com/article/10.3390/molecules27092844/s1, ^1^H, ^13^C NMR spectra of the synthesized target compounds.

## Sample Availability

Samples of the compounds are not available from the authors.
