# Supplementary material for: Design, Synthesis and Biological Evaluation of N-phenylindole Derivatives as Pks13 Inhibitors againstMycobacterium tuberculosis
Source: Molecules. 2022 Apr 29;27(9):2844. doi: 10.3390/molecules27092844 (PMC9106008; doi:10.3390/molecules27092844)
Supplement: Supplementary file 1 [file molecules-27-02844-s001.zip › molecules-1649846-supplementary.pdf]

Supplementary Materials

Design, Synthesis and Biological Evaluation of *N*-phenylindole Derivatives as Pks13 Inhibitors against *Mycobacterium tuberculosis*

Yanpeng Cai [1,†], Wei Zhang [1,†], Shichun Lun [2,†], Tongtong Zhu [1], Weijun Xu [1], Fan Yang [1], Jie Tang [3], William R. Bishai [2,*] and Lifang Yu [1,*]

[1] Shanghai Engineering Research Center of Molecular Therapeutics and New Drug Development, School of Chemistry and Molecular Engineering, East China Normal University, 3663 North Zhongshan Road, Shanghai 200062, China; caiyanpengdl@163.com (Y.C.); zhangwei@chem.ecnu.edu.cn (W.Z.); zhutongtong816@163.com (T.Z.); xuweijundl@163.com (W.X.); fyang@chem.ecnu.edu.cn (F.Y.)
[2] Center for Tuberculosis Research, Department of Medicine, Division of Infectious Disease, Johns Hopkins School of Medicine, Baltimore, MD 21231-1044, USA; slun1@jhmi.edu
[3] Shanghai Key Laboratory of Green Chemistry and Chemical Process, School of Chemistry and Molecular Engineering, East China Normal University, 3663 North Zhongshan Road, Shanghai 200062, China; jtang@chem.ecnu.edu.cn
* Correspondence: wbishai1@jhmi.edu (W.R.B.); lfyu@sat.ecnu.edu.cn (L.F.Y.)
† These authors contributed equally to this work.

NMR spectra (^{1}H and ^{13}C) of all final compounds S2-S37

Analytical HPLC purities of final compounds S38

Analytical HPLC traces of Compounds **45** and **58** S39-S41

HRMS of Compounds **45** and **58** S42

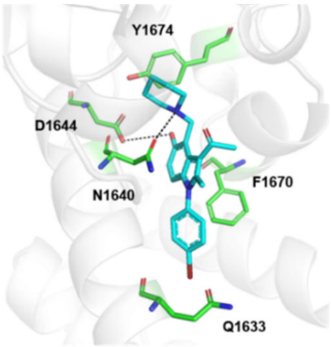

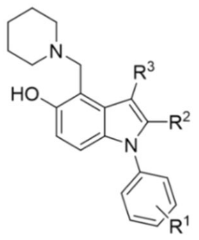

N-phenylindole-based analogues
MIC $_{H37Rv}$: 0.0625 μg/mL

13

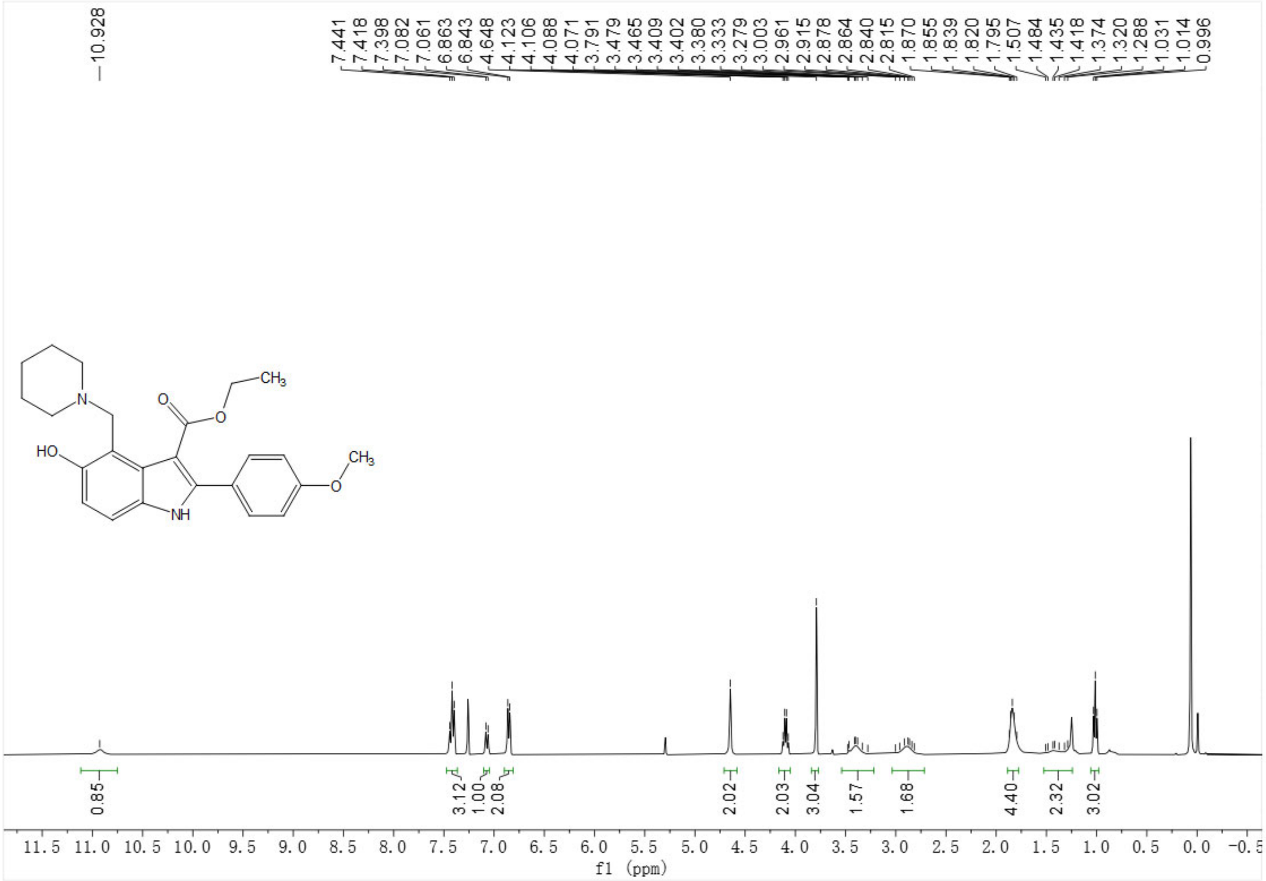
10.928
7.441 7.418 7.398 7.082 7.061 6.863 6.843 4.648 4.123 4.106 4.088 4.071 3.791 3.479 3.465 3.409 3.402 3.380 3.333 3.279 3.003 2.961 2.915 2.878 2.864 2.840 2.815 1.870 1.855 1.839 1.820 1.795 1.507 1.484 1.435 1.418 1.374 1.320 1.288 1.031 1.014 0.996
CH3
O
N
HO
NH
0.85 3.12 1.00 2.08 2.02 2.03 3.04 1.57 1.68 4.40 2.32 3.02
f1 (ppm)

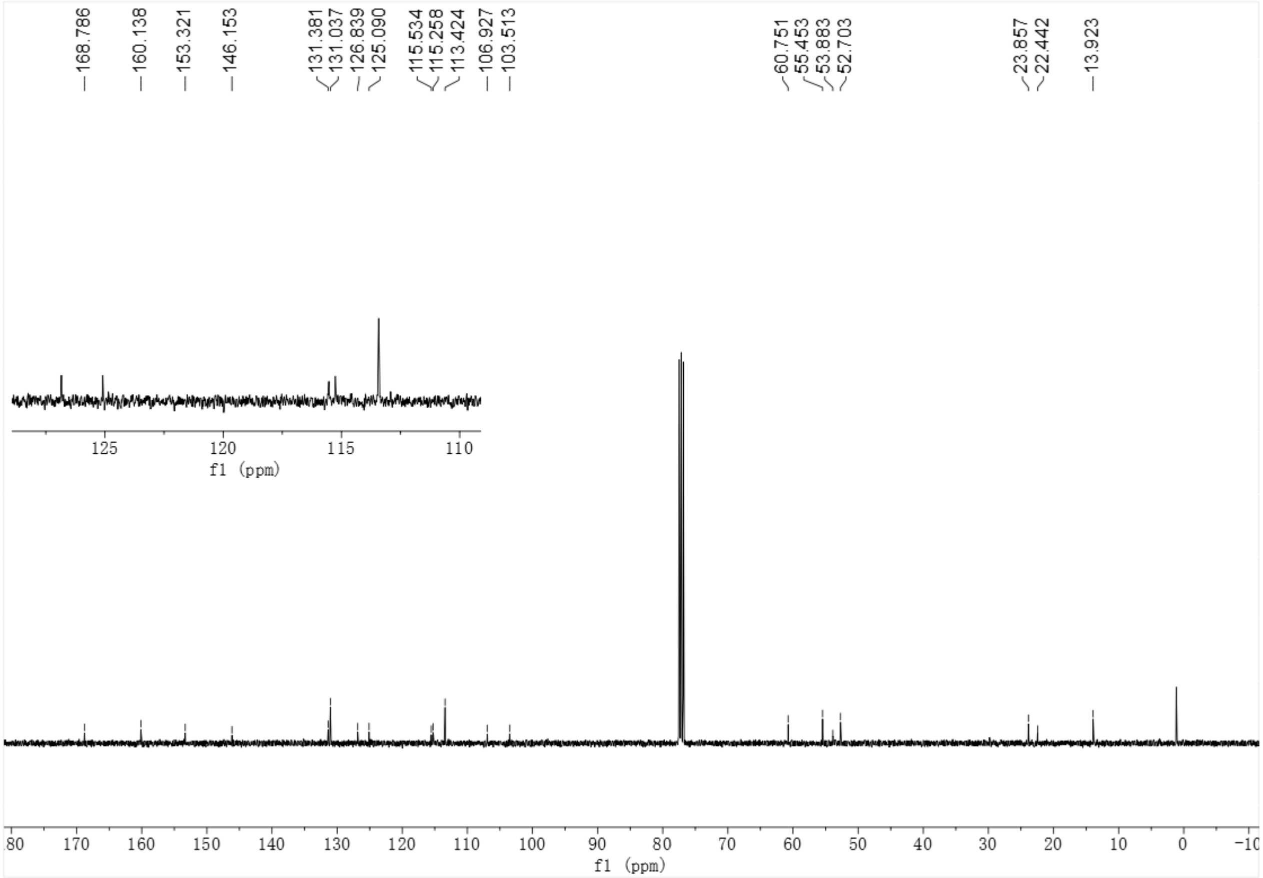
168.786 160.138 153.321 146.153 131.381 131.037 126.839 125.090 115.534 115.258 113.424 106.927 103.513 60.751 55.453 53.883 52.703 23.857 22.442 13.923
f1 (ppm)

14

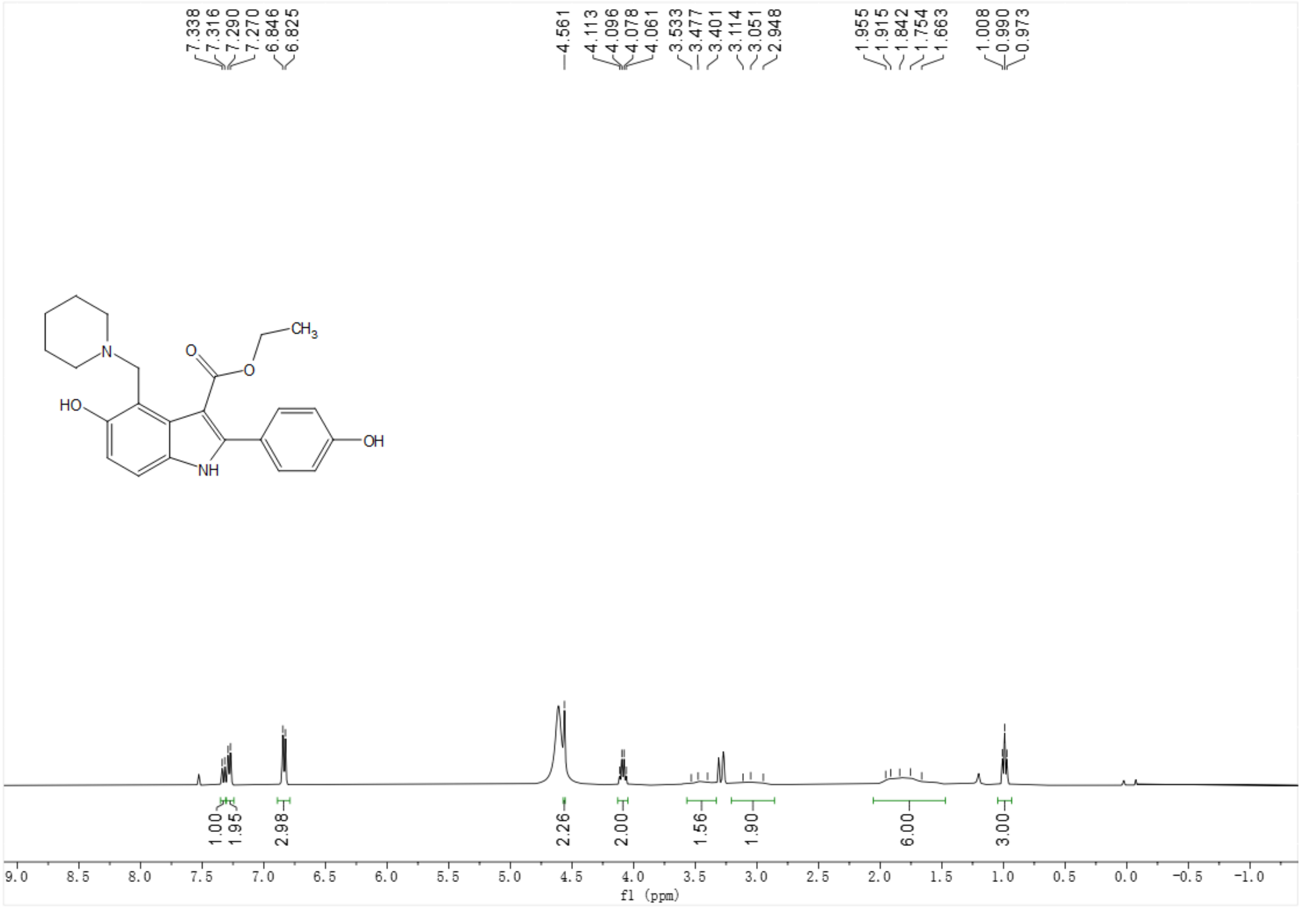
7.338
7.316
7.290
7.270
6.846
6.825
4.561
4.113
4.096
4.078
4.061
3.533
3.477
3.401
3.114
3.051
2.948
1.955
1.915
1.842
1.754
1.663
1.008
0.990
0.973
CH3
O
O
N
HO
OH
NH
1.00
1.95
2.98
2.26
2.00
1.56
1.90
6.00
3.00
f1 (ppm)

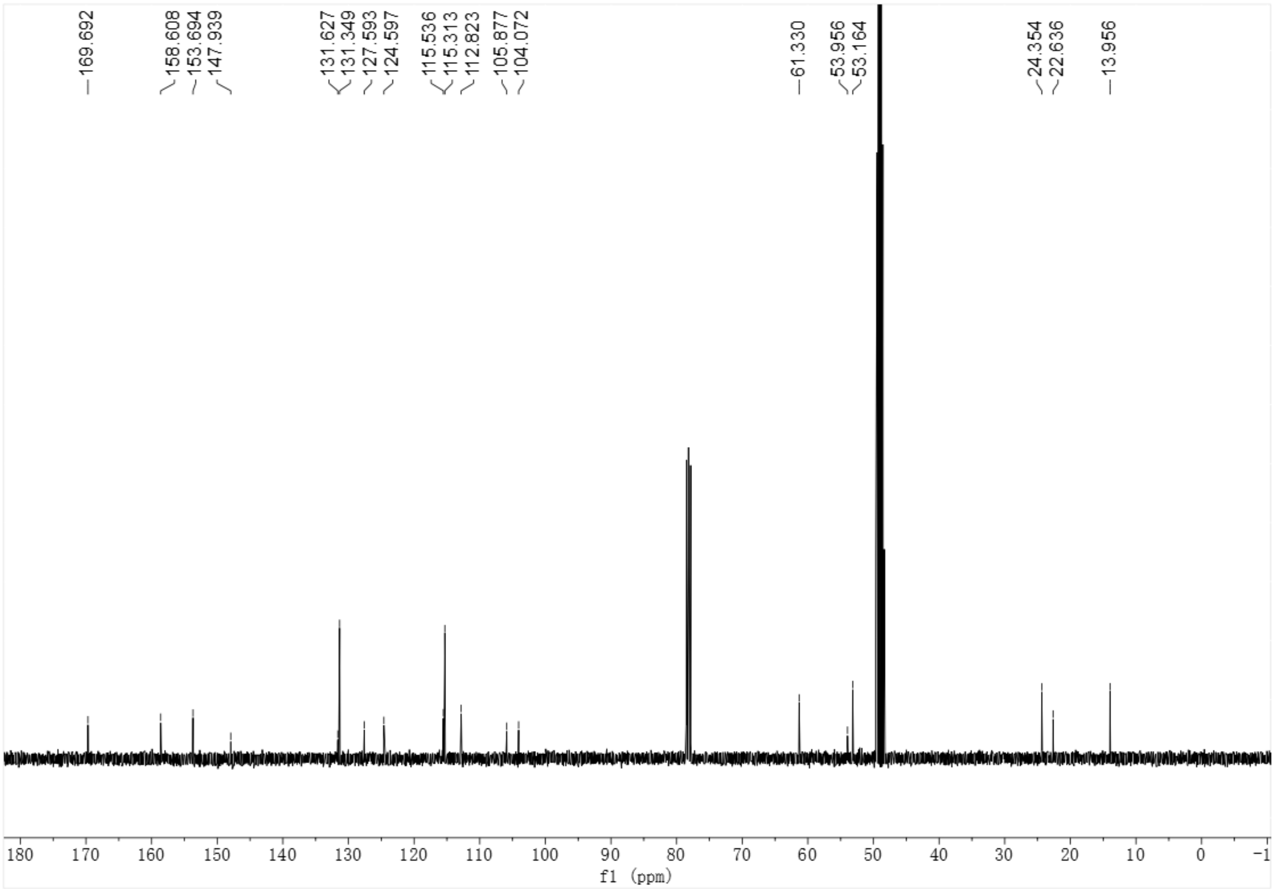
169.692
158.608
153.694
147.939
131.627
131.349
127.593
124.597
115.536
115.313
112.823
105.877
104.072
61.330
53.956
53.164
24.354
22.636
13.956
f1 (ppm)

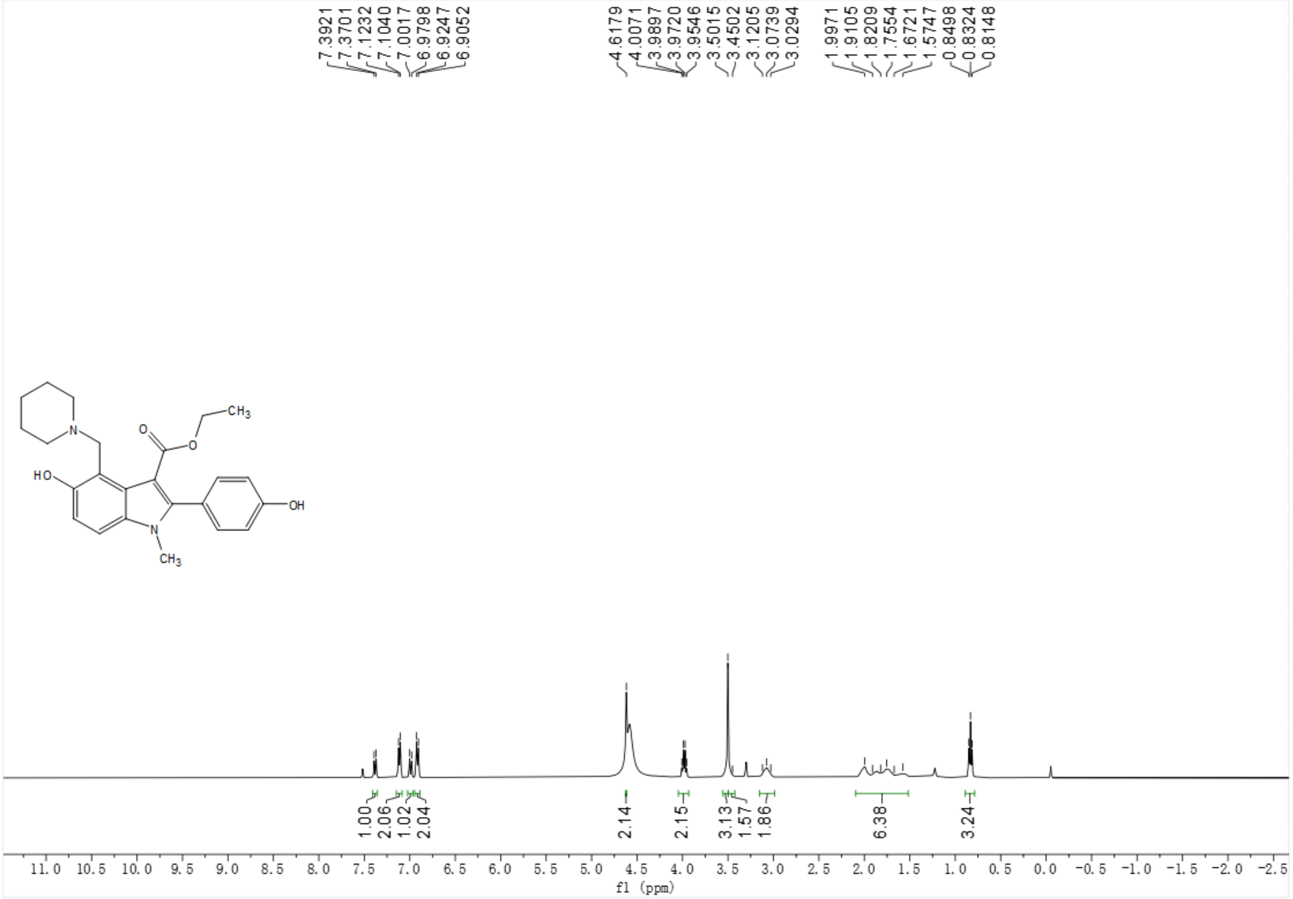
7.3921
7.3701
7.1232
7.1040
7.0017
6.9798
6.9247
6.9052
4.6179
4.0071
3.9897
3.9720
3.9546
3.5015
3.4502
3.1205
3.0739
3.0294
1.9971
1.9105
1.8209
1.7554
1.6721
1.5747
0.8498
0.8324
0.8148
HO
N
O
O
CH3
OH
CH3
1.00
2.06
1.02
2.04
2.14
2.15
3.13
1.57
1.86
6.38
3.24
f1 (ppm)

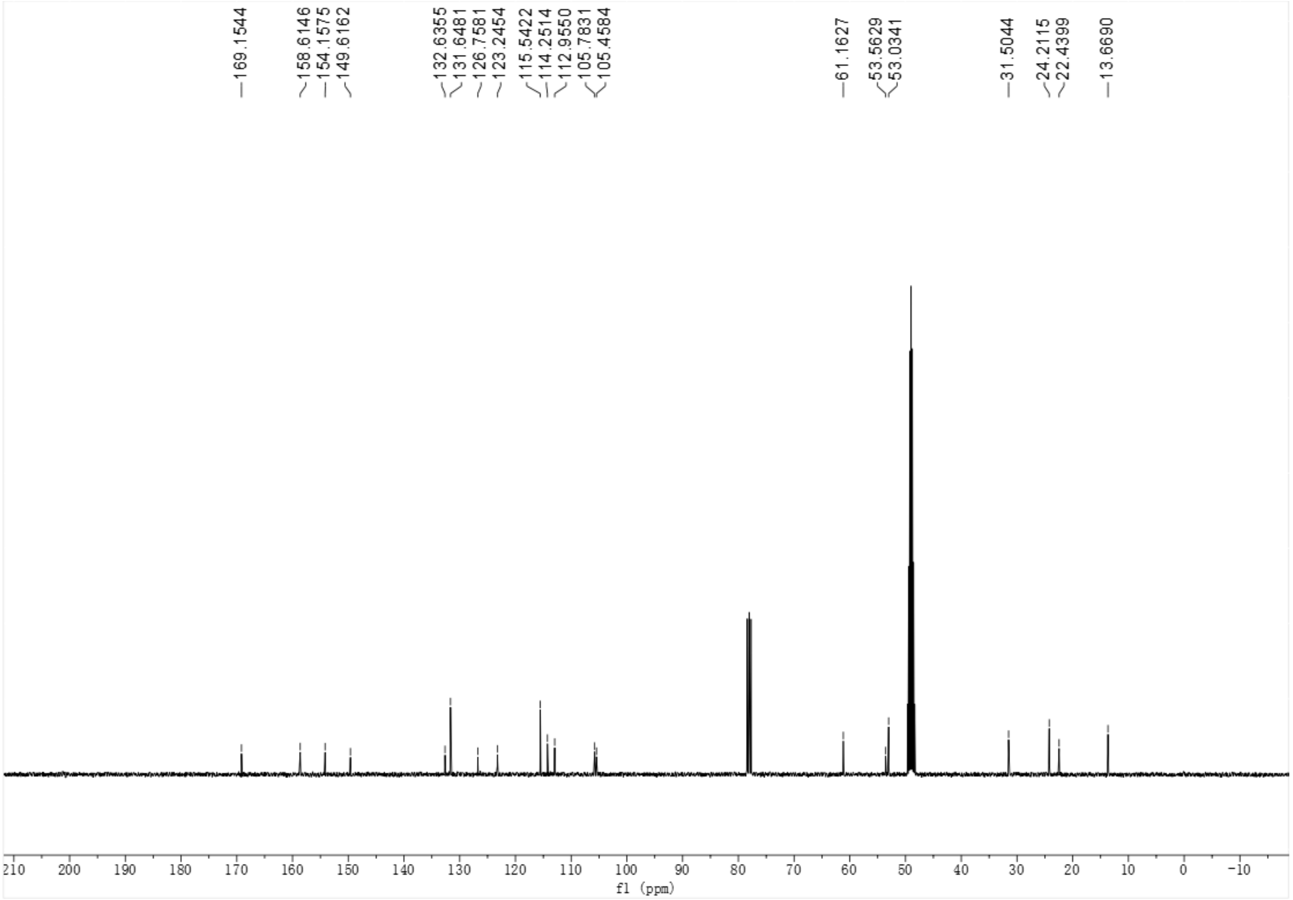
169.1544
158.6146
154.1575
149.6162
132.6355
131.6481
126.7581
123.2454
115.5422
114.2514
112.9550
105.7831
105.4584
61.1627
53.5629
53.0341
31.5044
24.2115
22.4399
13.6690
f1 (ppm)

16

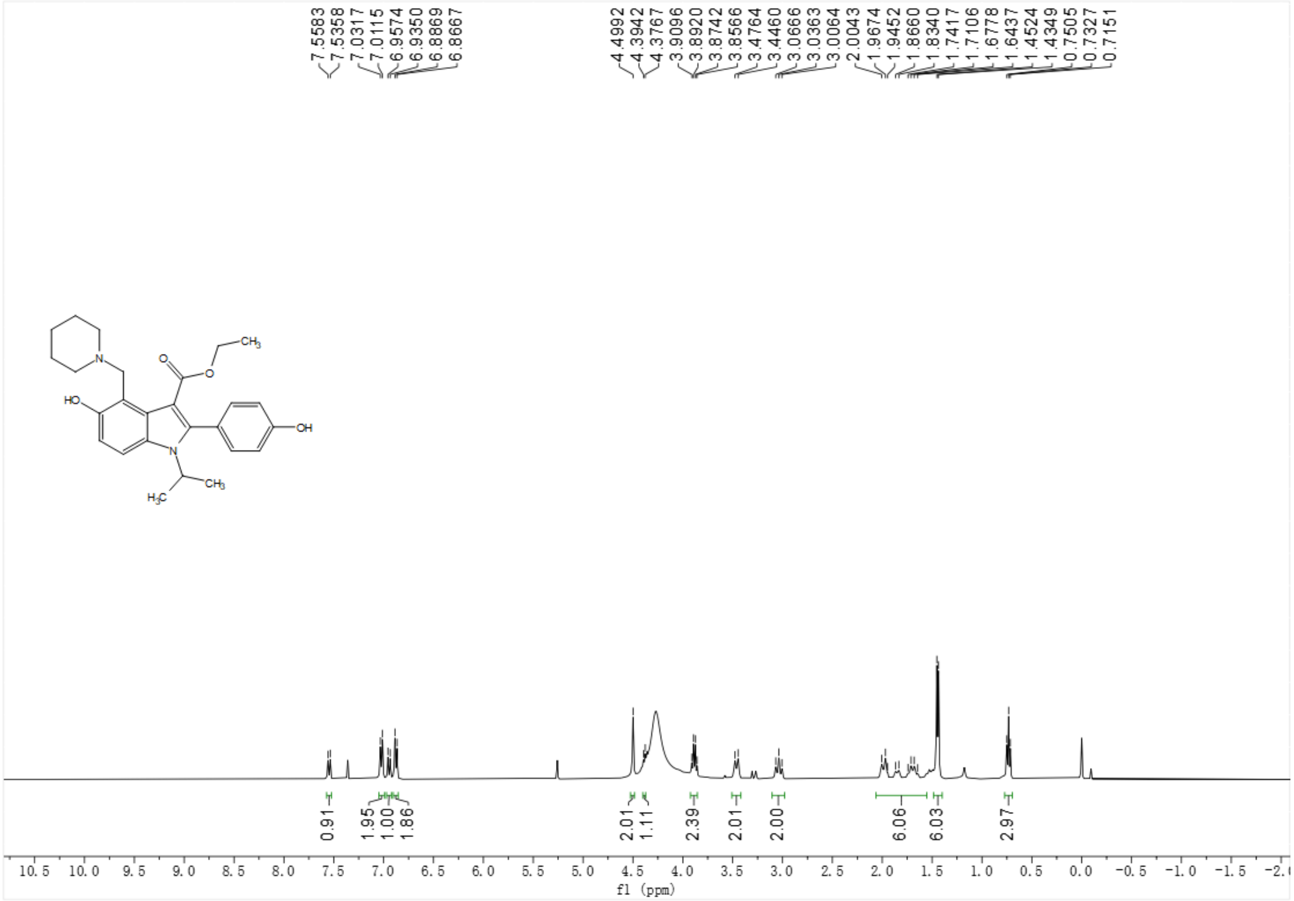
7.5583
7.5358
7.0317
7.0115
6.9574
6.9350
6.8869
6.8667
4.4992
4.3942
4.3767
3.9096
3.8920
3.8742
3.8566
3.4764
3.4460
3.0666
3.0363
3.0064
2.0043
1.9674
1.9452
1.8660
1.8340
1.7417
1.7106
1.6778
1.6437
1.4524
1.4349
0.7505
0.7327
0.7151
HO
OH
O
O
N
N
CH_3
CH_3
H_3C
0.91
1.95
1.00
1.86
2.01
1.11
2.39
2.01
2.00
6.06
6.03
2.97
f1 (ppm)

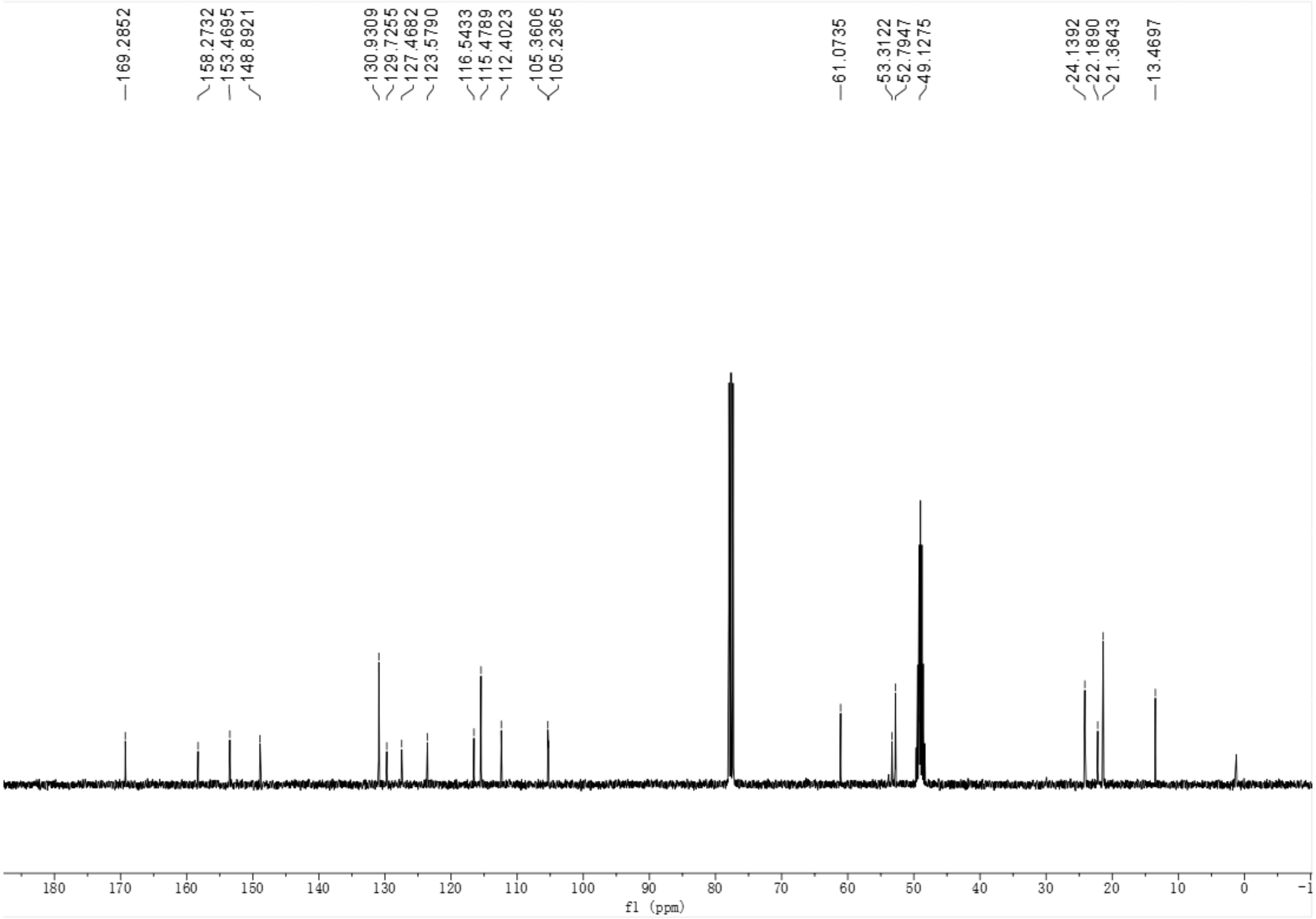
169.2852
158.2732
153.4695
148.8921
130.9309
129.7255
127.4682
123.5790
116.5433
115.4789
112.4023
105.3606
105.2365
61.0735
53.3122
52.7947
49.1275
24.1392
22.1890
21.3643
13.4697
f1 (ppm)

17

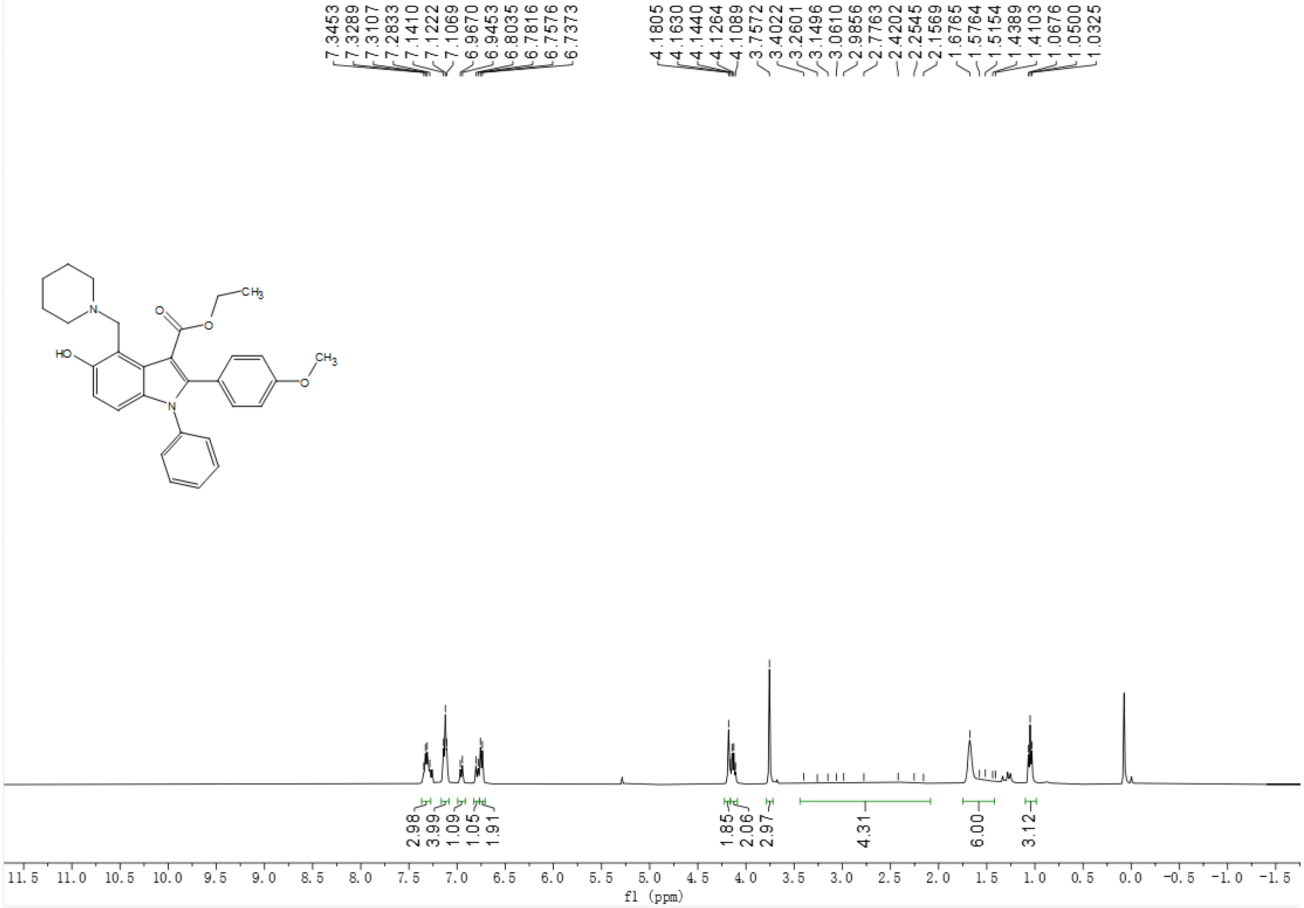
7.3453
7.3289
7.3107
7.2833
7.1410
7.1222
7.1069
6.9670
6.9453
6.8035
6.7816
6.7576
6.7373
4.1805
4.1630
4.1440
4.1264
4.1089
3.7572
3.4022
3.2601
3.1496
3.0610
2.9856
2.7763
2.4202
2.2545
2.1569
1.6765
1.5764
1.5154
1.4389
1.4103
1.0676
1.0500
1.0325
N
O
CH3
HO
2.98
3.99
1.09
1.05
1.91
1.85
2.06
2.97
4.31
6.00
3.12
f1 (ppm)

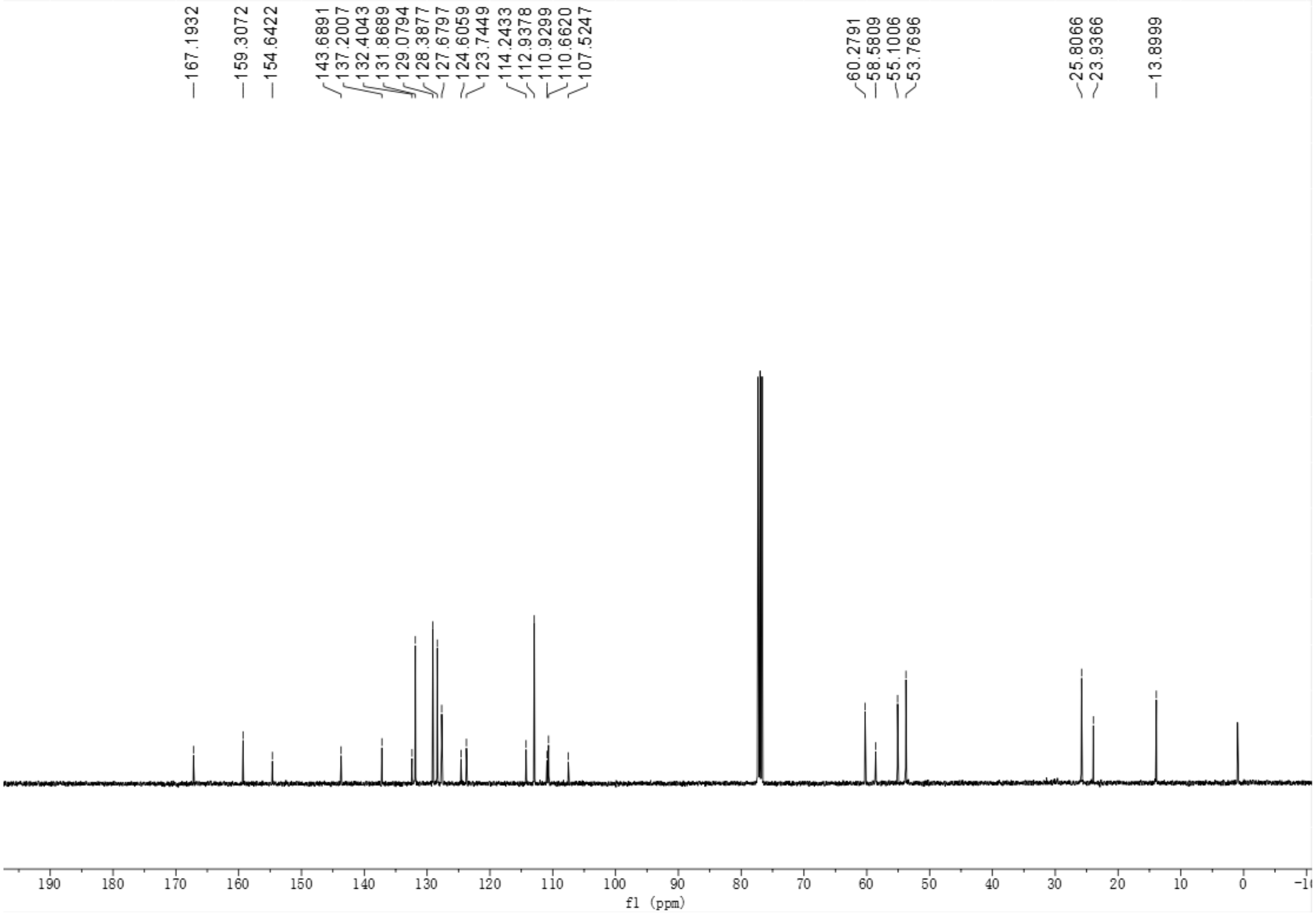
167.1932
159.3072
154.6422
143.6891
137.2007
132.4043
131.8689
129.0794
128.3877
127.6797
124.6059
123.7449
114.2433
112.9378
110.9299
110.6620
107.5247
60.2791
58.5809
55.1006
53.7696
25.8066
23.9366
13.8999
f1 (ppm)

18

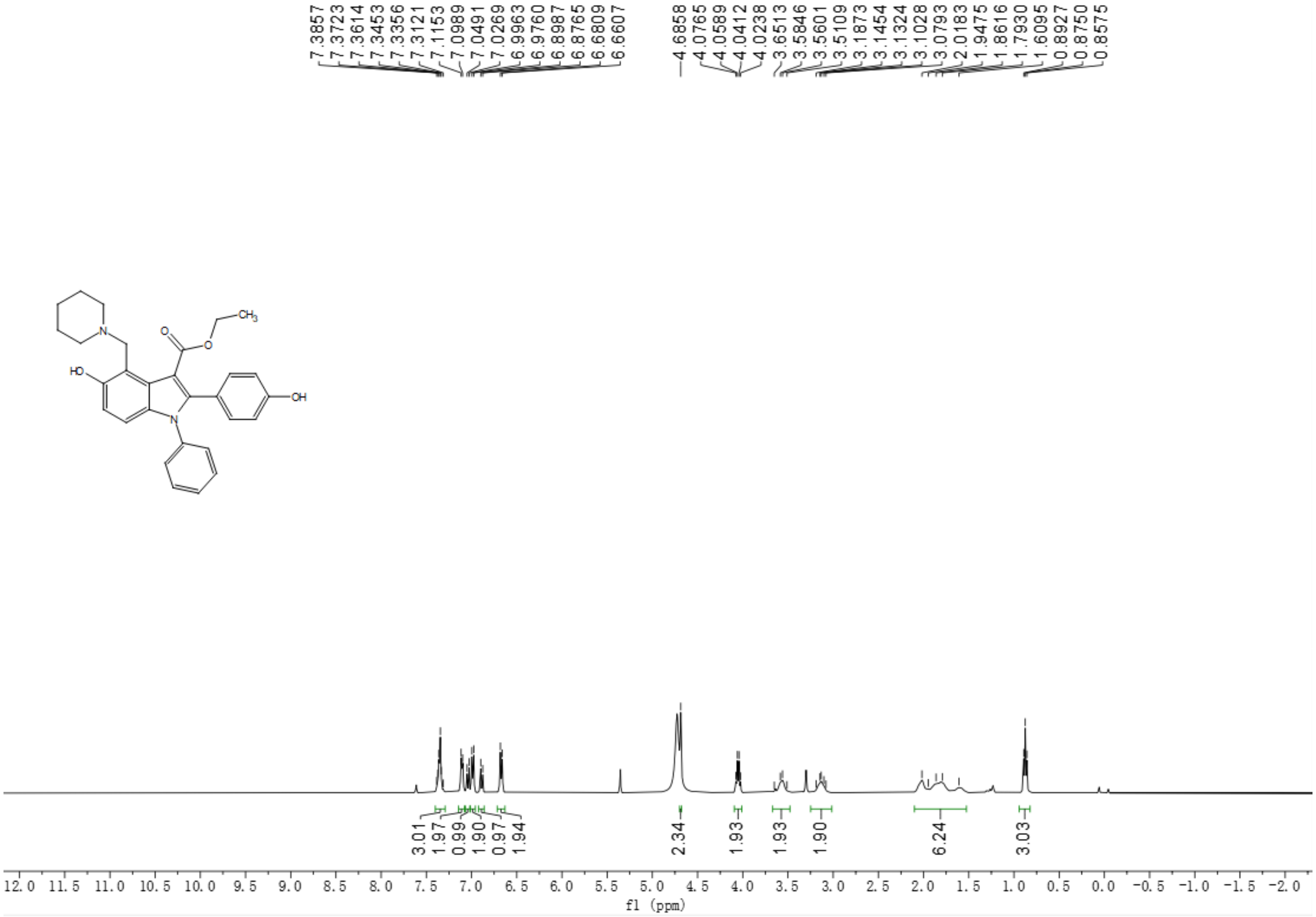
7.3857
7.3723
7.3614
7.3453
7.3356
7.3121
7.1153
7.0989
7.0491
7.0269
6.9963
6.9760
6.8987
6.8765
6.6809
6.6607
4.6858
4.0765
4.0589
4.0412
4.0238
3.6513
3.5846
3.5601
3.5109
3.1873
3.1454
3.1324
3.1028
3.0793
2.0183
1.9475
1.8616
1.7930
1.6095
0.8927
0.8750
0.8575
CH3
O
O
N
HO
N
OH
3.01
1.97
0.99
1.90
0.97
1.94
2.34
1.93
1.93
1.90
6.24
3.03
12.0 11.5 11.0 10.5 10.0 9.5 9.0 8.5 8.0 7.5 7.0 6.5 6.0 5.5 5.0 4.5 4.0 3.5 3.0 2.5 2.0 1.5 1.0 0.5 0.0 -0.5 -1.0 -1.5 -2.0
f1 (ppm)

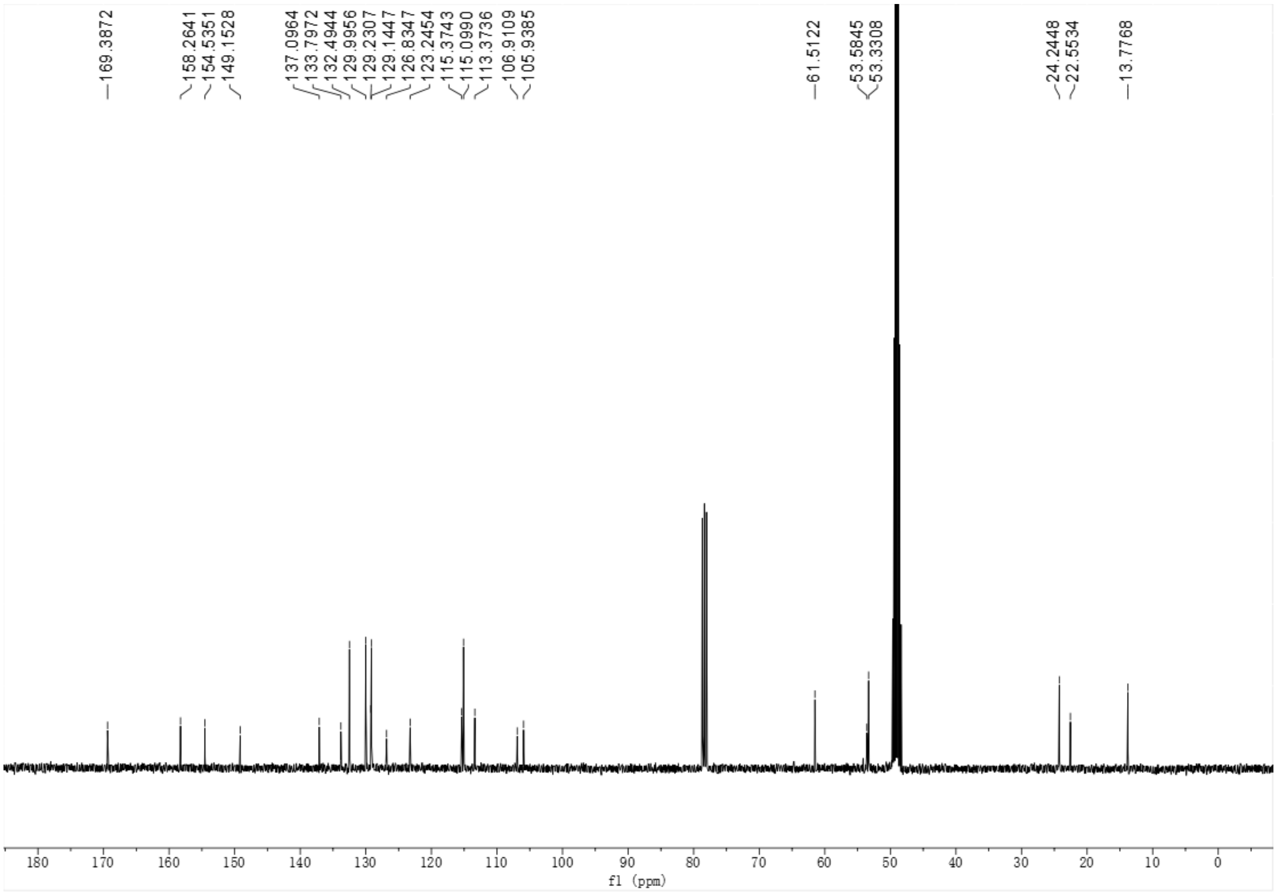
169.3872
158.2641
154.5351
149.1528
137.0964
133.7972
132.4944
129.9956
129.2307
129.1447
126.8347
123.2454
115.3743
115.0990
113.3736
106.9109
105.9385
61.5122
53.5845
53.3308
24.2448
22.5534
13.7768
180 170 160 150 140 130 120 110 100 90 80 70 60 50 40 30 20 10 0
f1 (ppm)

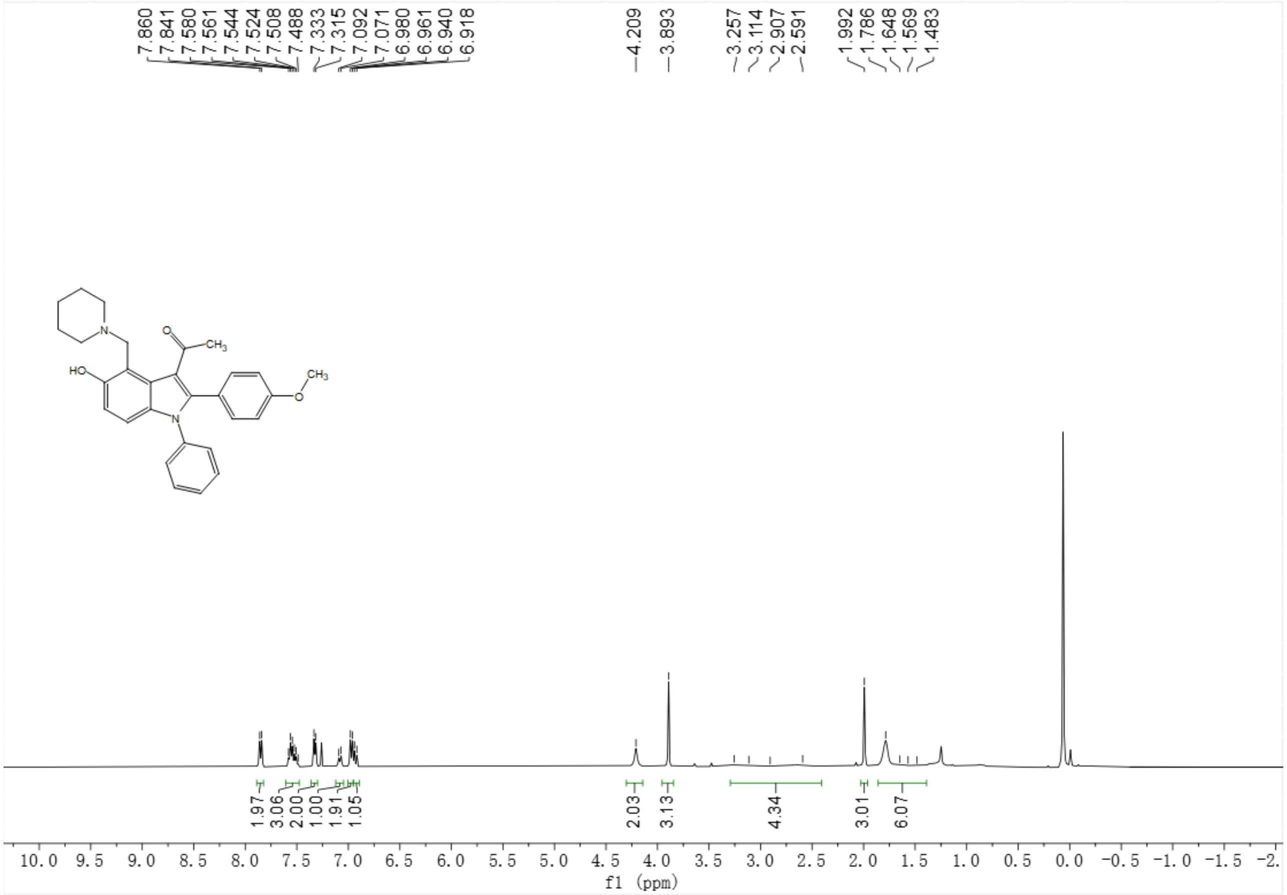

7.860
7.841
7.580
7.561
7.544
7.524
7.508
7.488
7.333
7.315
7.092
7.071
6.980
6.961
6.940
6.918
4.209
3.893
3.257
3.114
2.907
2.591
1.992
1.786
1.648
1.569
1.483
1.97
3.06
2.00
1.00
1.91
1.05
2.03
3.13
4.34
3.01
6.07
HO
N
O
CH3
10.0 9.5 9.0 8.5 8.0 7.5 7.0 6.5 6.0 5.5 5.0 4.5 4.0 3.5 3.0 2.5 2.0 1.5 1.0 0.5 0.0 −0.5 −1.0 −1.5
f1 (ppm)

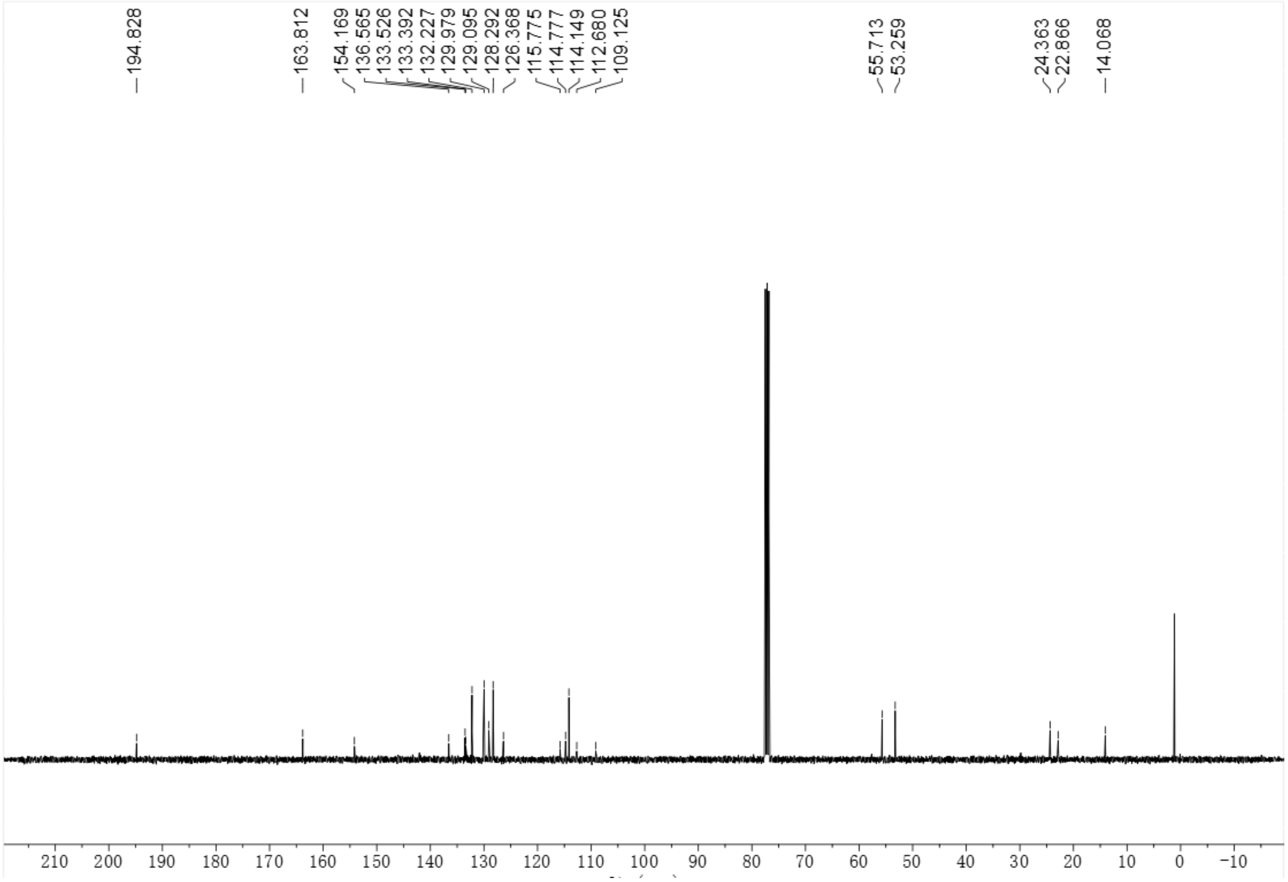

194.828
163.812
154.169
136.565
133.526
133.392
132.227
129.979
129.095
128.292
126.368
115.775
114.777
114.149
112.680
109.125
55.713
53.259
24.363
22.866
14.068
210 200 190 180 170 160 150 140 130 120 110 100 90 80 70 60 50 40 30 20 10 0 −10

20

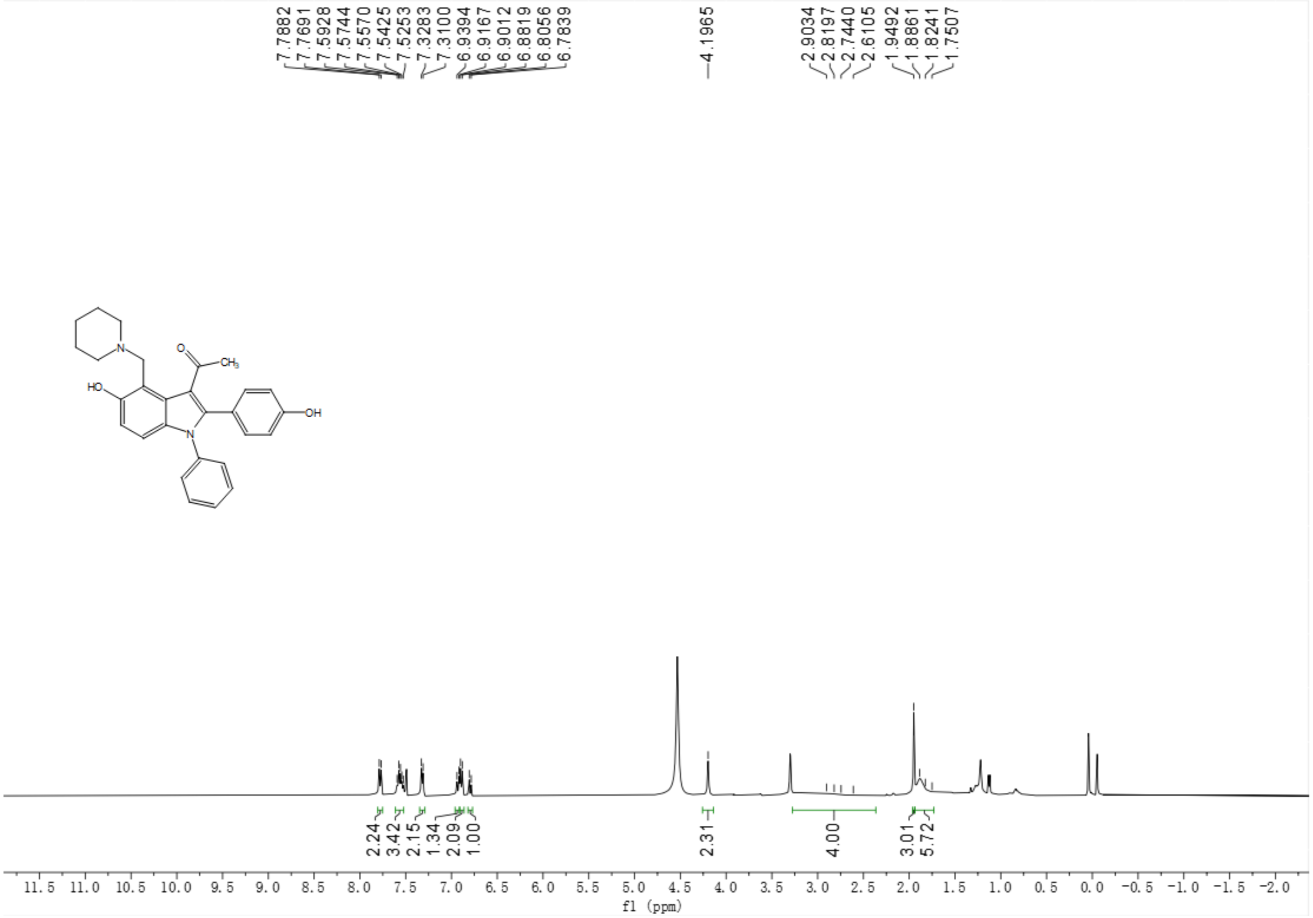
7.7882
7.7691
7.5928
7.5744
7.5570
7.5425
7.5253
7.3283
7.3100
6.9394
6.9167
6.9012
6.8819
6.8056
6.7839
4.1965
2.9034
2.8197
2.7440
2.6105
1.9492
1.8861
1.8241
1.7507
2.24
3.42
2.15
1.34
2.09
1.00
2.31
4.00
3.01
5.72
fl (ppm)

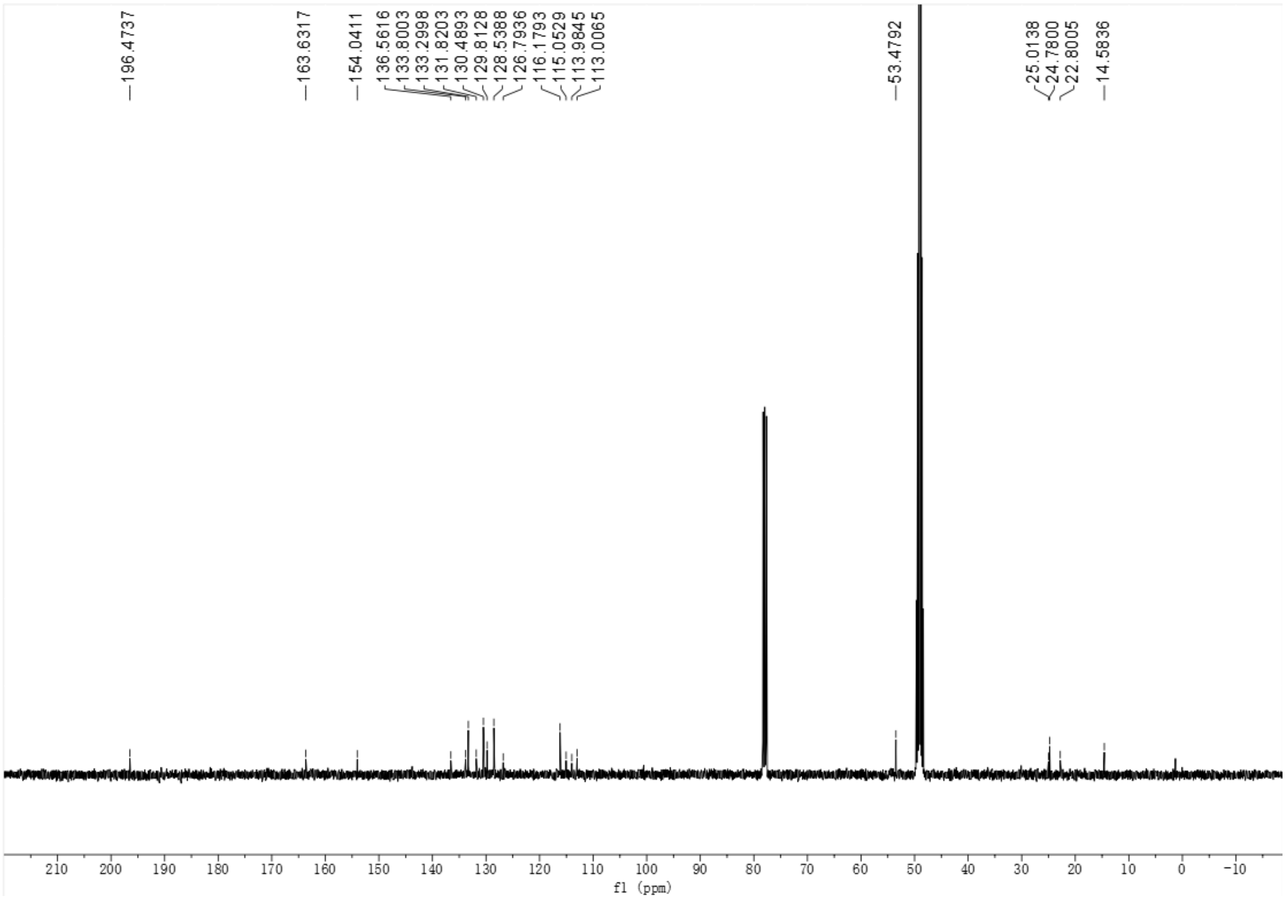
196.4737
163.6317
154.0411
136.5616
133.8003
133.2998
131.8203
130.4893
129.8128
128.5388
126.7936
116.1793
115.0529
113.9845
113.0065
53.4792
25.0138
24.7800
22.8005
14.5836
fl (ppm)

22

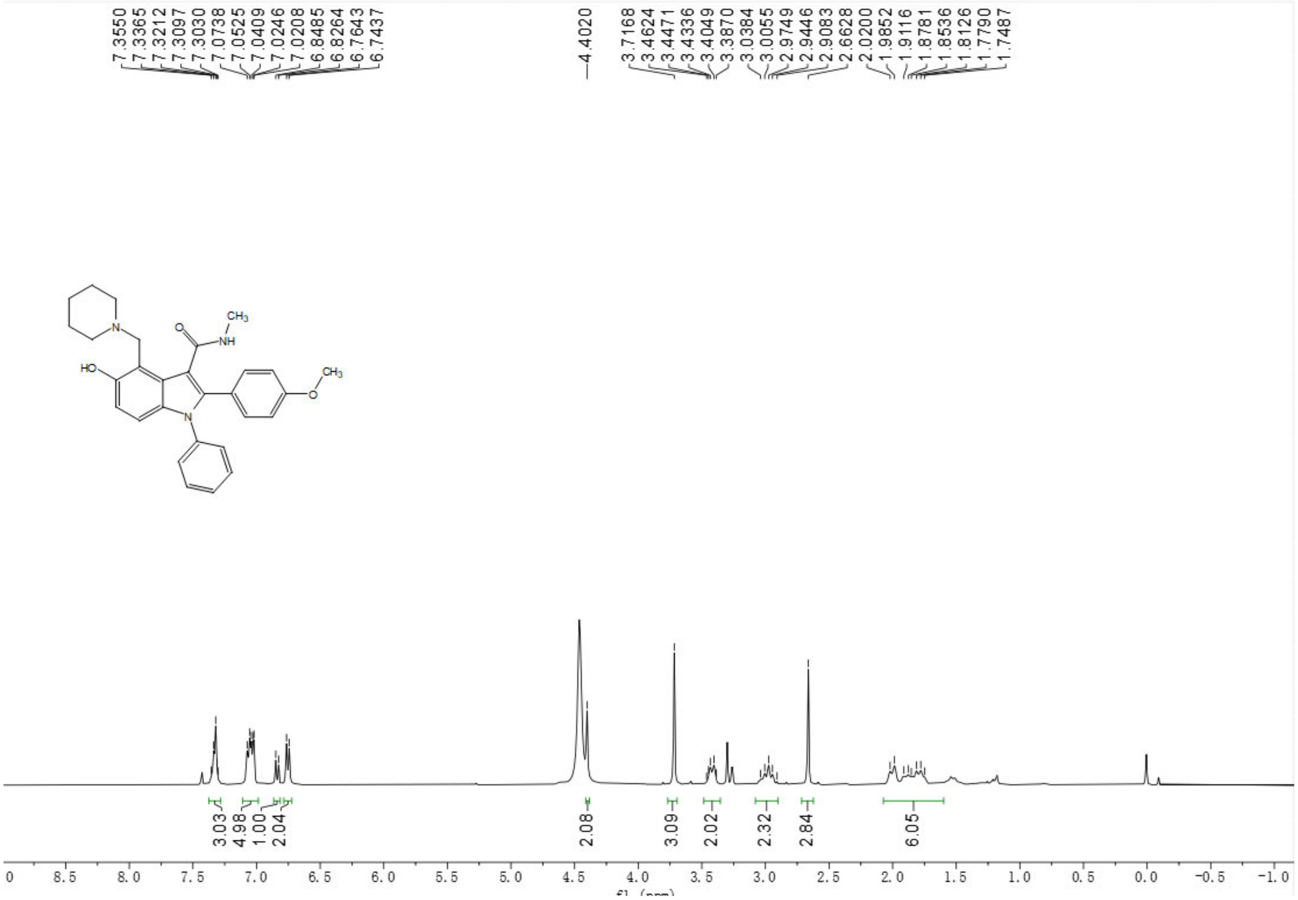
7.3550
7.3365
7.3212
7.3097
7.3030
7.0738
7.0525
7.0409
7.0246
7.0208
6.8485
6.8264
6.7643
6.7437
4.4020
3.7168
3.4624
3.4471
3.4336
3.4049
3.3870
3.0384
3.0055
2.9749
2.9446
2.9083
2.6628
2.0200
1.9852
1.9116
1.8781
1.8536
1.8126
1.7790
1.7487
HO
N
O
NH
CH3
O
CH3
N
3.03
4.98
1.00
2.04
2.08
3.09
2.02
2.32
2.84
6.05
8.5
8.0
7.5
7.0
6.5
6.0
5.5
5.0
4.5
4.0
3.5
3.0
2.5
2.0
1.5
1.0
0.5
0.0
-0.5
-1.0

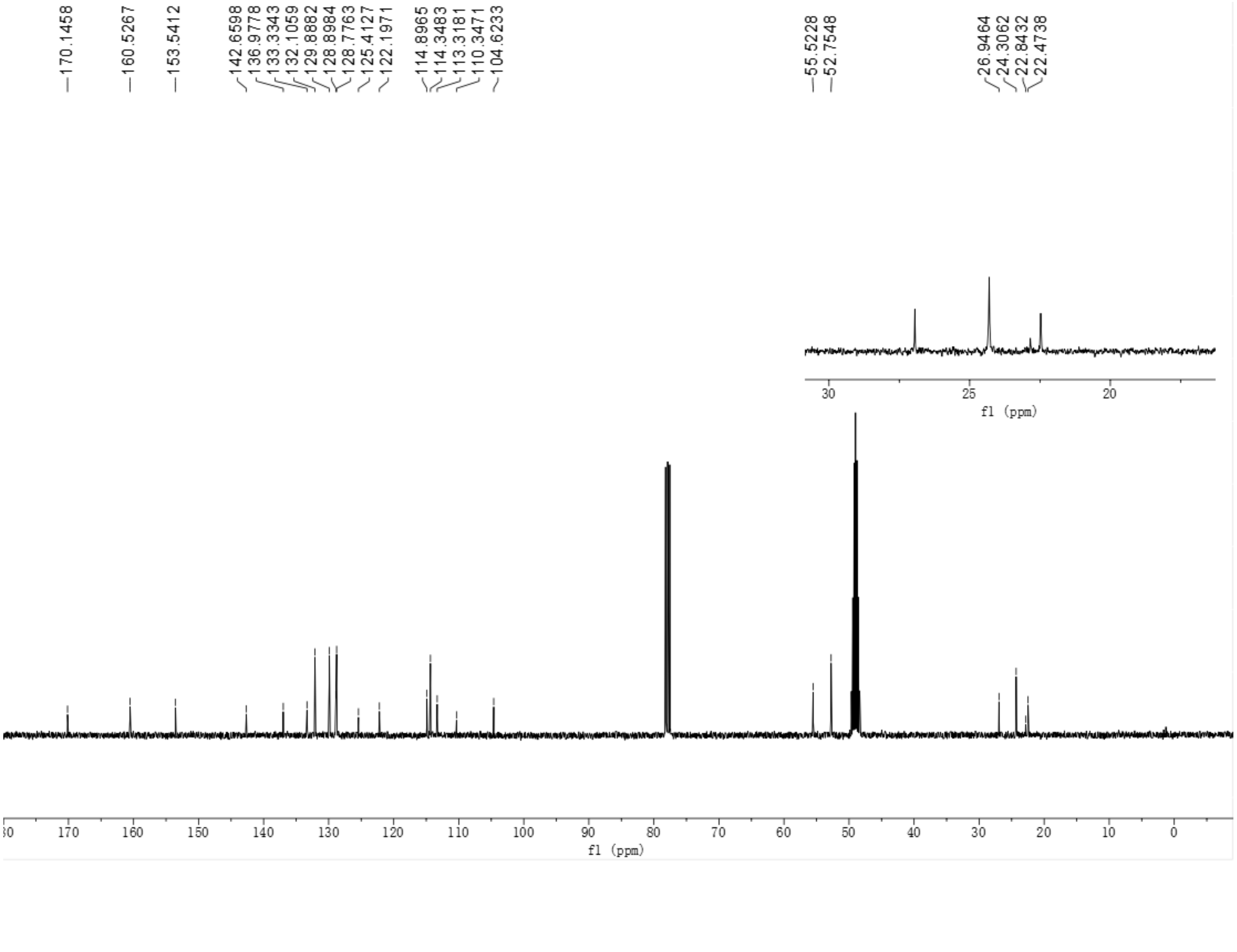
170.1458
160.5267
153.5412
142.6598
136.9778
133.3343
132.1059
129.8882
128.8984
128.7763
125.4127
122.1971
114.8965
114.3483
113.3181
110.3471
104.6233
55.5228
52.7548
26.9464
24.3062
22.8432
22.4738
30
25
20
f1 (ppm)
170
160
150
140
130
120
110
100
90
80
70
60
50
40
30
20
10
0
f1 (ppm)

23

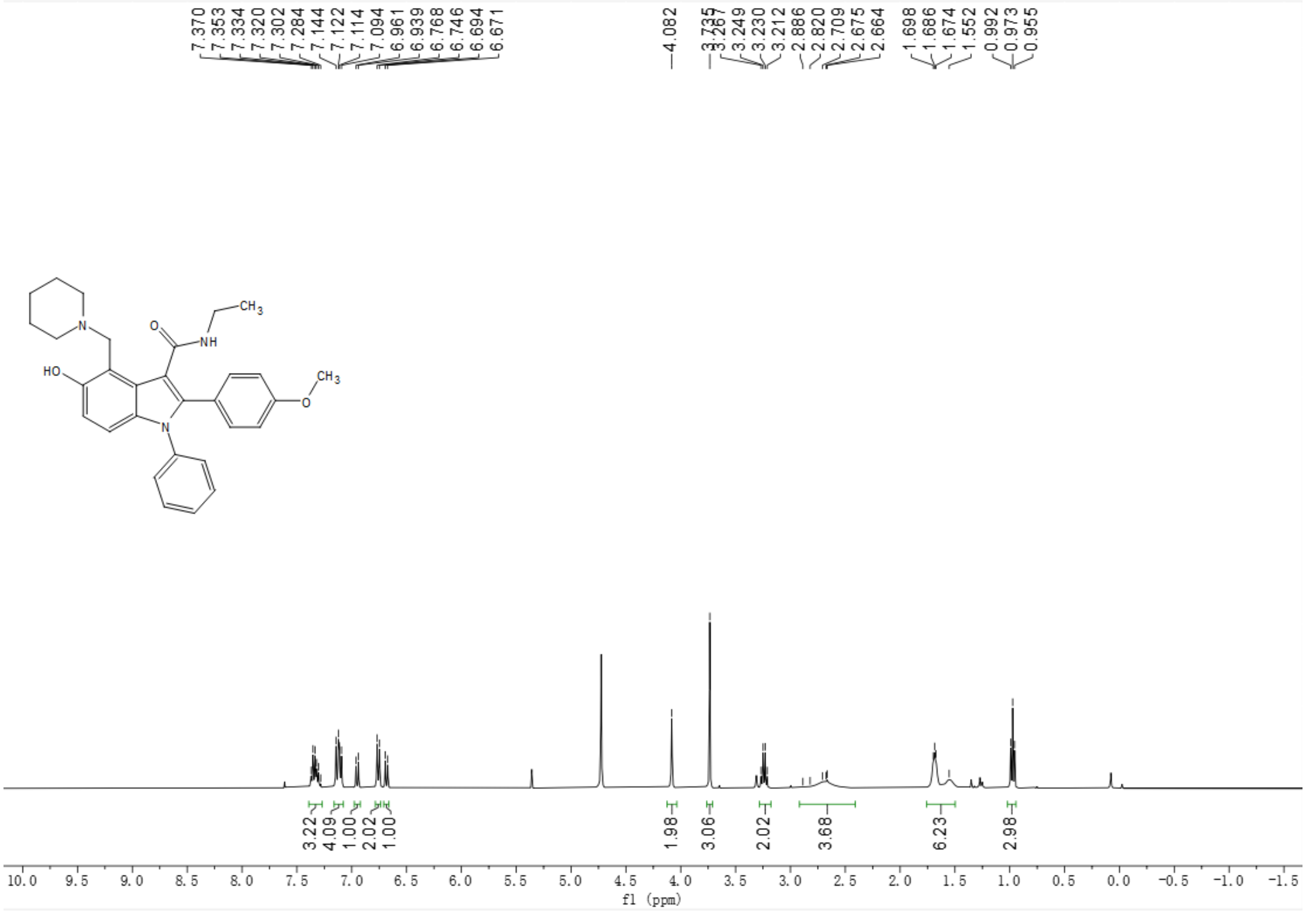
7.370
7.353
7.334
7.320
7.302
7.284
7.144
7.122
7.114
7.094
6.961
6.939
6.768
6.746
6.694
6.671
4.082
[illegible]
3.249
3.230
3.212
2.886
2.820
2.709
2.675
2.664
1.698
1.686
1.674
1.552
0.992
0.973
0.955
CH3
O
NH
N
HO
CH3
O
N
3.22
4.09
1.00
2.02
1.00
1.98
3.06
2.02
3.68
6.23
2.98
f1 (ppm)

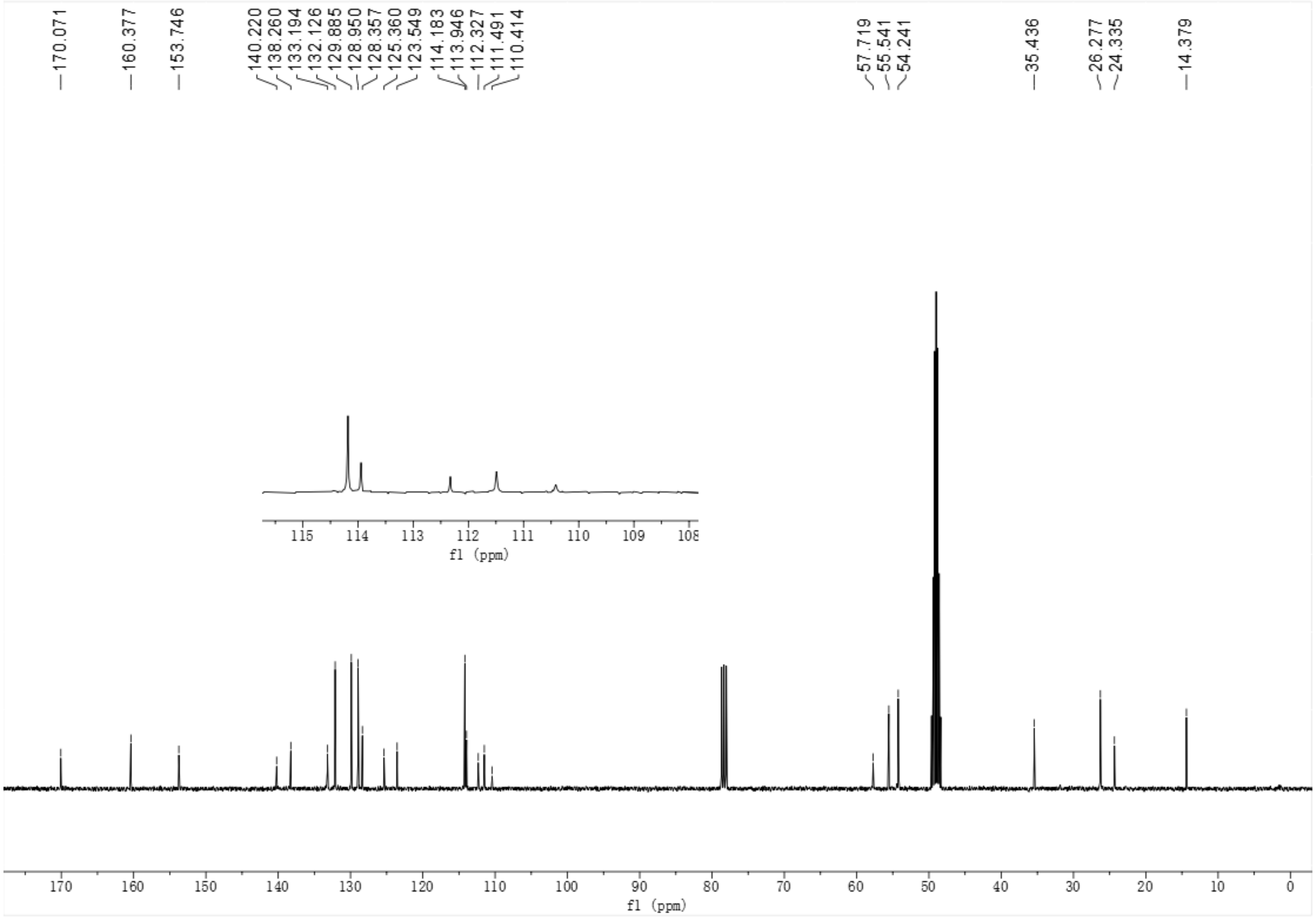
170.071
160.377
153.746
140.220
138.260
133.194
132.126
129.885
128.950
128.357
125.360
123.549
114.183
113.946
112.327
111.491
110.414
57.719
55.541
54.241
35.436
26.277
24.335
14.379
f1 (ppm)
f1 (ppm)

24

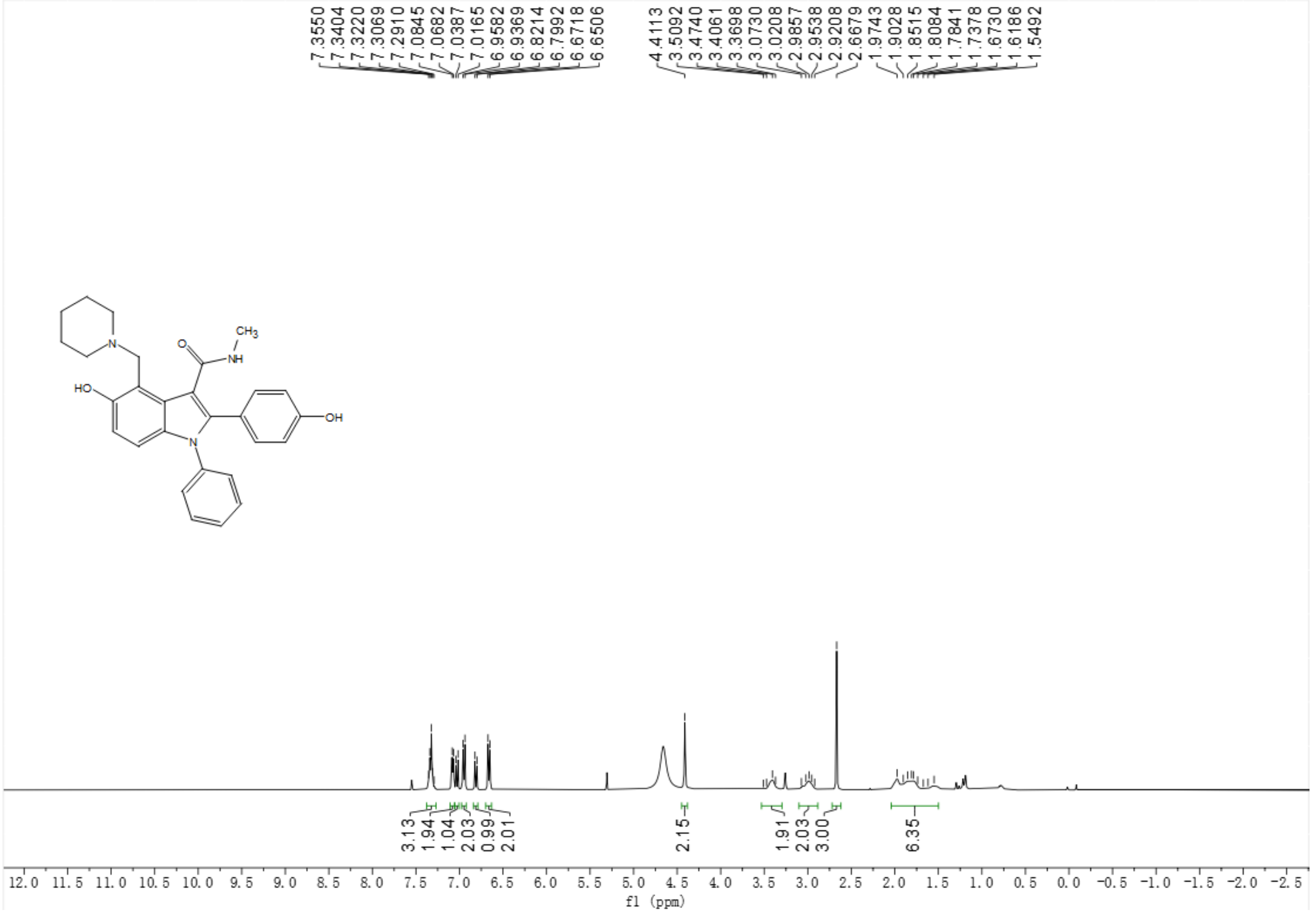
7.3550
7.3404
7.3220
7.3069
7.2910
7.0845
7.0682
7.0387
7.0165
6.9582
6.9369
6.8214
6.7992
6.6718
6.6506
4.4113
3.5092
3.4740
3.4061
3.3698
3.0730
3.0208
2.9857
2.9538
2.9208
2.6679
1.9743
1.9028
1.8515
1.8084
1.7841
1.7378
1.6730
1.6186
1.5492
CH3
O
NH
N
HO
OH
N
3.13
1.94
1.04
2.03
0.99
2.01
2.15
1.91
2.03
3.00
6.35
f1 (ppm)

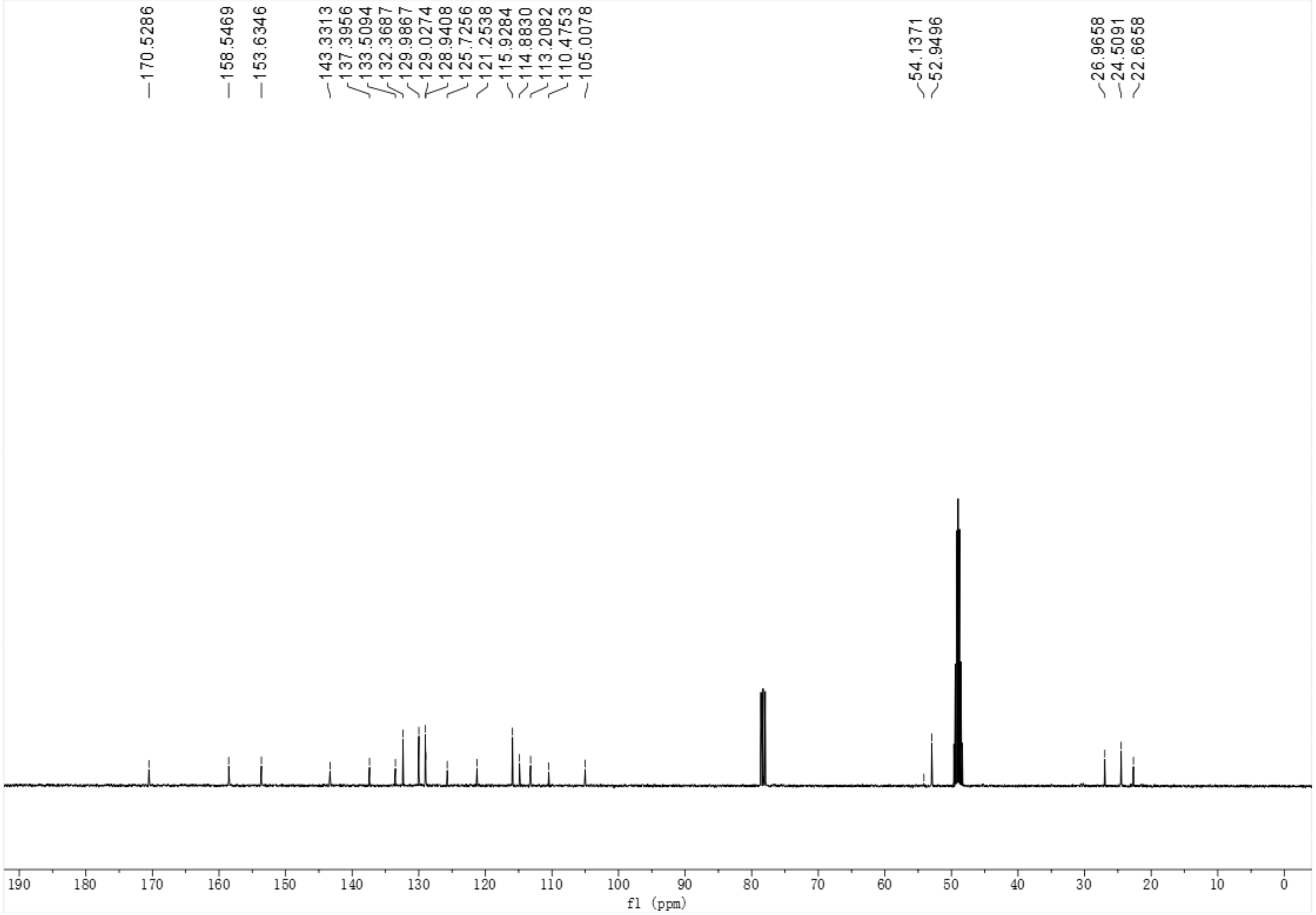
170.5286
158.5469
153.6346
143.3313
137.3956
133.5094
132.3687
129.9867
129.0274
128.9408
125.7256
121.2538
115.9284
114.8830
113.2082
110.4753
105.0078
54.1371
52.9496
26.9658
24.5091
22.6658
f1 (ppm)

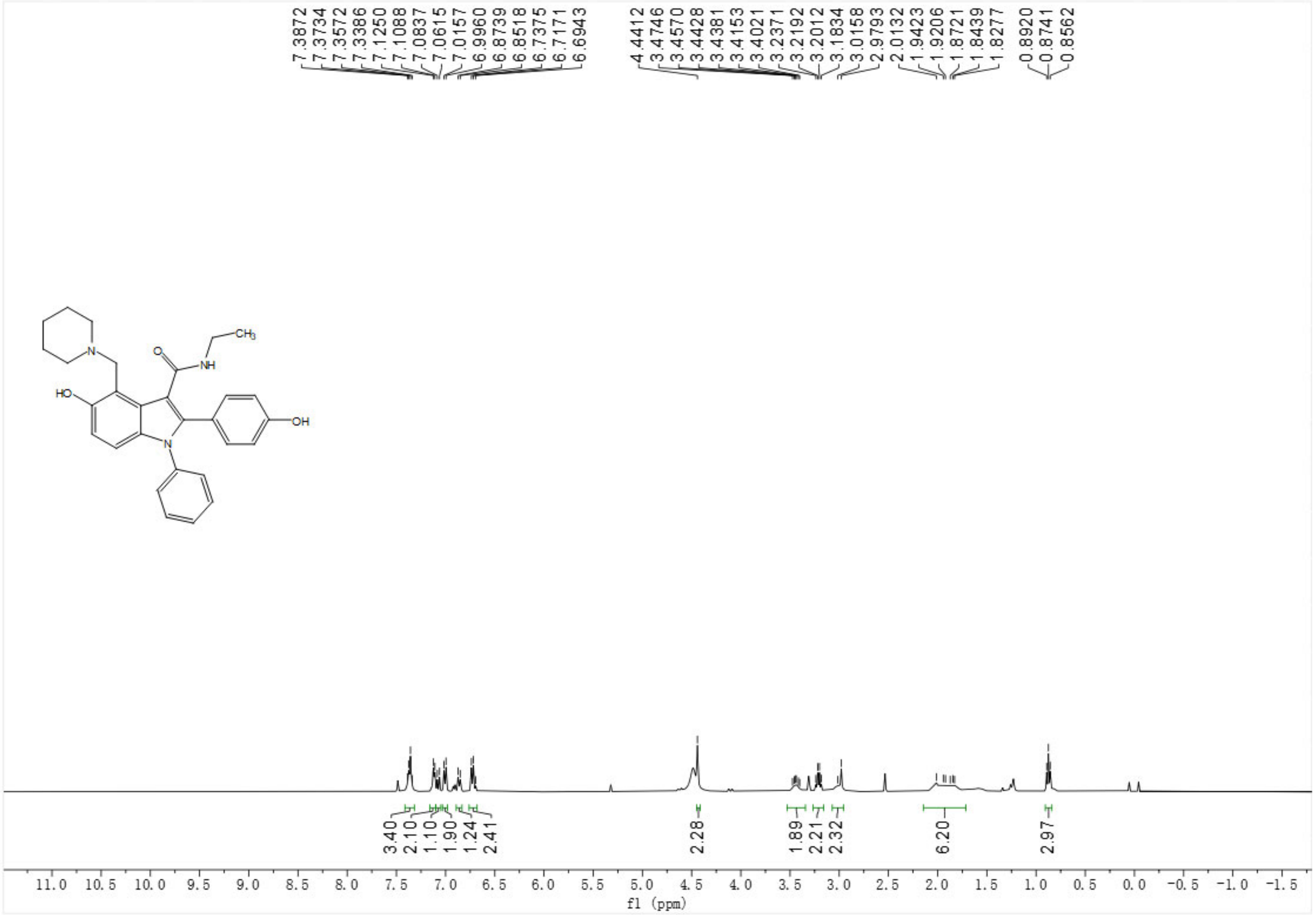
7.3872
7.3734
7.3572
7.3386
7.1250
7.1088
7.0837
7.0615
7.0157
6.9960
6.8739
6.8518
6.7375
6.7171
6.6943
4.4412
3.4746
3.4570
3.4428
3.4381
3.4153
3.4021
3.2371
3.2192
3.2012
3.1834
3.0158
2.9793
2.0132
1.9423
1.9206
1.8721
1.8439
1.8277
0.8920
0.8741
0.8562
CH3
O
NH
N
HO
OH
N
3.40
2.10
1.10
1.90
1.24
2.41
2.28
1.89
2.21
2.32
6.20
2.97
fl (ppm)

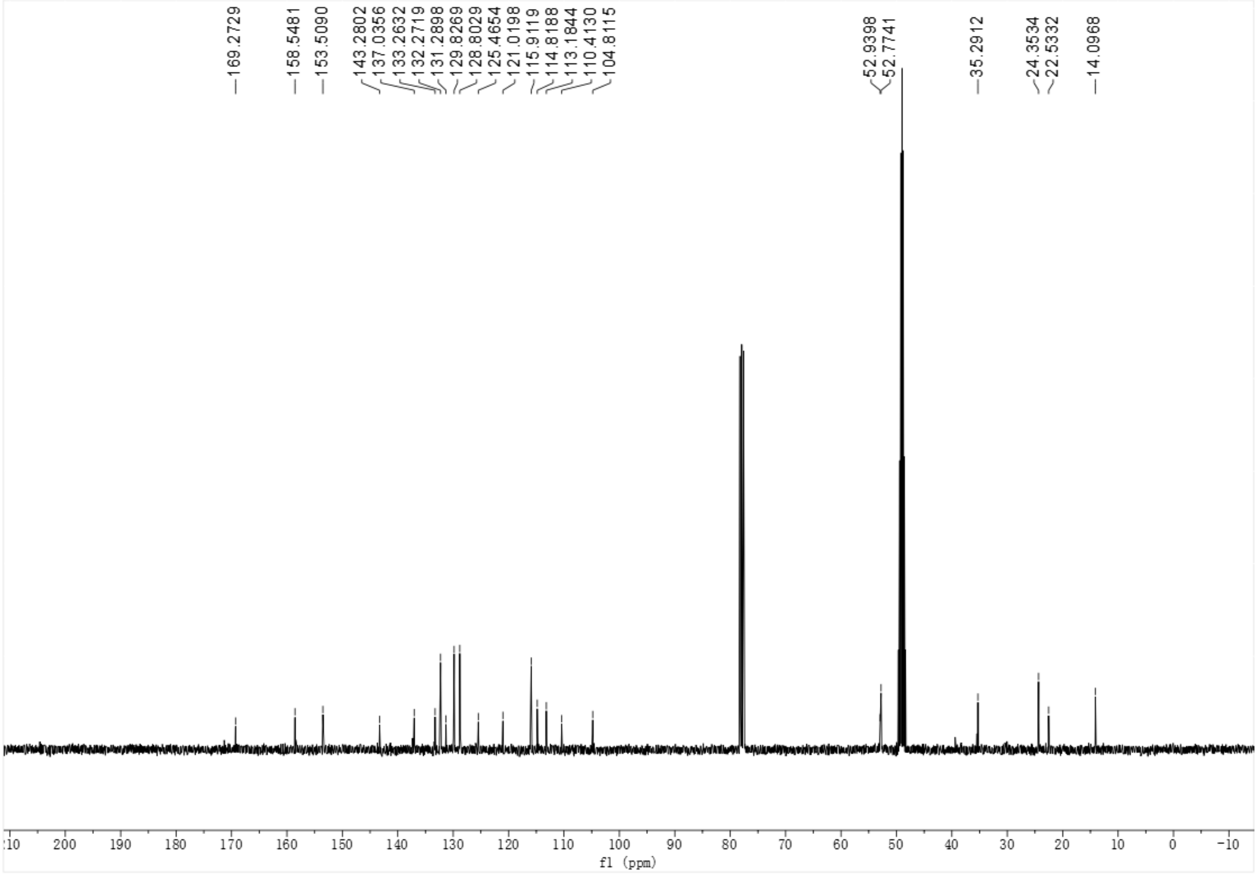
169.2729
158.5481
153.5090
143.2802
137.0356
133.2632
132.2719
131.2898
129.8269
128.8029
125.4654
121.0198
115.9119
114.8188
113.1844
110.4130
104.8115
52.9398
52.7741
35.2912
24.3534
22.5332
14.0968
fl (ppm)

28

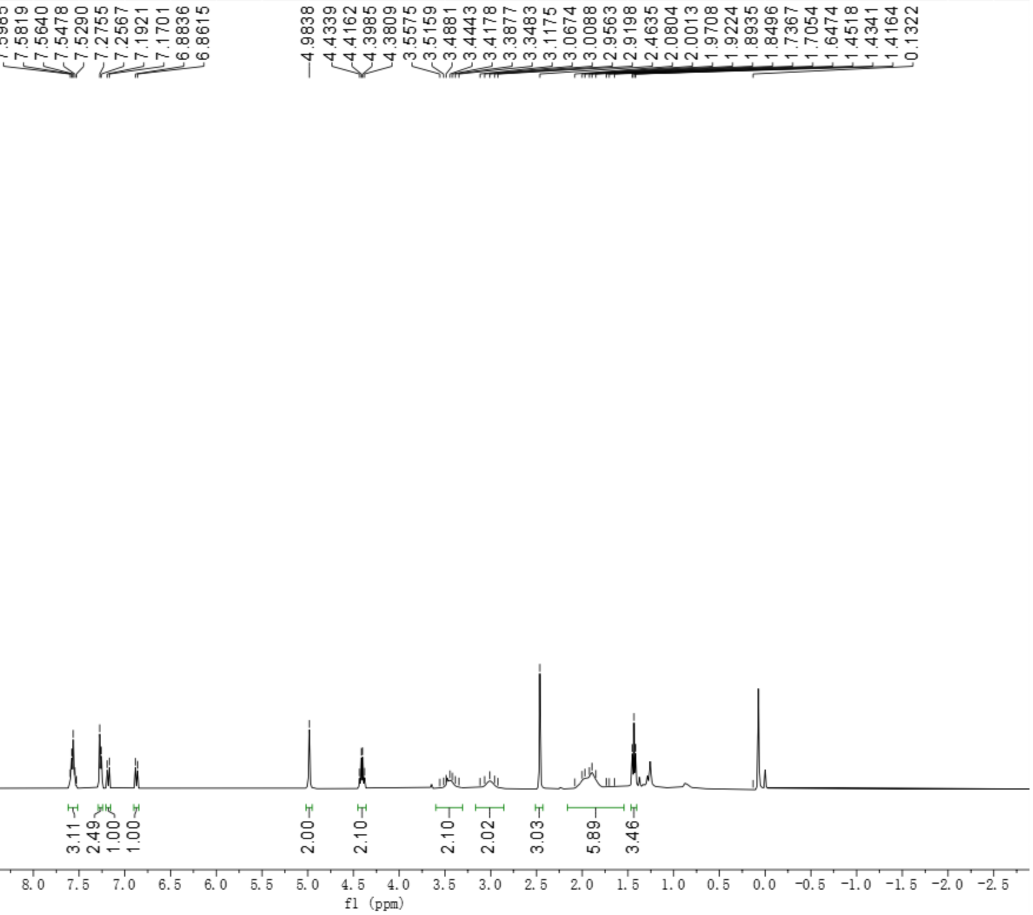

7.5985
7.5819
7.5640
7.5478
7.5290
7.2755
7.2567
7.1921
7.1701
6.8836
6.8615
4.9838
4.4339
4.4162
4.3985
4.3809
3.5575
3.5159
3.4881
3.4443
3.4178
3.3877
3.3483
3.1175
3.0674
3.0088
2.9563
2.9198
2.4635
2.0804
2.0013
1.9708
1.9224
1.8935
1.8496
1.7367
1.7054
1.6474
1.4518
1.4341
1.4164
0.1322
3.11
2.49
1.00
1.00
2.00
2.10
2.10
2.02
3.03
5.89
3.46
f1 (ppm)

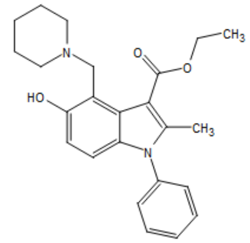

HO
N
O
O
CH3
CH3
N

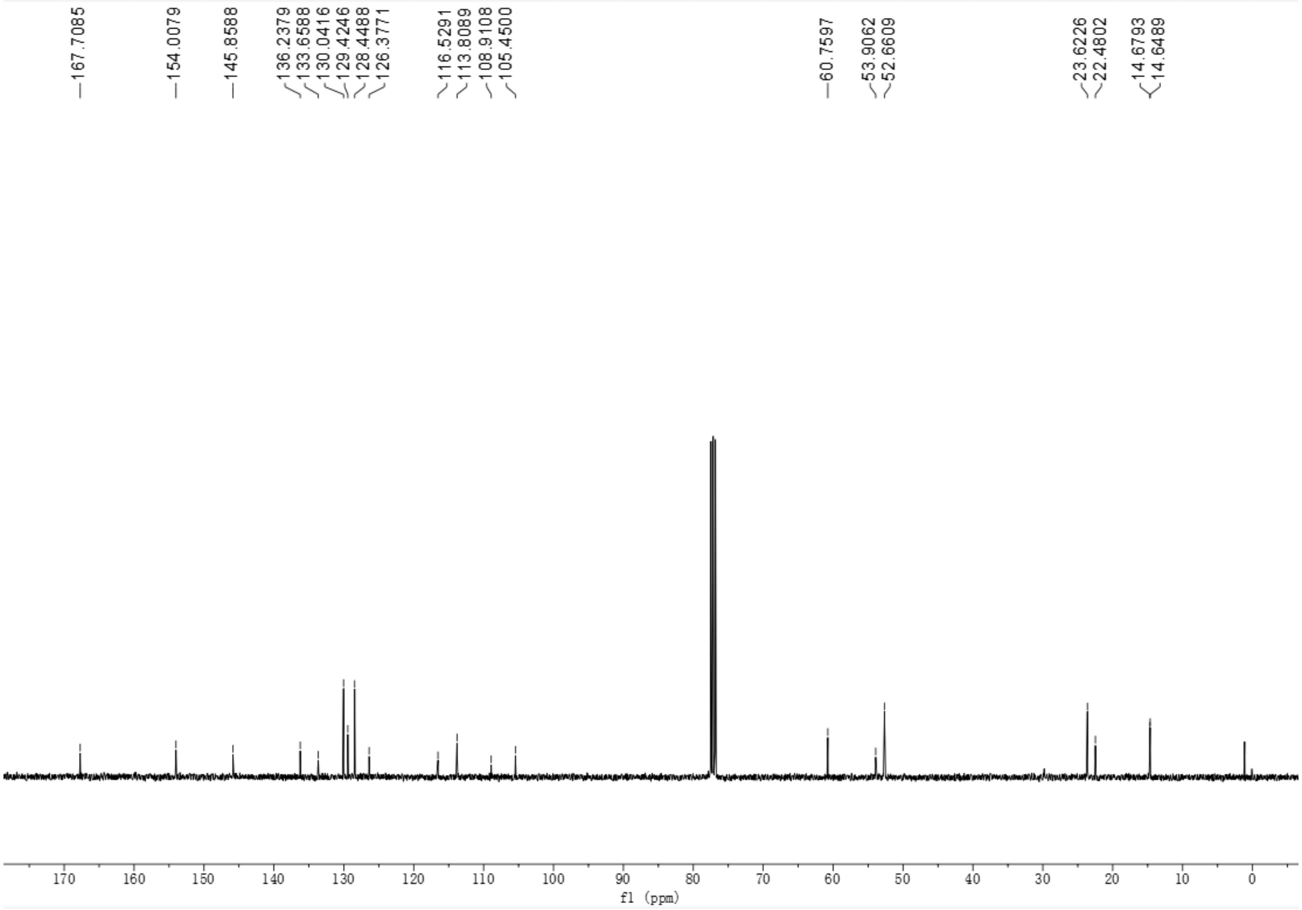

167.7085
154.0079
145.8588
136.2379
133.6588
130.0416
129.4246
128.4488
126.3771
116.5291
113.8089
108.9108
105.4500
60.7597
53.9062
52.6609
23.6226
22.4802
14.6793
14.6489
f1 (ppm)

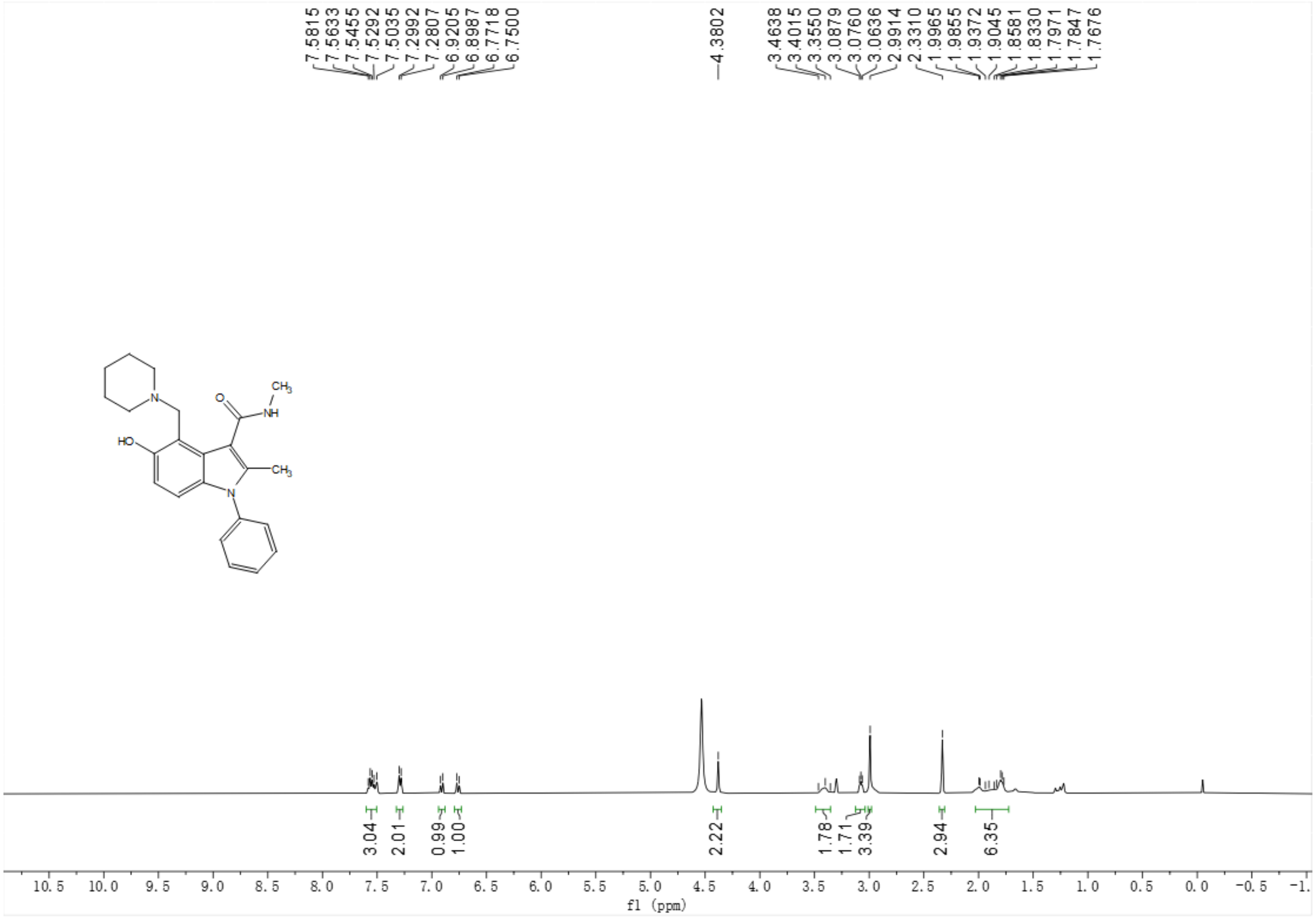

7.5815
7.5633
7.5455
7.5292
7.5035
7.2992
7.2807
6.9205
6.8987
6.7718
6.7500
4.3802
3.4638
3.4015
3.3550
3.0879
3.0760
3.0636
2.9914
2.3310
1.9965
1.9855
1.9372
1.9045
1.8581
1.8330
1.7971
1.7847
1.7676
3.04
2.01
0.99
1.00
2.22
1.78
1.71
3.39
2.94
6.35
f1 (ppm)

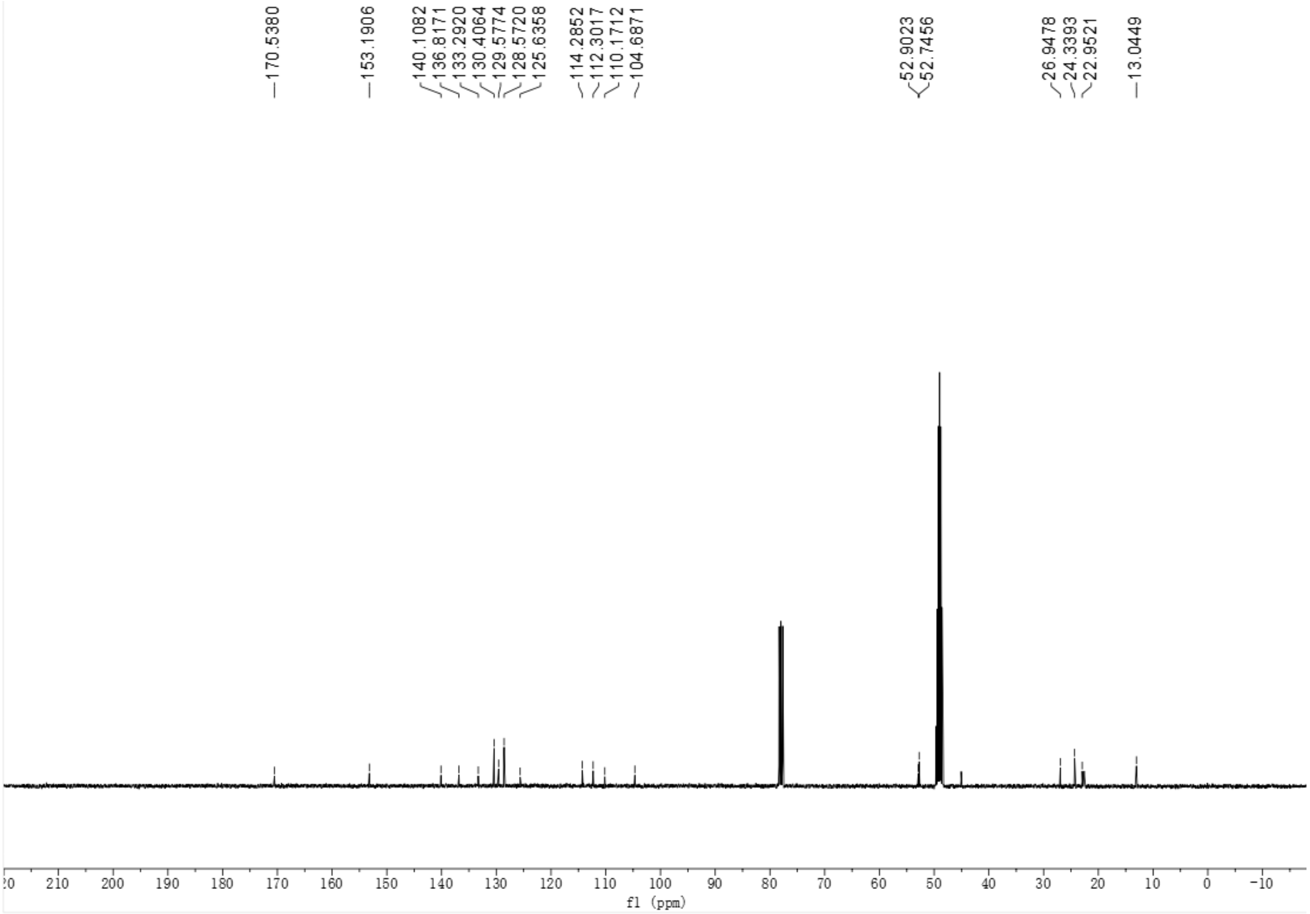

170.5380
153.1906
140.1082
136.8171
133.2920
130.4064
129.5774
128.5720
125.6358
114.2852
112.3017
110.1712
104.6871
52.9023
52.7456
26.9478
24.3393
22.9521
13.0449
f1 (ppm)

30

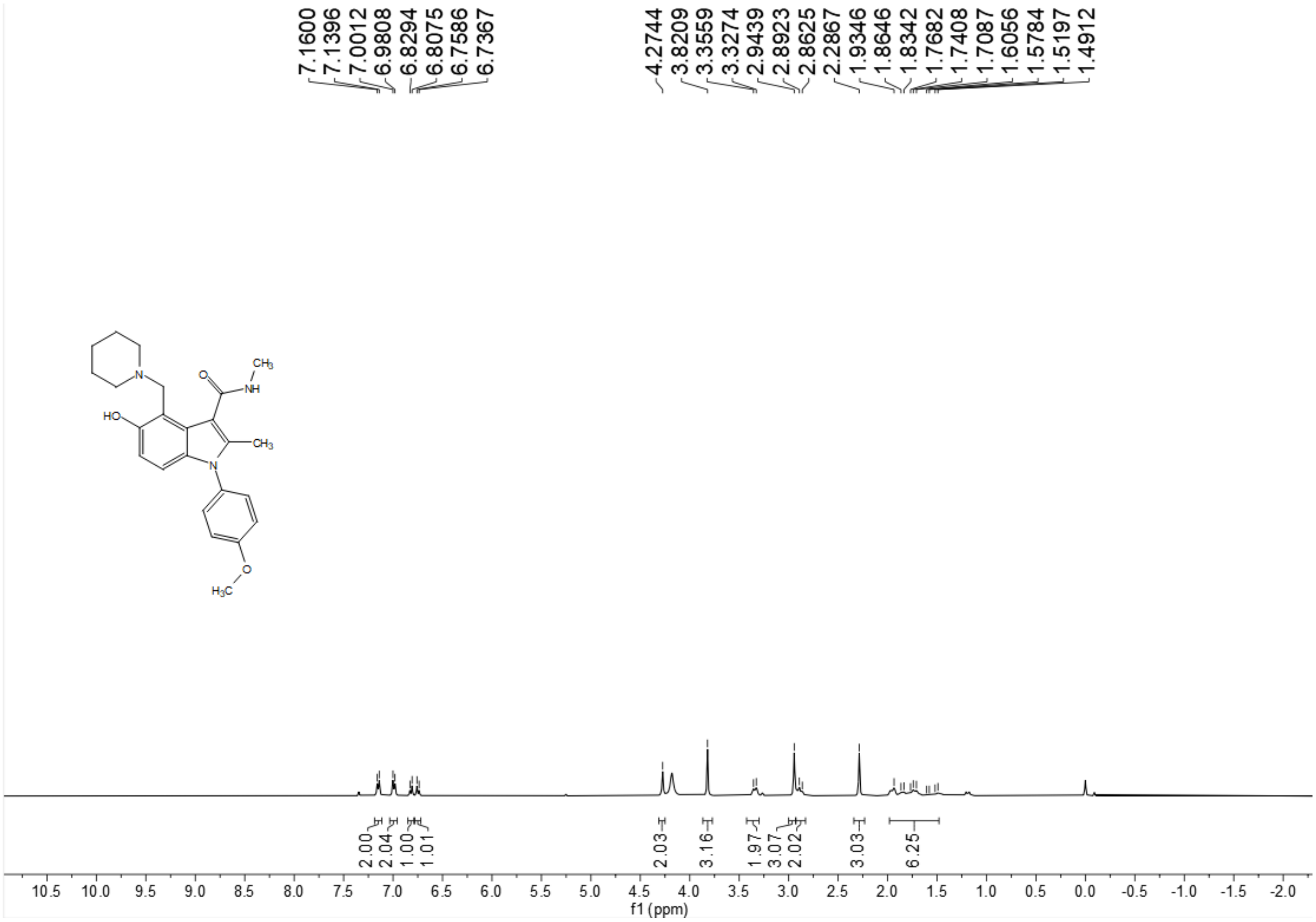
7.1600
7.1396
7.0012
6.9808
6.8294
6.8075
6.7586
6.7367
4.2744
3.8209
3.3559
3.3274
2.9439
2.8923
2.8625
2.2867
1.9346
1.8646
1.8342
1.7682
1.7408
1.7087
1.6056
1.5784
1.5197
1.4912
CH3
O
NH
N
HO
CH3
N
O
H3C
2.00
2.04
1.00
1.01
2.03
3.16
1.97
3.07
2.02
3.03
6.25
10.5 10.0 9.5 9.0 8.5 8.0 7.5 7.0 6.5 6.0 5.5 5.0 4.5 4.0 3.5 3.0 2.5 2.0 1.5 1.0 0.5 0.0 -0.5 -1.0 -1.5 -2.0
f1 (ppm)

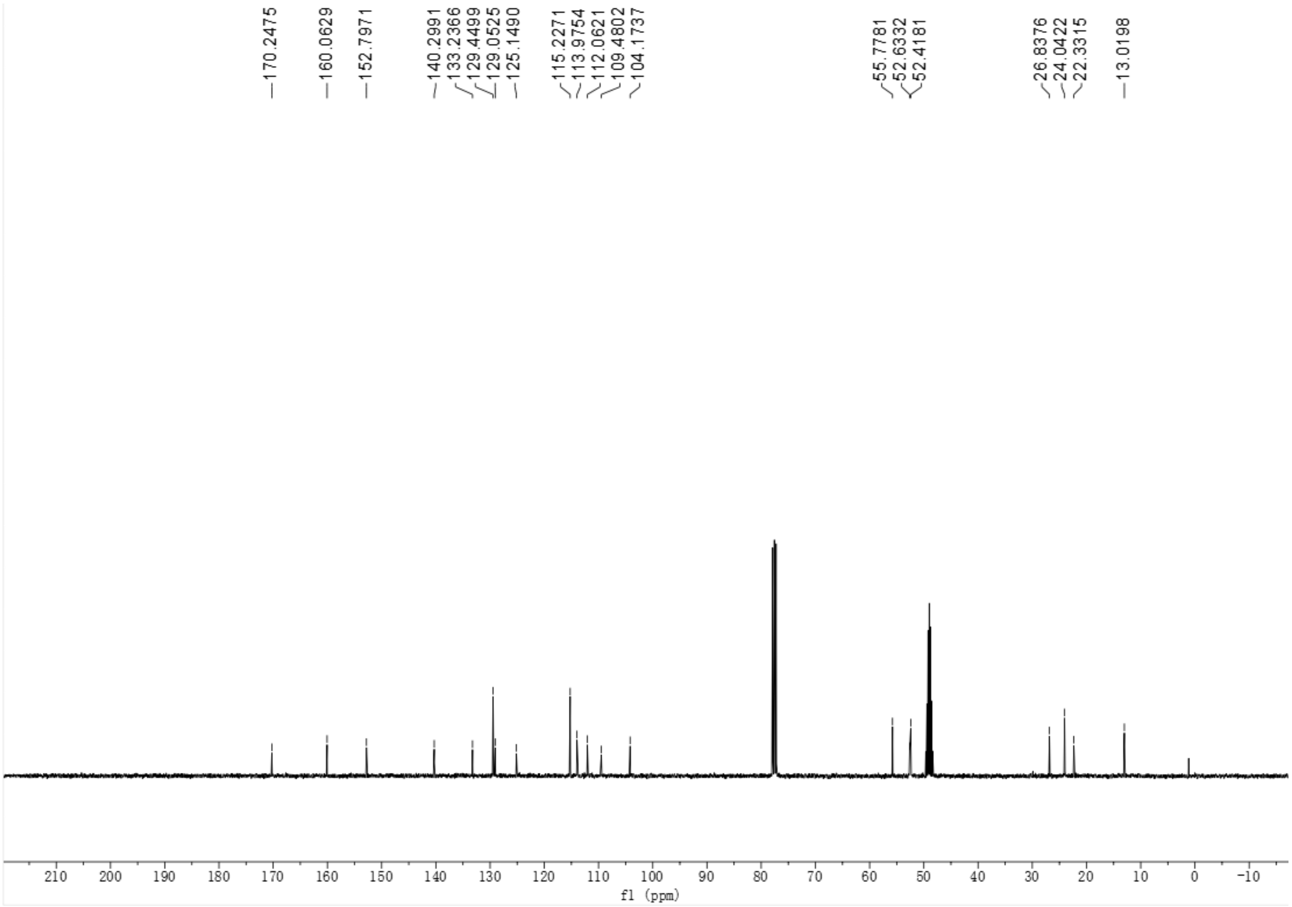
170.2475
160.0629
152.7971
140.2991
133.2366
129.4499
129.0525
125.1490
115.2271
113.9754
112.0621
109.4802
104.1737
55.7781
52.6332
52.4181
26.8376
24.0422
22.3315
13.0198
210 200 190 180 170 160 150 140 130 120 110 100 90 80 70 60 50 40 30 20 10 0 -10
f1 (ppm)

31

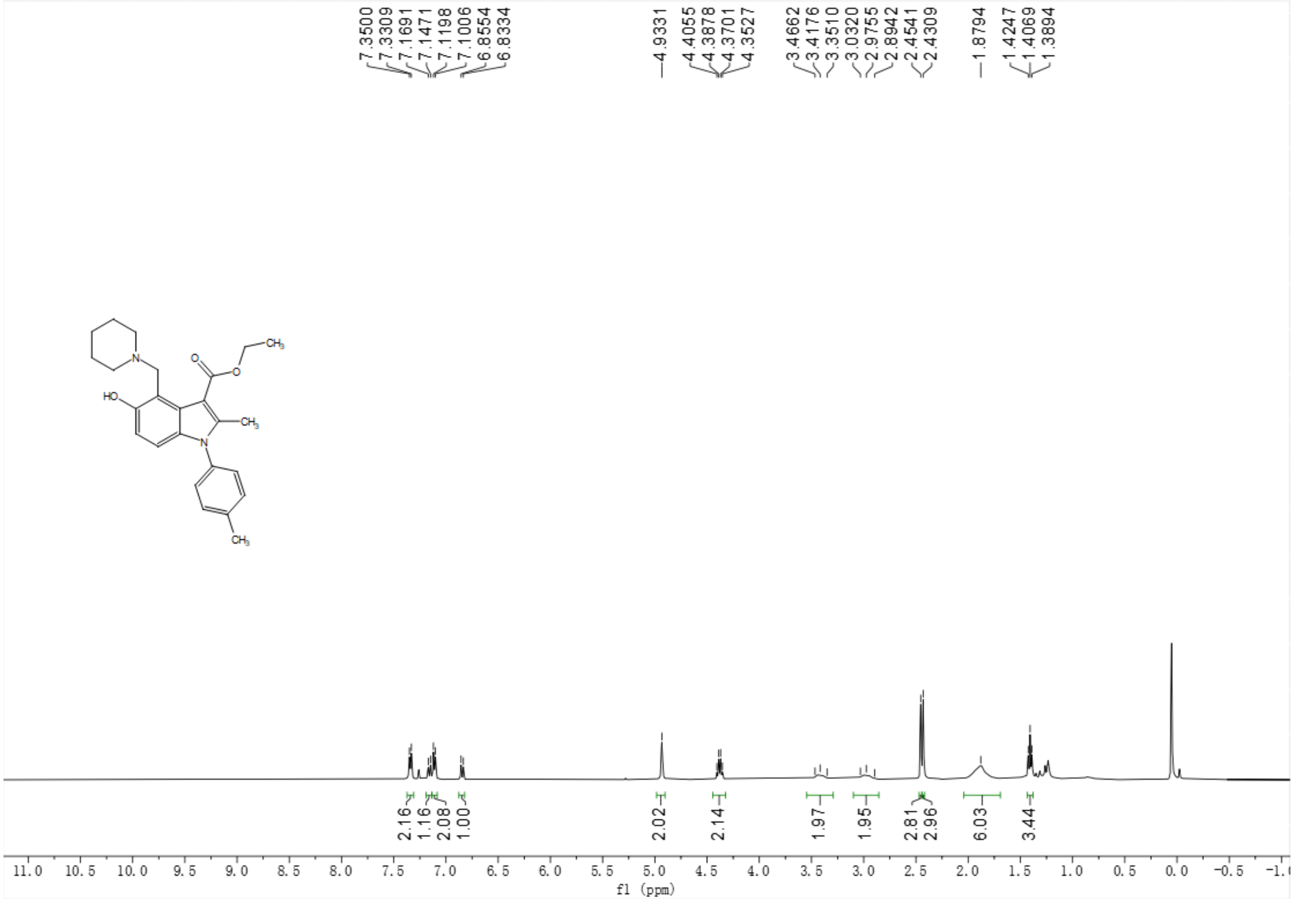
7.3500
7.3309
7.1691
7.1471
7.1198
7.1006
6.8554
6.8334
4.9331
4.4055
4.3878
4.3701
4.3527
3.4662
3.4176
3.3510
3.0320
2.9755
2.8942
2.4541
2.4309
1.8794
1.4247
1.4069
1.3894
HO
O
O
N
N
CH3
CH3
CH3
2.16
1.16
2.08
1.00
2.02
2.14
1.97
1.95
2.81
2.96
6.03
3.44
11.0 10.5 10.0 9.5 9.0 8.5 8.0 7.5 7.0 6.5 6.0 5.5 5.0 4.5 4.0 3.5 3.0 2.5 2.0 1.5 1.0 0.5 0.0 -0.5
f1 (ppm)

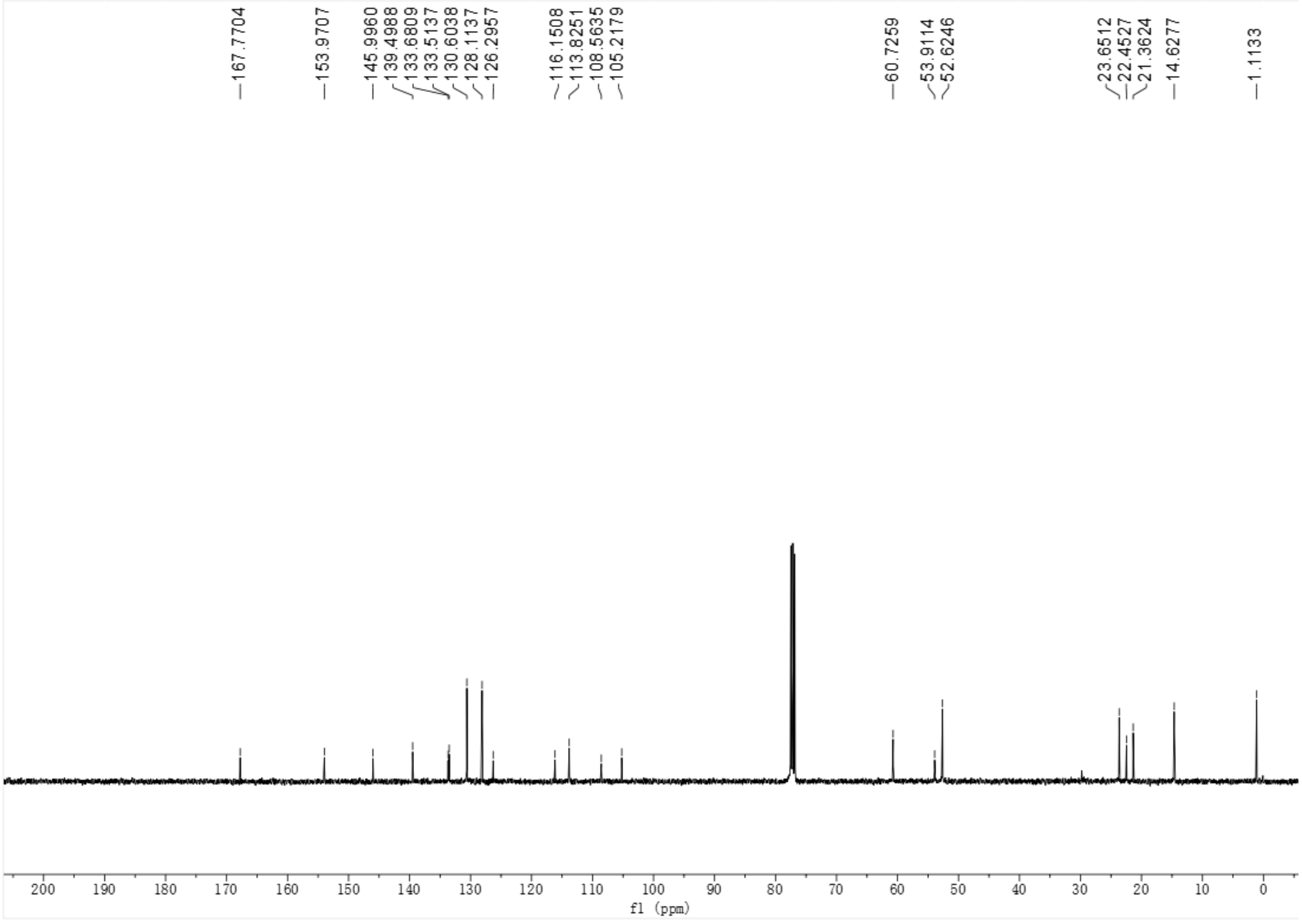
167.7704
153.9707
145.9960
139.4988
133.6809
133.5137
130.6038
128.1137
126.2957
116.1508
113.8251
108.5635
105.2179
60.7259
53.9114
52.6246
23.6512
22.4527
21.3624
14.6277
1.1133
200 190 180 170 160 150 140 130 120 110 100 90 80 70 60 50 40 30 20 10 0
f1 (ppm)

32

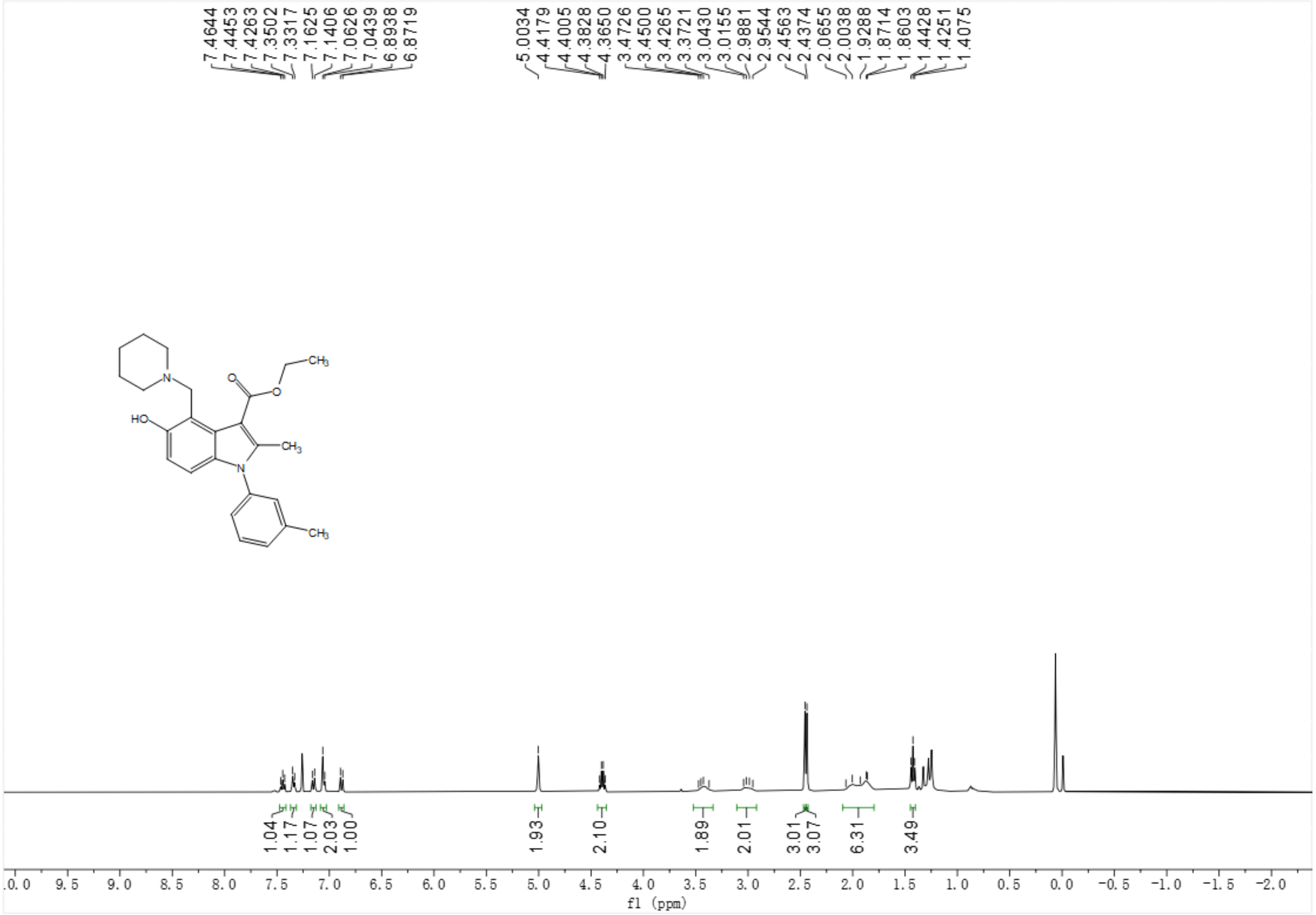

7.4644
7.4453
7.4263
7.3502
7.3317
7.1625
7.1406
7.0626
7.0439
6.8938
6.8719
5.0034
4.4179
4.4005
4.3828
4.3650
3.4726
3.4500
3.4265
3.3721
3.0430
3.0155
2.9881
2.9544
2.4563
2.4374
2.0655
2.0038
1.9288
1.8714
1.8603
1.4428
1.4251
1.4075
HO
N
O
CH3
1.04
1.17
1.07
2.03
1.00
1.93
2.10
1.89
2.01
3.01
3.07
6.31
3.49
f1 (ppm)

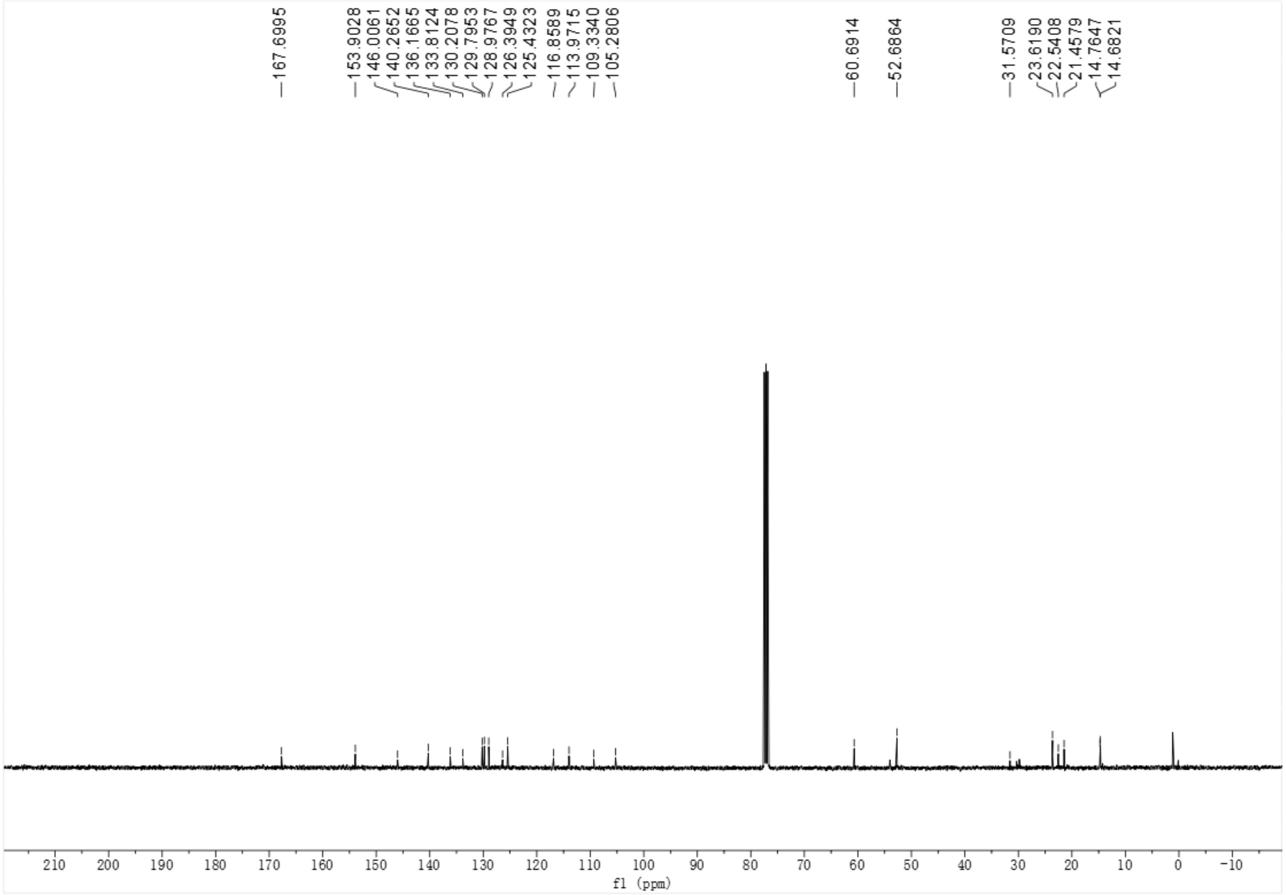

167.6995
153.9028
146.0061
140.2652
136.1665
133.8124
130.2078
129.7953
128.9767
126.3949
125.4323
116.8589
113.9715
109.3340
105.2806
60.6914
52.6864
31.5709
23.6190
22.5408
21.4579
14.7647
14.6821
f1 (ppm)

33

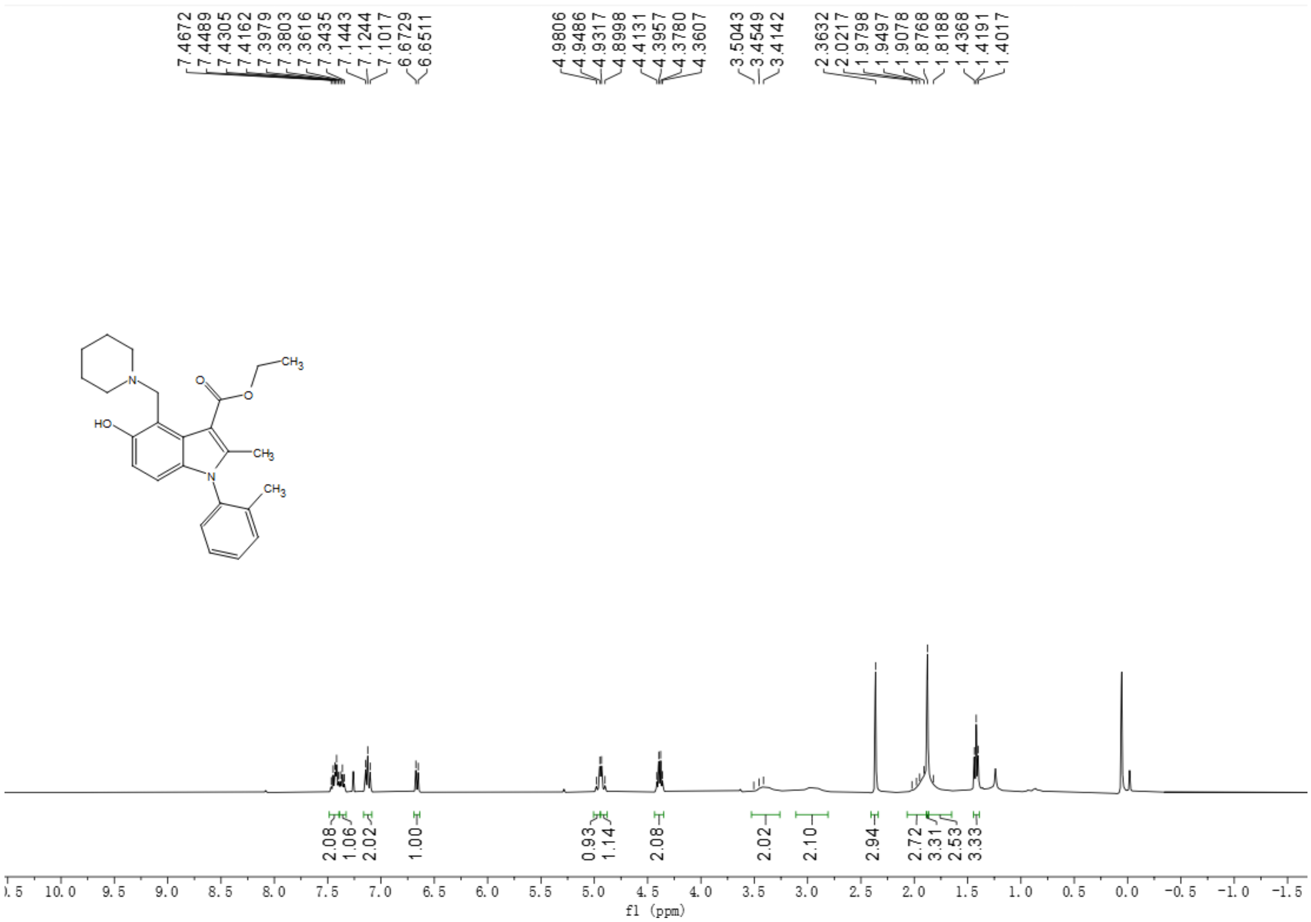

7.4672
7.4489
7.4305
7.4162
7.3979
7.3803
7.3616
7.3435
7.1443
7.1244
7.1017
6.6729
6.6511
4.9806
4.9486
4.9317
4.8998
4.4131
4.3957
4.3780
4.3607
3.5043
3.4549
3.4142
2.3632
2.0217
1.9798
1.9497
1.9078
1.8768
1.8188
1.4368
1.4191
1.4017
HO
N
O
O
CH3
CH3
N
CH3
2.08
1.06
2.02
1.00
0.93
1.14
2.08
2.02
2.10
2.94
2.72
3.31
2.53
3.33
f1 (ppm)

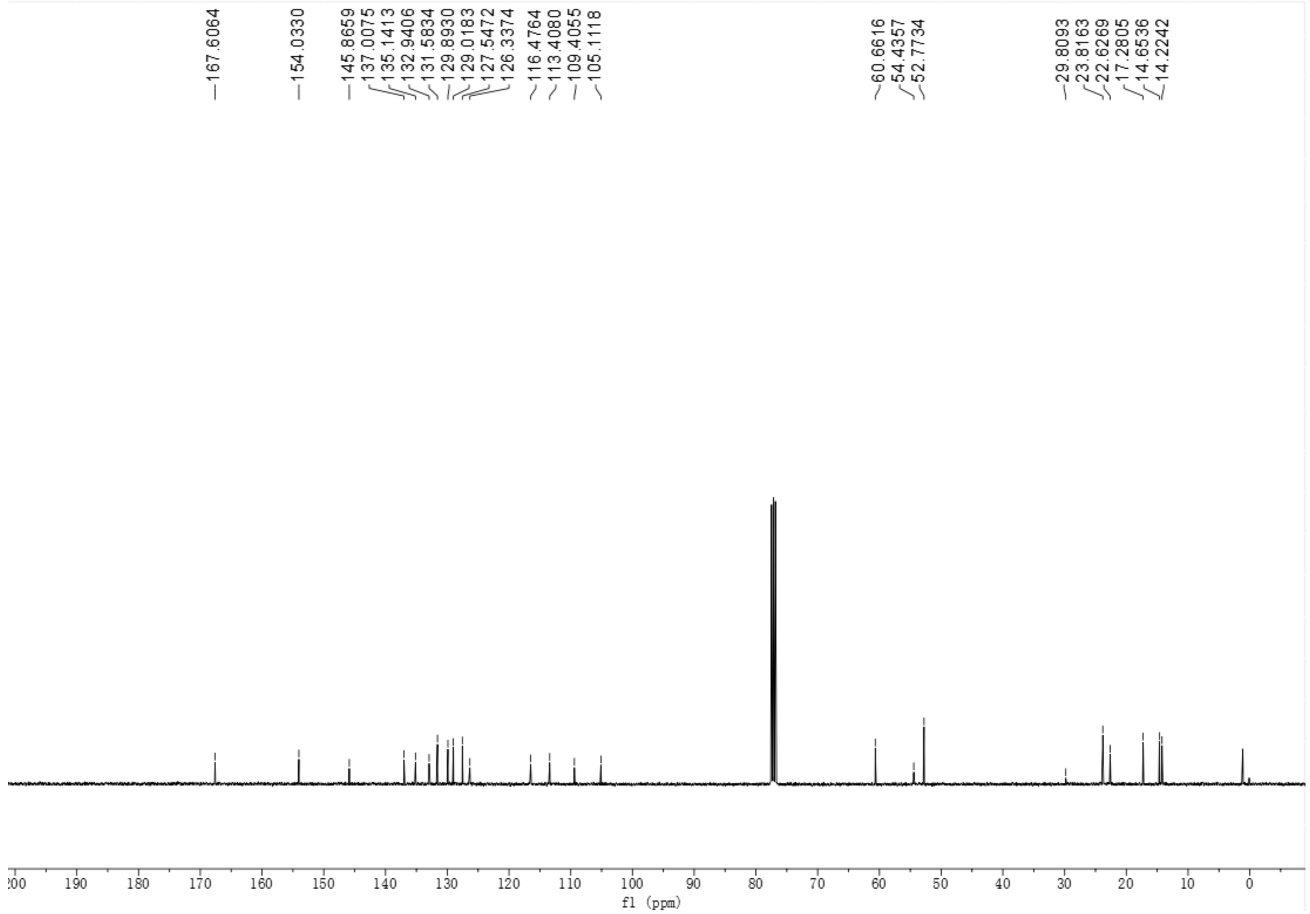

167.6064
154.0330
145.8659
137.0075
135.1413
132.9406
131.5834
129.8930
129.0183
127.5472
126.3374
116.4764
113.4080
109.4055
105.1118
60.6616
54.4357
52.7734
29.8093
23.8163
22.6269
17.2805
14.6536
14.2242
f1 (ppm)

34

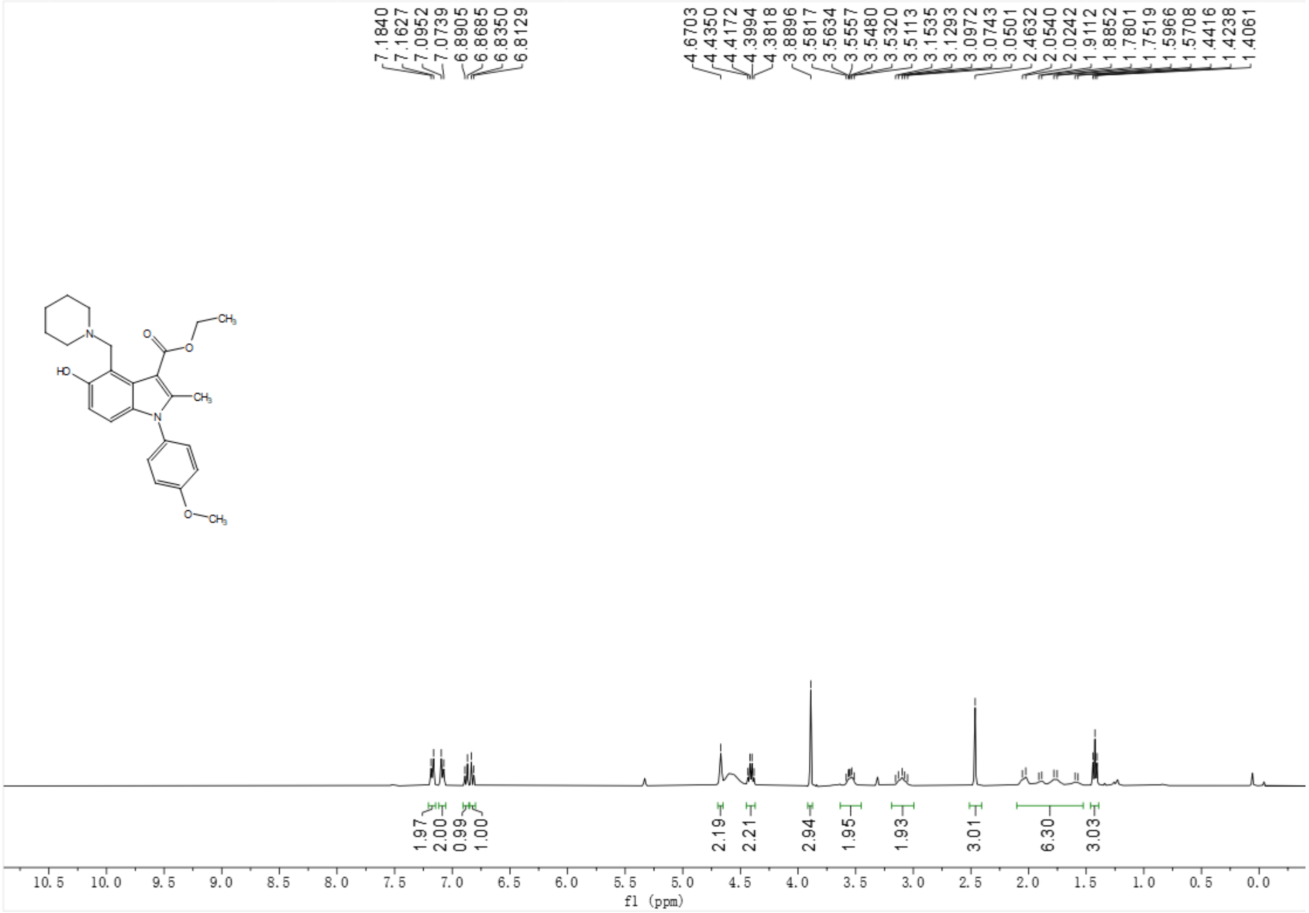
7.1840
7.1627
7.0952
7.0739
6.8905
6.8685
6.8350
6.8129
4.6703
4.4350
4.4172
4.3994
4.3818
3.8896
3.5817
3.5634
3.5557
3.5480
3.5320
3.5113
3.1535
3.1293
3.0972
3.0743
3.0501
2.4632
2.0540
2.0242
1.9112
1.8852
1.7801
1.7519
1.5966
1.5708
1.4416
1.4238
1.4061
HO
O
O
CH3
N
N
O
CH3
1.97
2.00
0.99
1.00
2.19
2.21
2.94
1.95
1.93
3.01
6.30
3.03
10.5 10.0 9.5 9.0 8.5 8.0 7.5 7.0 6.5 6.0 5.5 5.0 4.5 4.0 3.5 3.0 2.5 2.0 1.5 1.0 0.5 0.0
f1 (ppm)

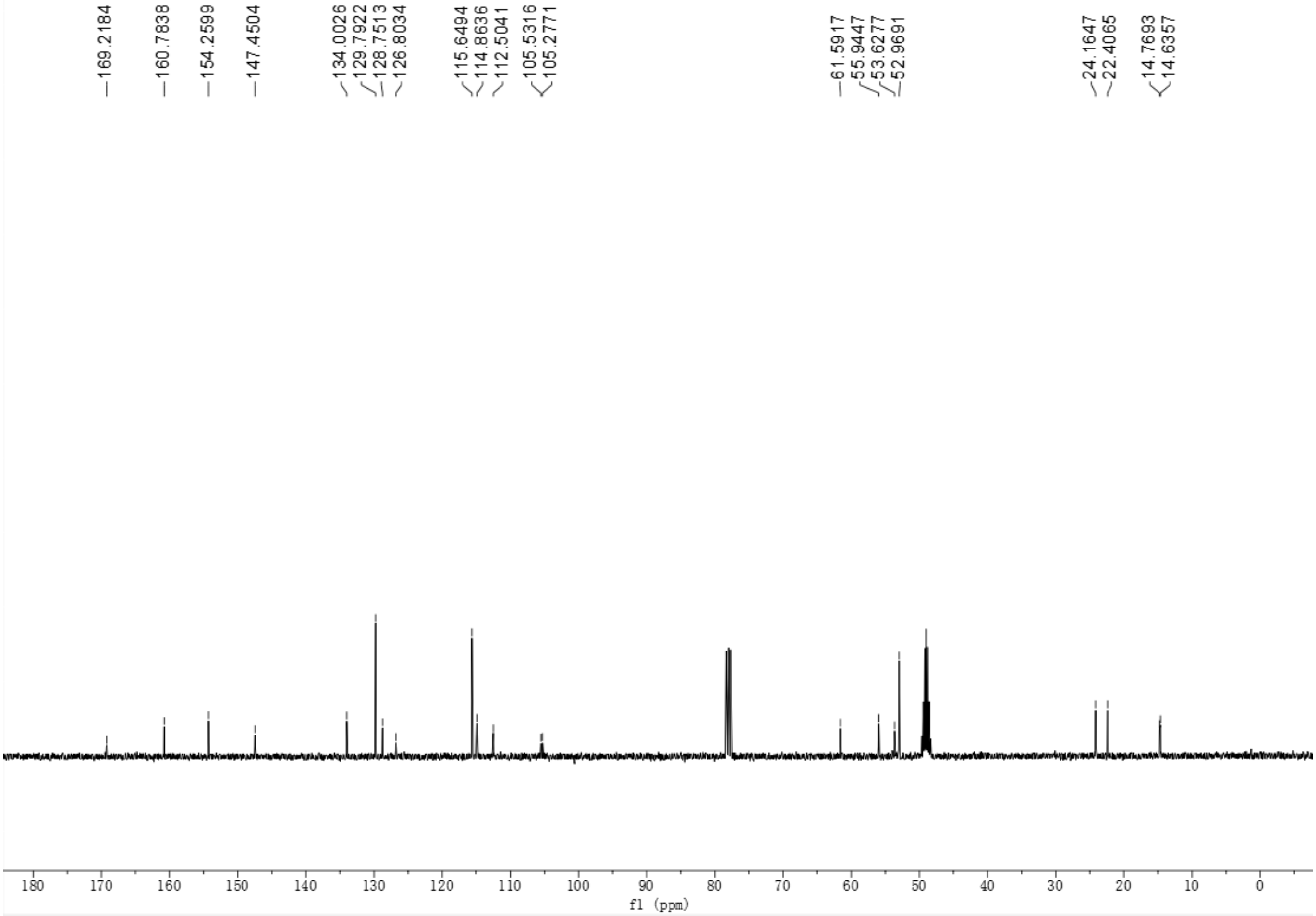
169.2184
160.7838
154.2599
147.4504
134.0026
129.7922
128.7513
126.8034
115.6494
114.8636
112.5041
105.5316
105.2771
61.5917
55.9447
53.6277
52.9691
24.1647
22.4065
14.7693
14.6357
180 170 160 150 140 130 120 110 100 90 80 70 60 50 40 30 20 10 0
f1 (ppm)

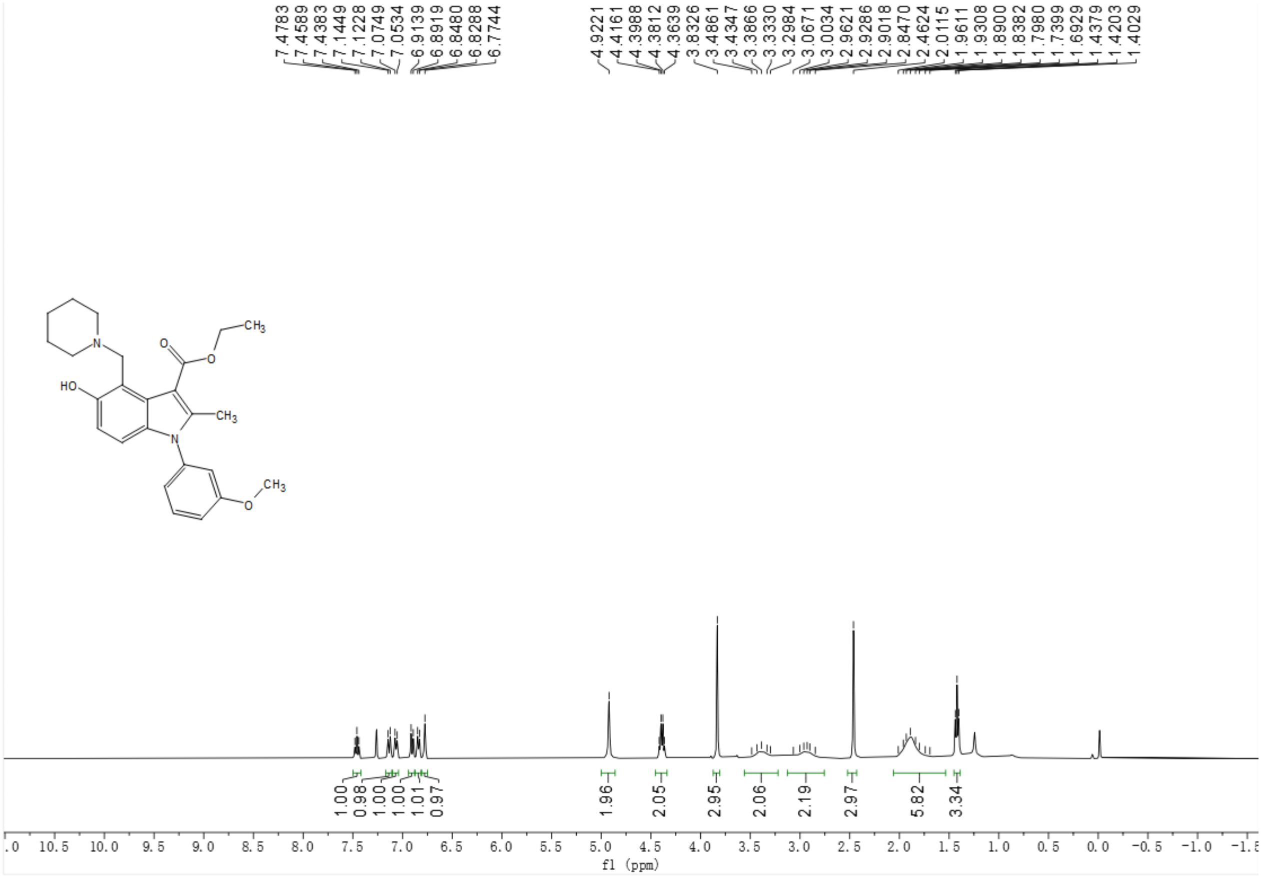

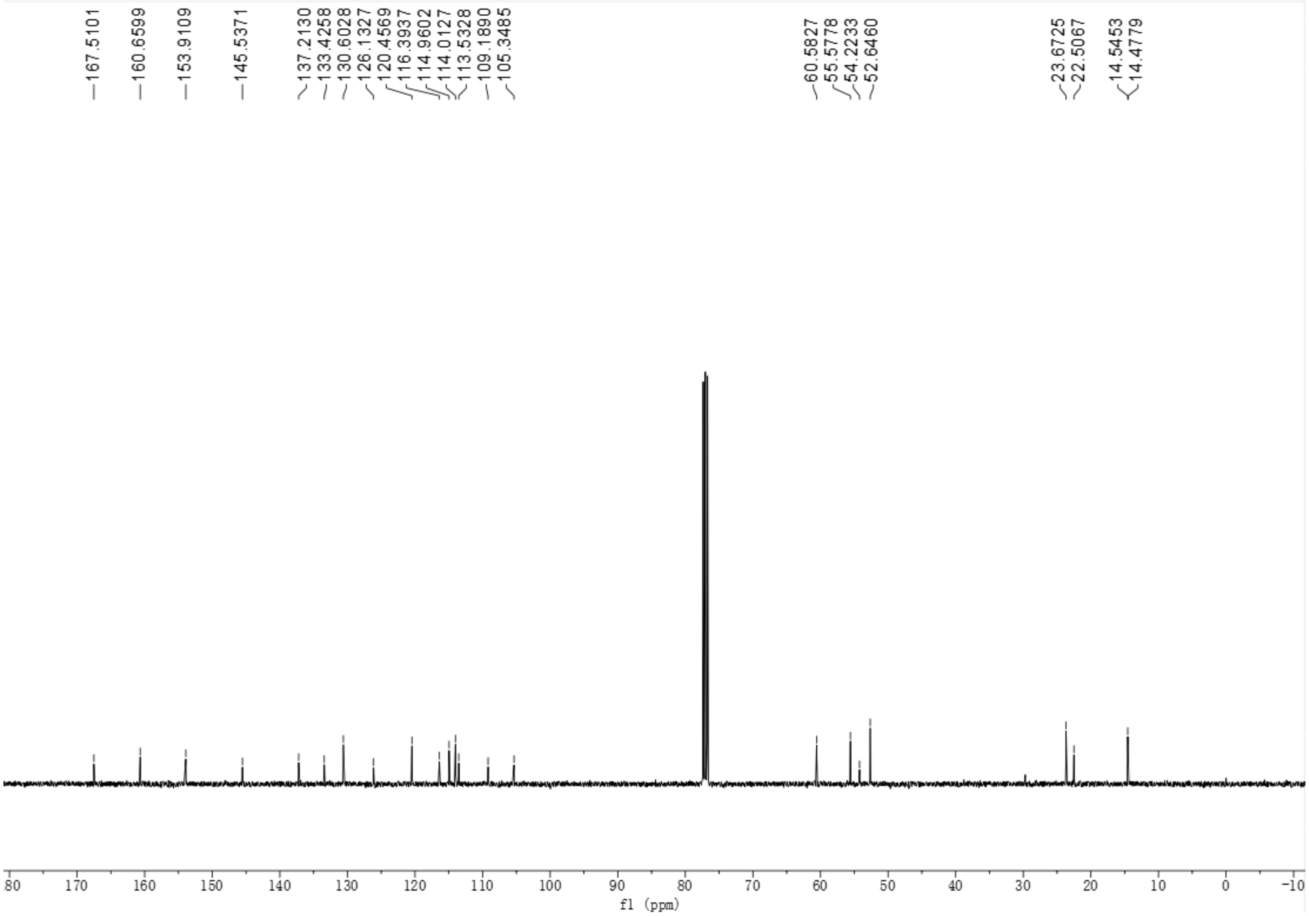

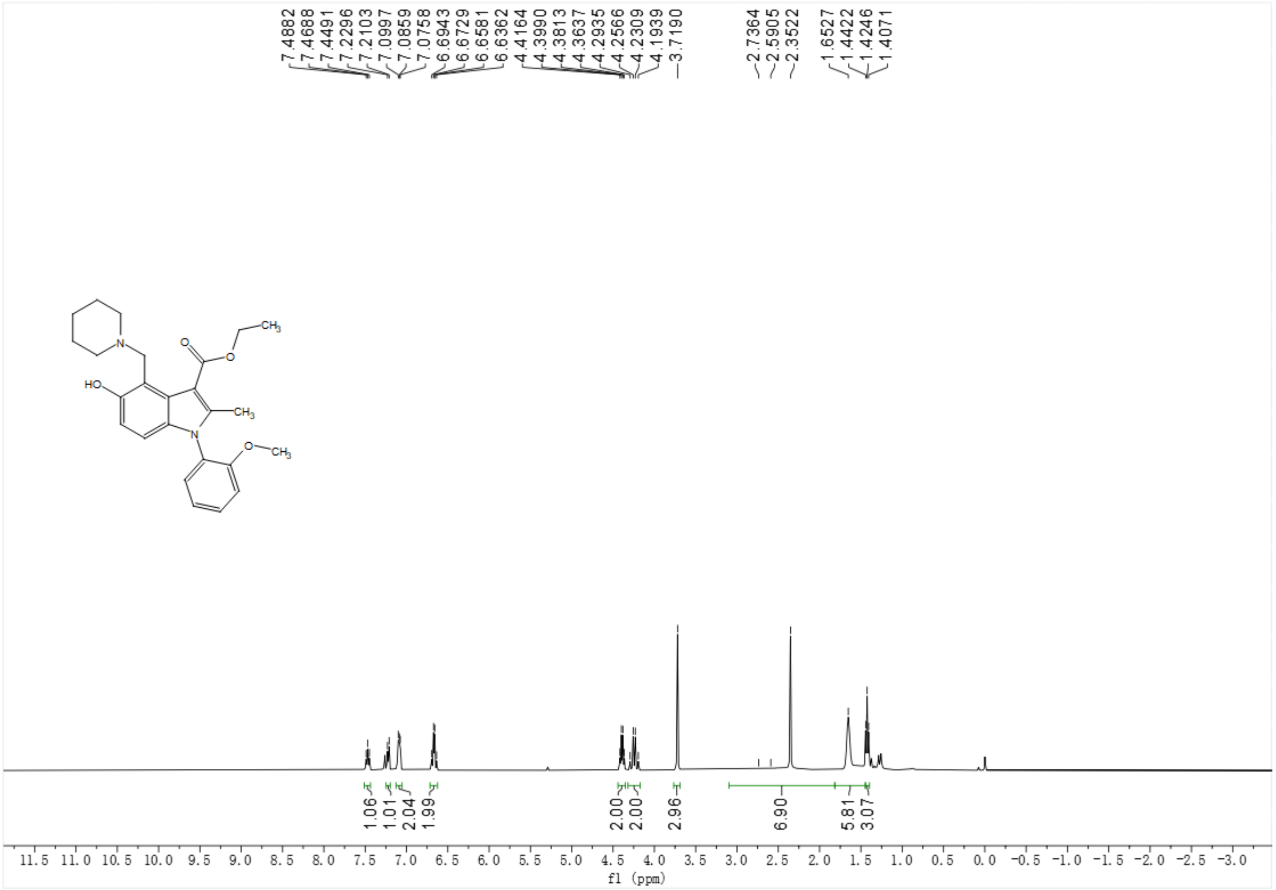
7.4882
7.4688
7.4491
7.2296
7.2103
7.0997
7.0859
7.0758
6.6943
6.6729
6.6581
6.6362
4.4164
4.3990
4.3813
4.3637
4.2935
4.2566
4.2309
4.1939
3.7190
2.7364
2.5905
2.3522
1.6527
1.4422
1.4246
1.4071
HO
CH3
O
N
1.06
1.01
2.04
1.99
2.00
2.00
2.96
6.90
5.81
3.07
f1 (ppm)

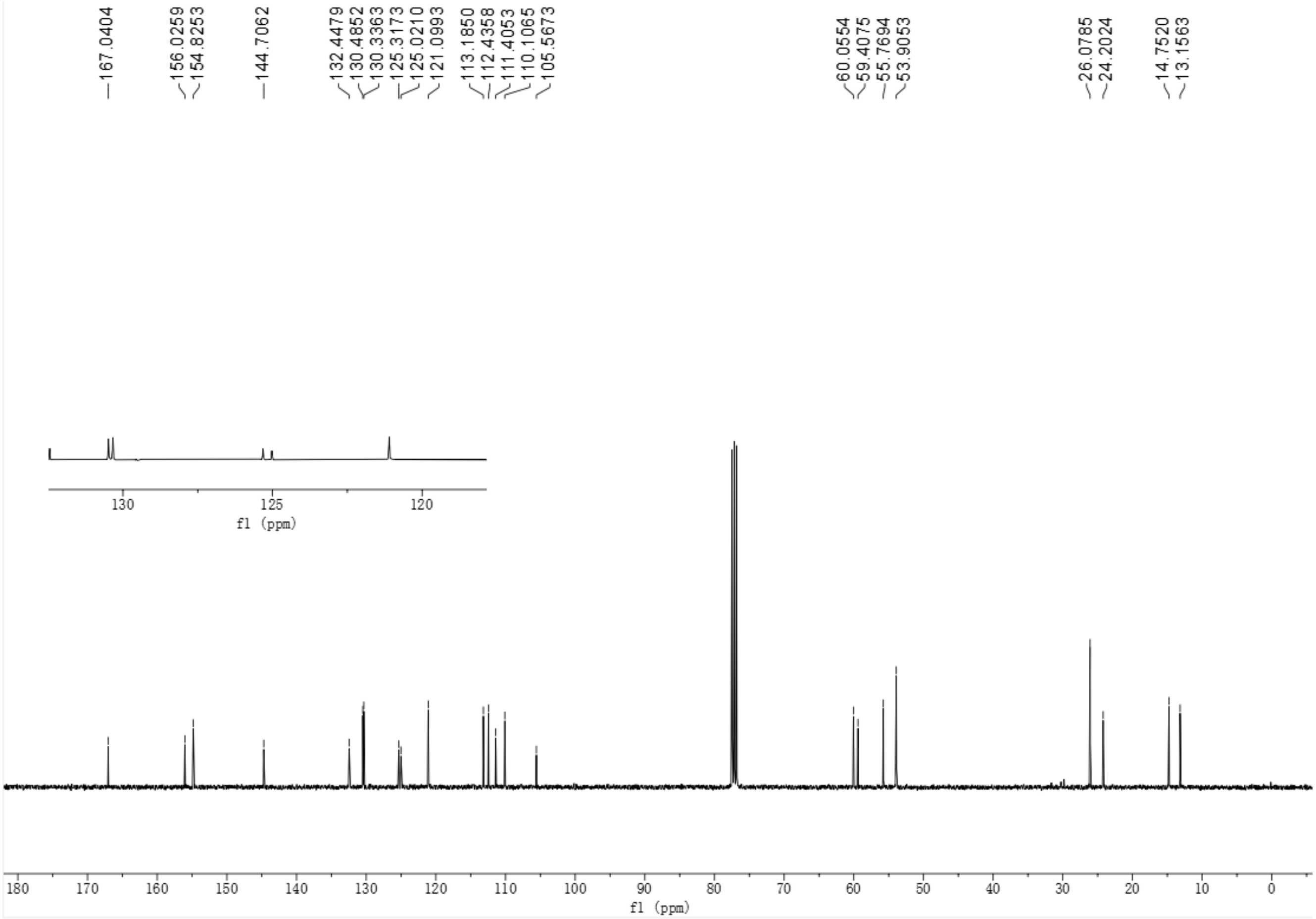
167.0404
156.0259
154.8253
144.7062
132.4479
130.4852
130.3363
125.3173
125.0210
121.0993
113.1850
112.4358
111.4053
110.1065
105.5673
60.0554
59.4075
55.7694
53.9053
26.0785
24.2024
14.7520
13.1563
f1 (ppm)

37

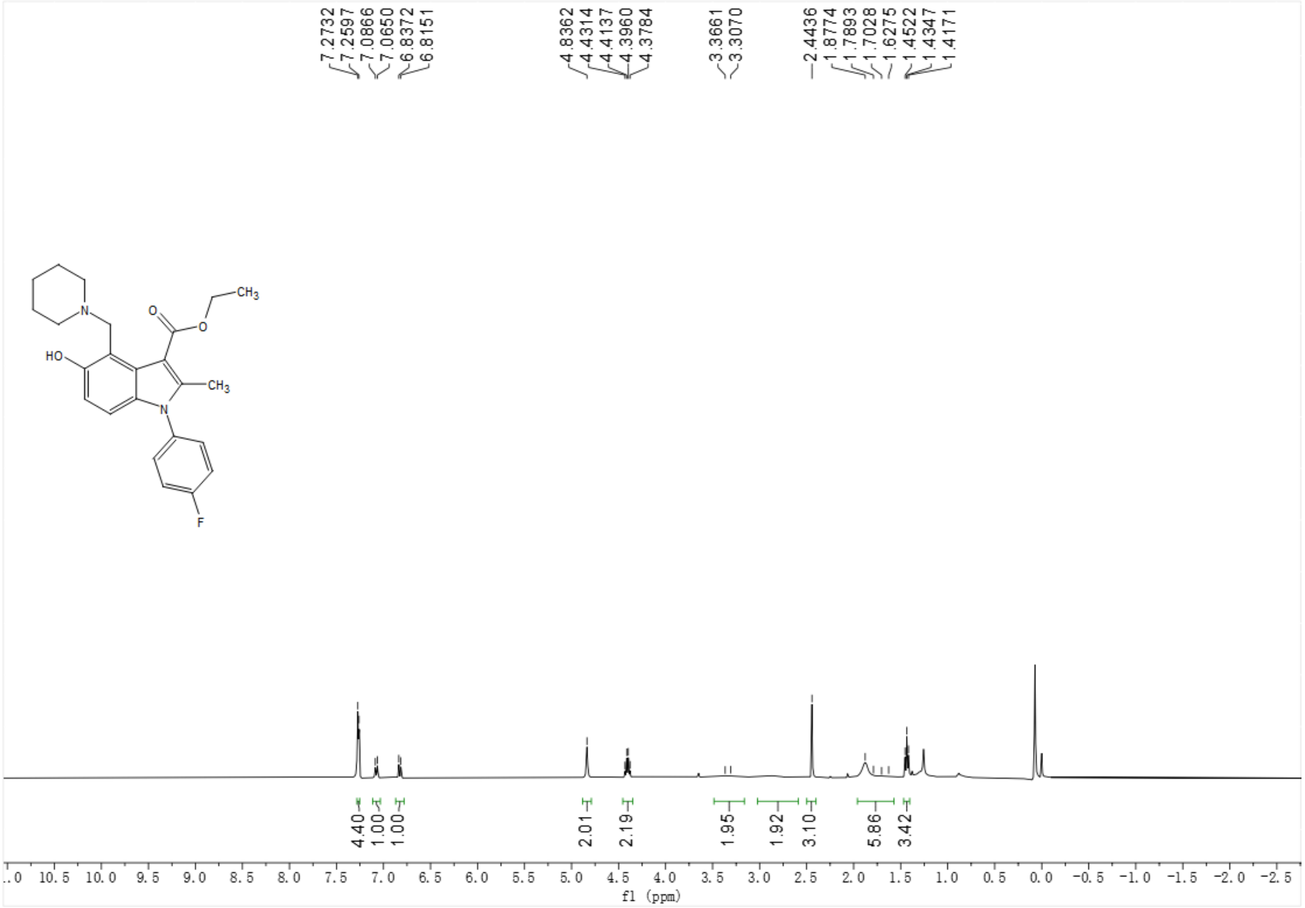
7.2732
7.2597
7.0866
7.0650
6.8372
6.8151
4.8362
4.4314
4.4137
4.3960
4.3784
3.3661
3.3070
2.4436
1.8774
1.7893
1.7028
1.6275
1.4522
1.4347
1.4171
4.40
1.00
1.00
2.01
2.19
1.95
1.92
3.10
5.86
3.42
f1 (ppm)

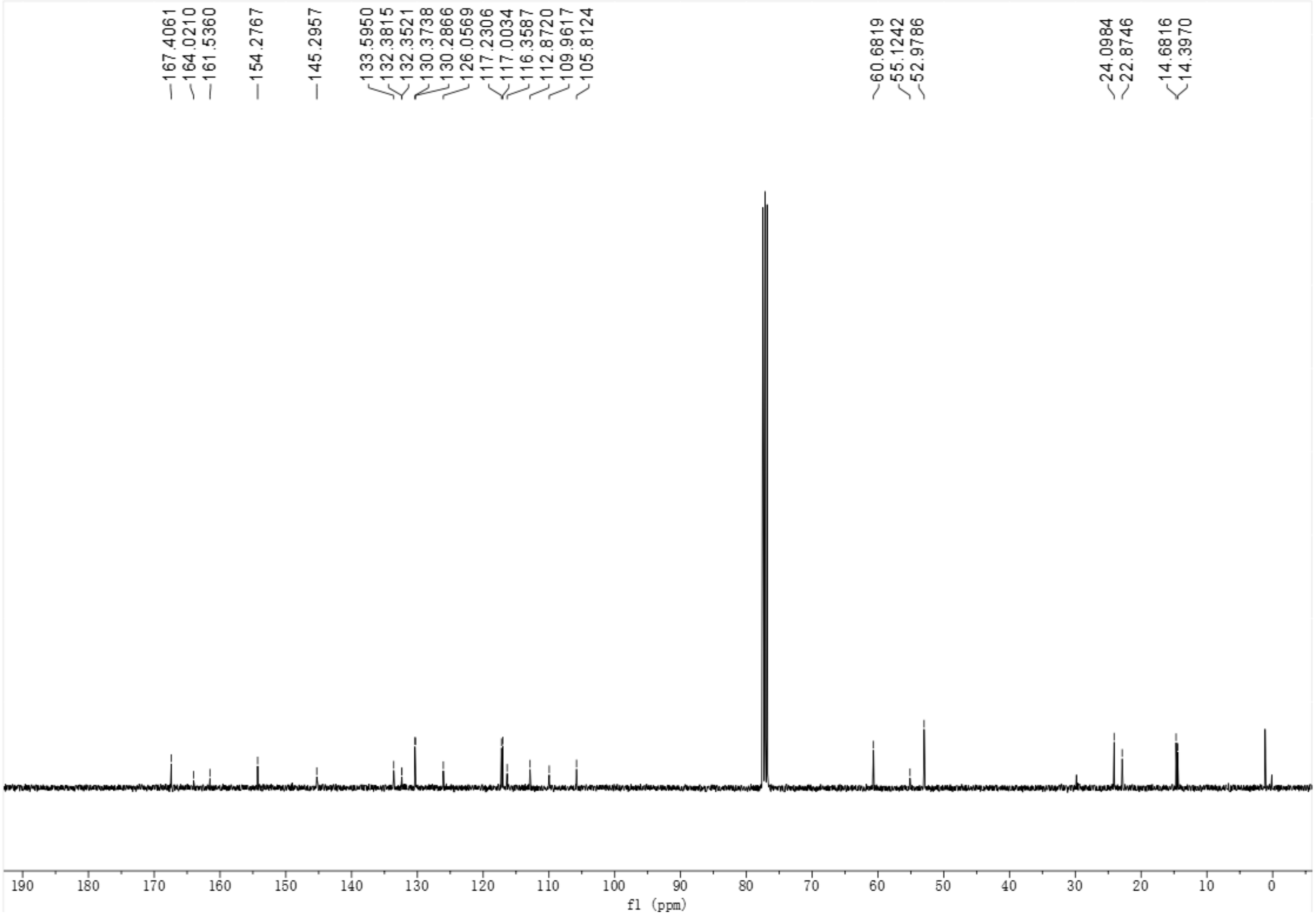
167.4061
164.0210
161.5360
154.2767
145.2957
133.5950
132.3815
132.3521
130.3738
130.2866
126.0569
117.2306
117.0034
116.3587
112.8720
109.9617
105.8124
60.6819
55.1242
52.9786
24.0984
22.8746
14.6816
14.3970
f1 (ppm)

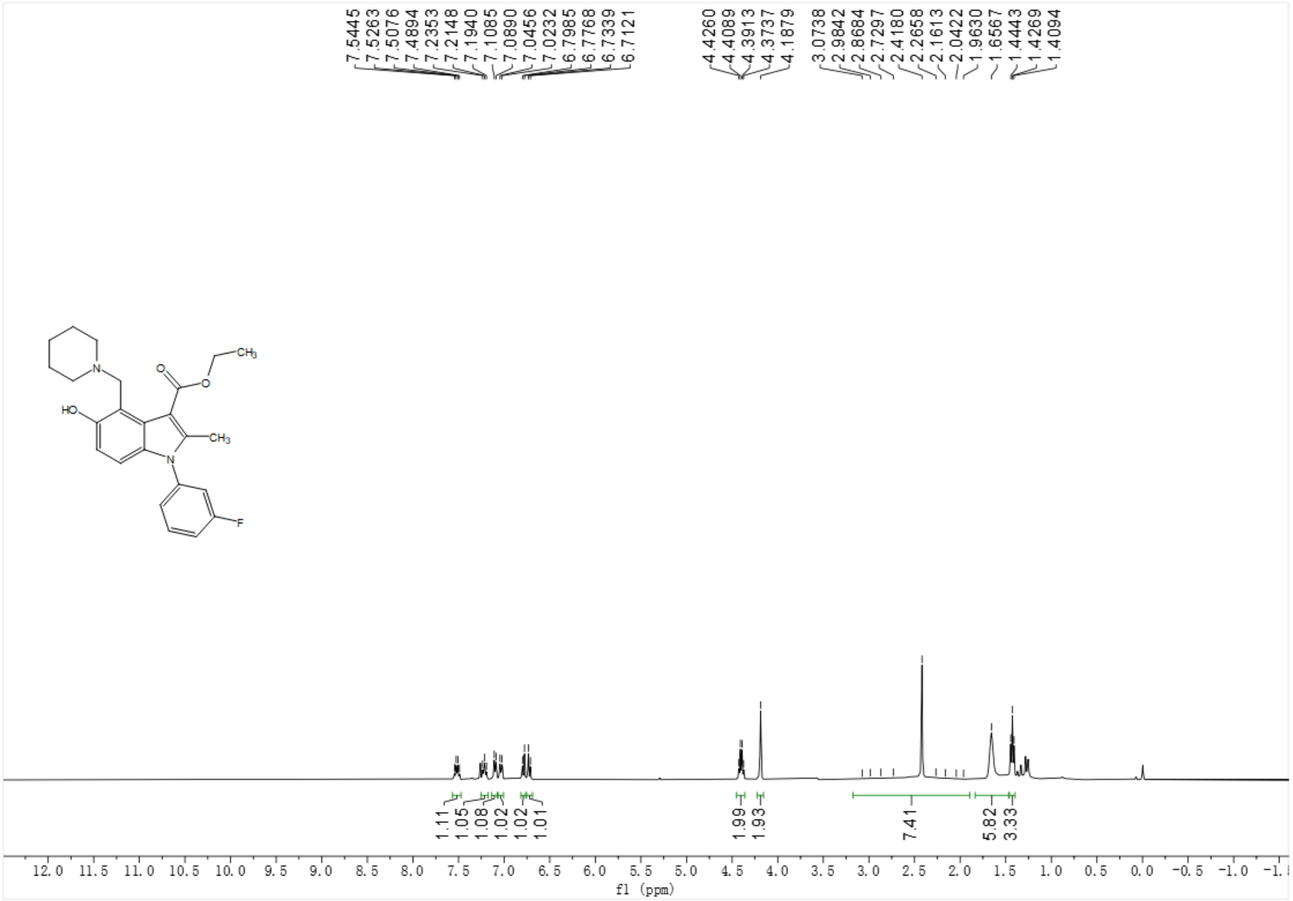
7.5445
7.5263
7.5076
7.4894
7.2353
7.2148
7.1940
7.1085
7.0890
7.0456
7.0232
6.7985
6.7768
6.7339
6.7121
4.4260
4.4089
4.3913
4.3737
4.1879
3.0738
2.9842
2.8684
2.7297
2.4180
2.2658
2.1613
2.0422
1.9630
1.6567
1.4443
1.4269
1.4094
N
O
O
CH3
HO
CH3
N
F
1.11
1.05
1.08
1.02
1.01
1.99
1.93
7.41
5.82
3.33
f1 (ppm)

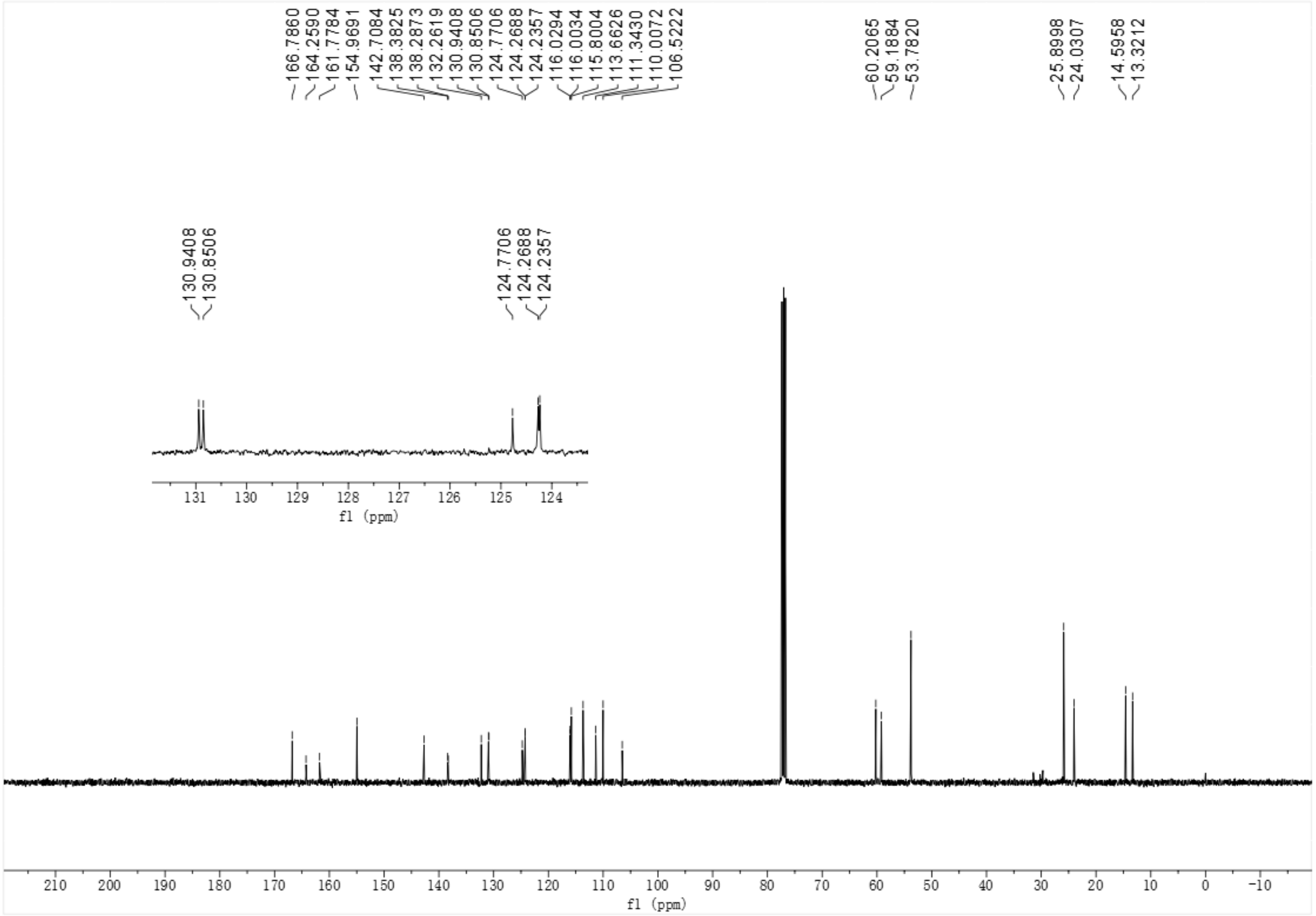
166.7860
164.2590
161.7784
154.9691
142.7084
138.3825
138.2873
132.2619
130.9408
130.8506
124.7706
124.2688
124.2357
116.0294
116.0034
115.8004
113.6626
111.3430
110.0072
106.5222
60.2065
59.1884
53.7820
25.8998
24.0307
14.5958
13.3212
130.9408
130.8506
124.7706
124.2688
124.2357
f1 (ppm)
f1 (ppm)

39

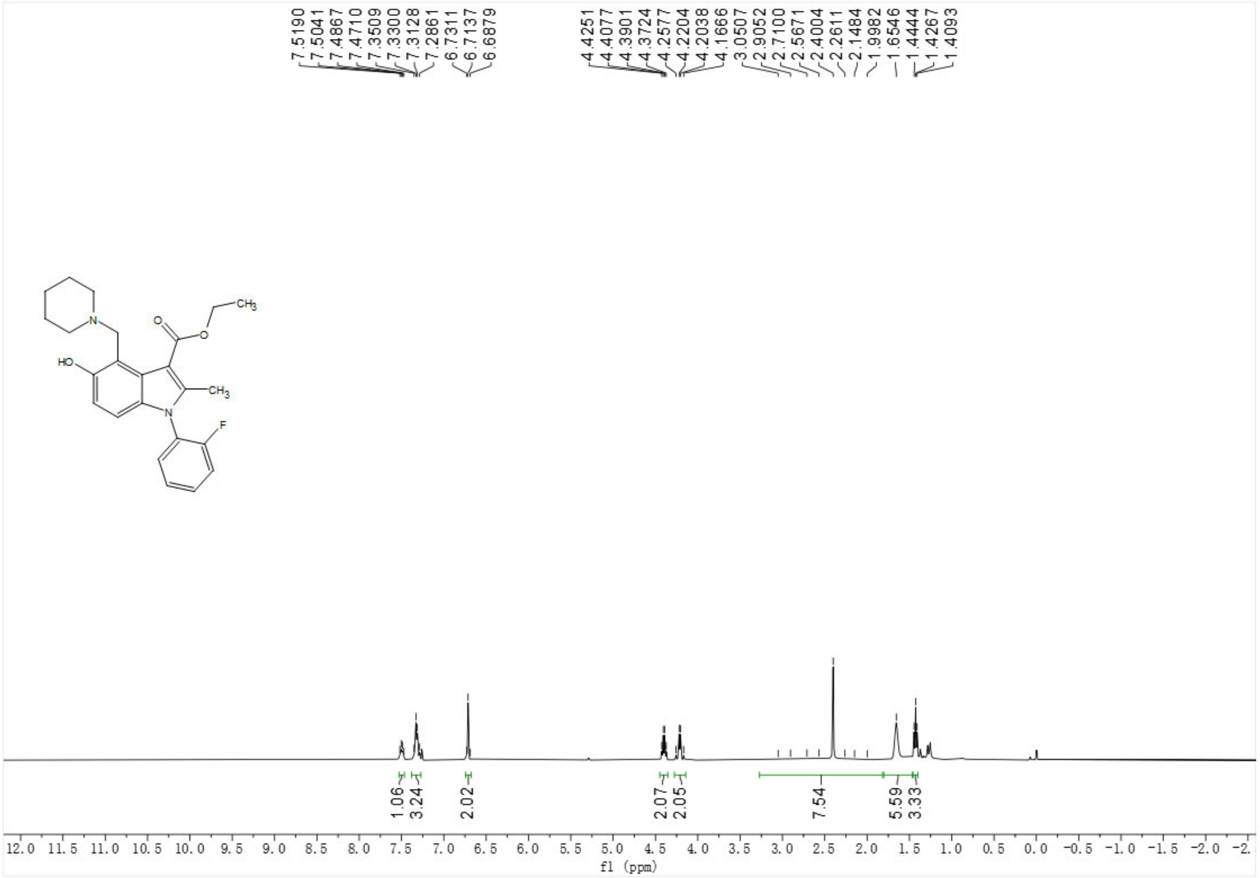
7.5190
7.5041
7.4867
7.4710
7.3509
7.3300
7.3128
7.2861
6.7311
6.7137
6.6879
4.4251
4.4077
4.3901
4.3724
4.2577
4.2204
4.2038
4.1666
3.0507
2.9052
2.7100
2.5671
2.4004
2.2611
2.1484
1.9982
1.6546
1.4444
1.4267
1.4093
1.06
3.24
2.02
2.07
2.05
7.54
5.59
3.33
12.0 11.5 11.0 10.5 10.0 9.5 9.0 8.5 8.0 7.5 7.0 6.5 6.0 5.5 5.0 4.5 4.0 3.5 3.0 2.5 2.0 1.5 1.0 0.5 0.0 -0.5 -1.0 -1.5 -2.0 -2.
f1 (ppm)

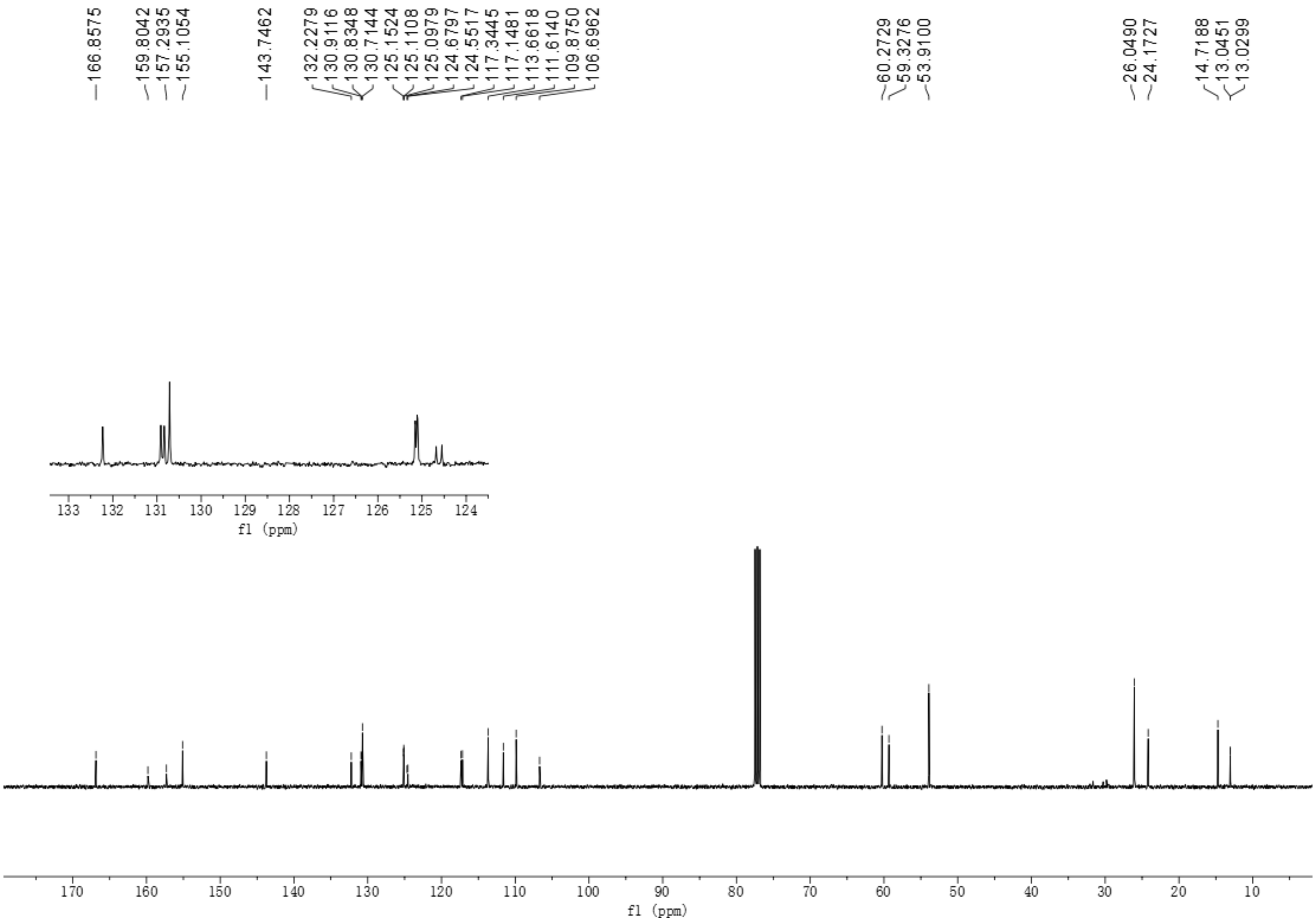
166.8575
159.8042
157.2935
155.1054
143.7462
132.2279
130.9116
130.8348
130.7144
125.1524
125.1108
125.0979
124.6797
124.5517
117.3445
117.1481
113.6618
111.6140
109.8750
106.6962
60.2729
59.3276
53.9100
26.0490
24.1727
14.7188
13.0451
13.0299
133 132 131 130 129 128 127 126 125 124
f1 (ppm)
170 160 150 140 130 120 110 100 90 80 70 60 50 40 30 20 10
f1 (ppm)

45

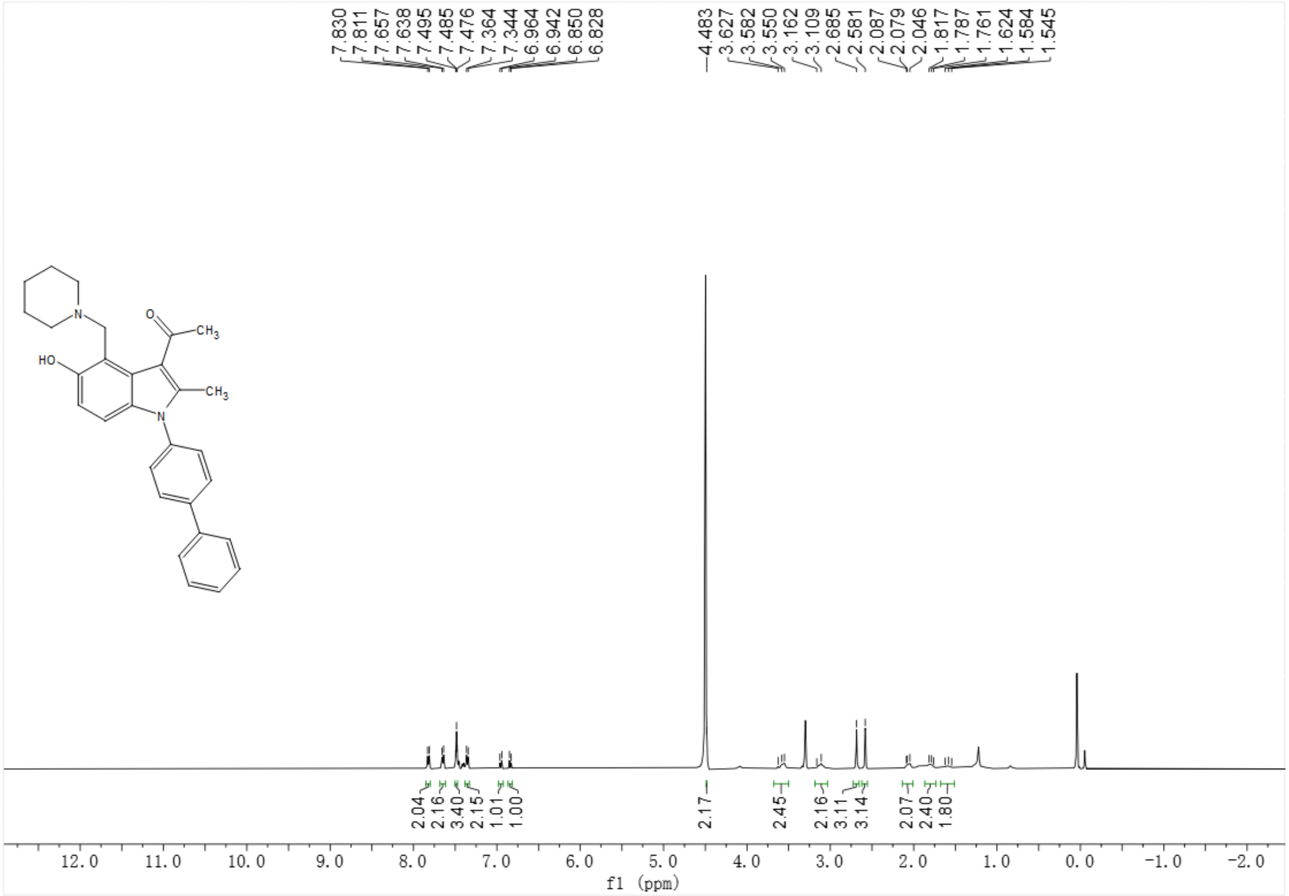

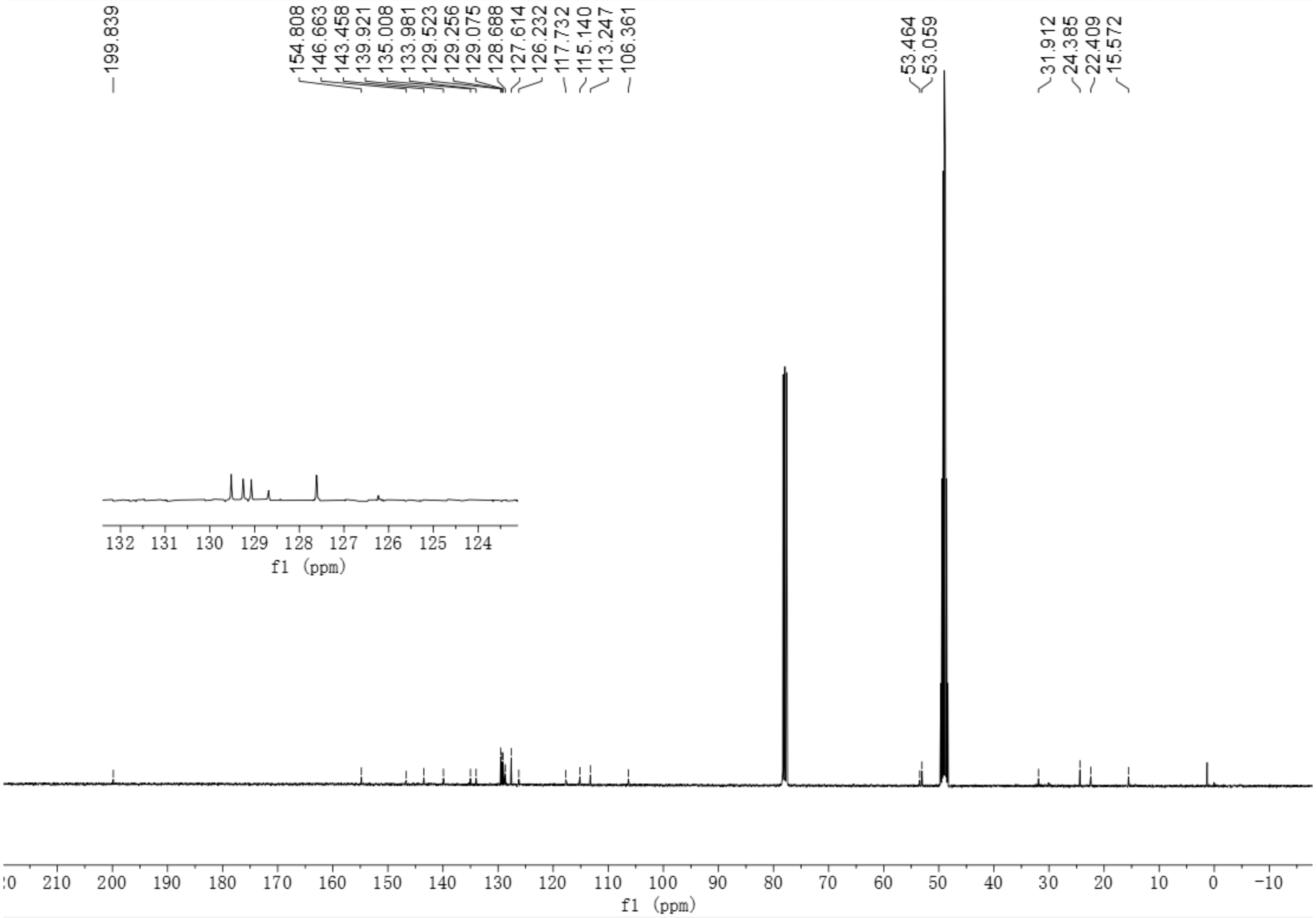

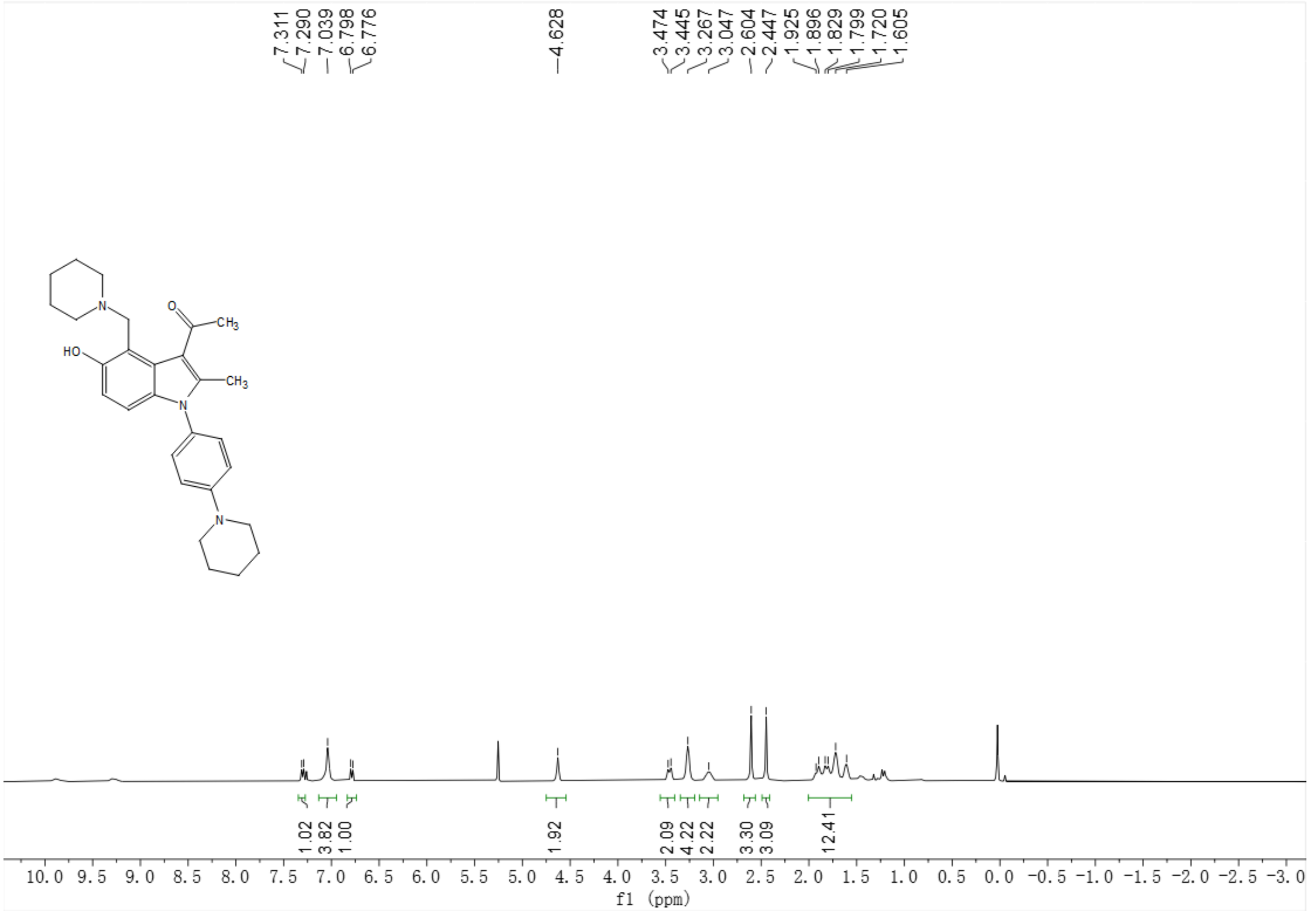
7.311
7.290
7.039
6.798
6.776
4.628
3.474
3.445
3.267
3.047
2.604
2.447
1.925
1.896
1.829
1.799
1.720
1.605
HO
O
CH3
CH3
N
N
N
1.02
3.82
1.00
1.92
2.09
4.22
2.22
3.30
3.09
12.41
10.0 9.5 9.0 8.5 8.0 7.5 7.0 6.5 6.0 5.5 5.0 4.5 4.0 3.5 3.0 2.5 2.0 1.5 1.0 0.5 0.0 -0.5 -1.0 -1.5 -2.0 -2.5 -3.0
f1 (ppm)

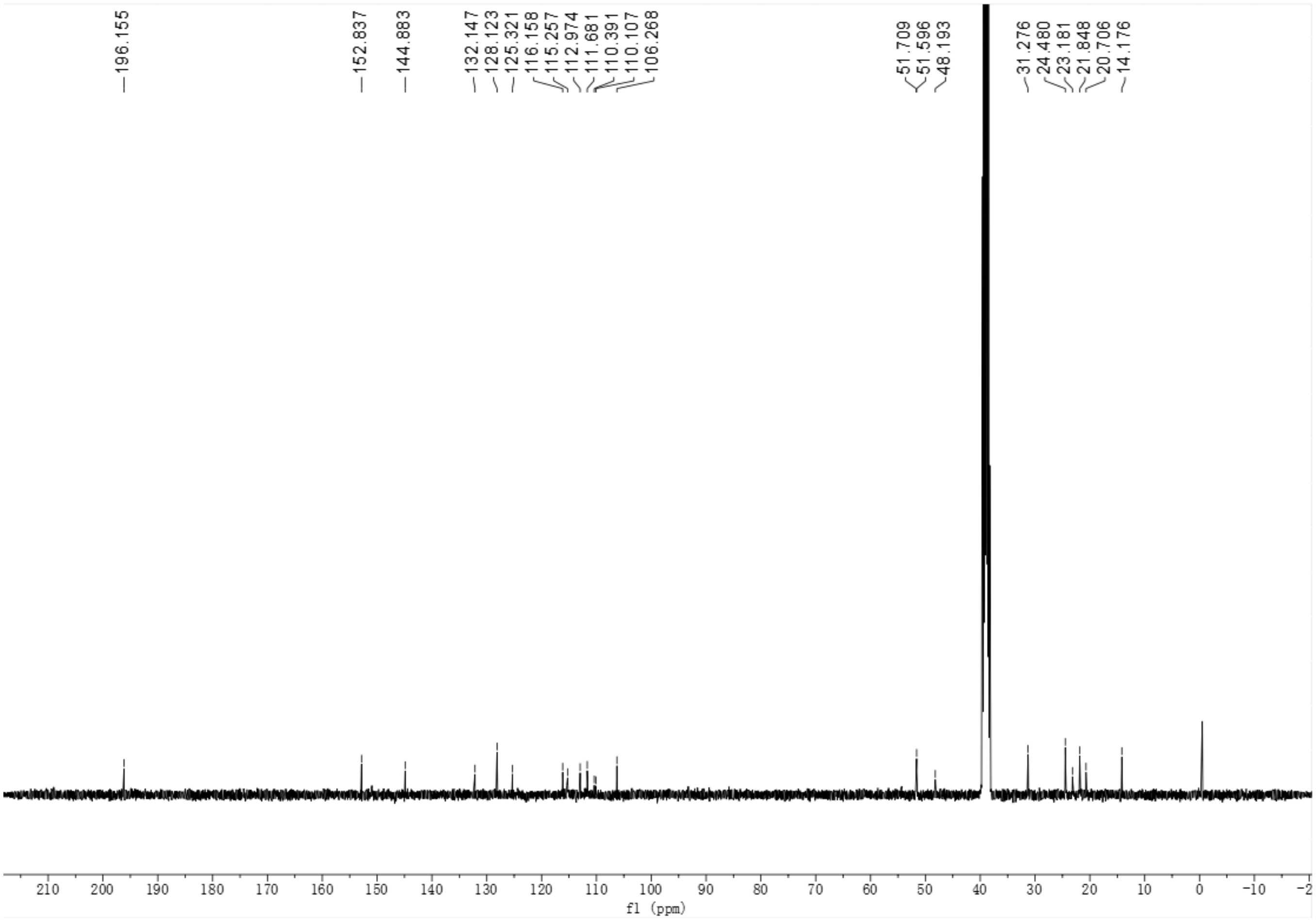
196.155
152.837
144.883
132.147
128.123
125.321
116.158
115.257
112.974
111.681
110.391
110.107
106.268
51.709
51.596
48.193
31.276
24.480
23.181
21.848
20.706
14.176
210 200 190 180 170 160 150 140 130 120 110 100 90 80 70 60 50 40 30 20 10 0 -10 -2
f1 (ppm)

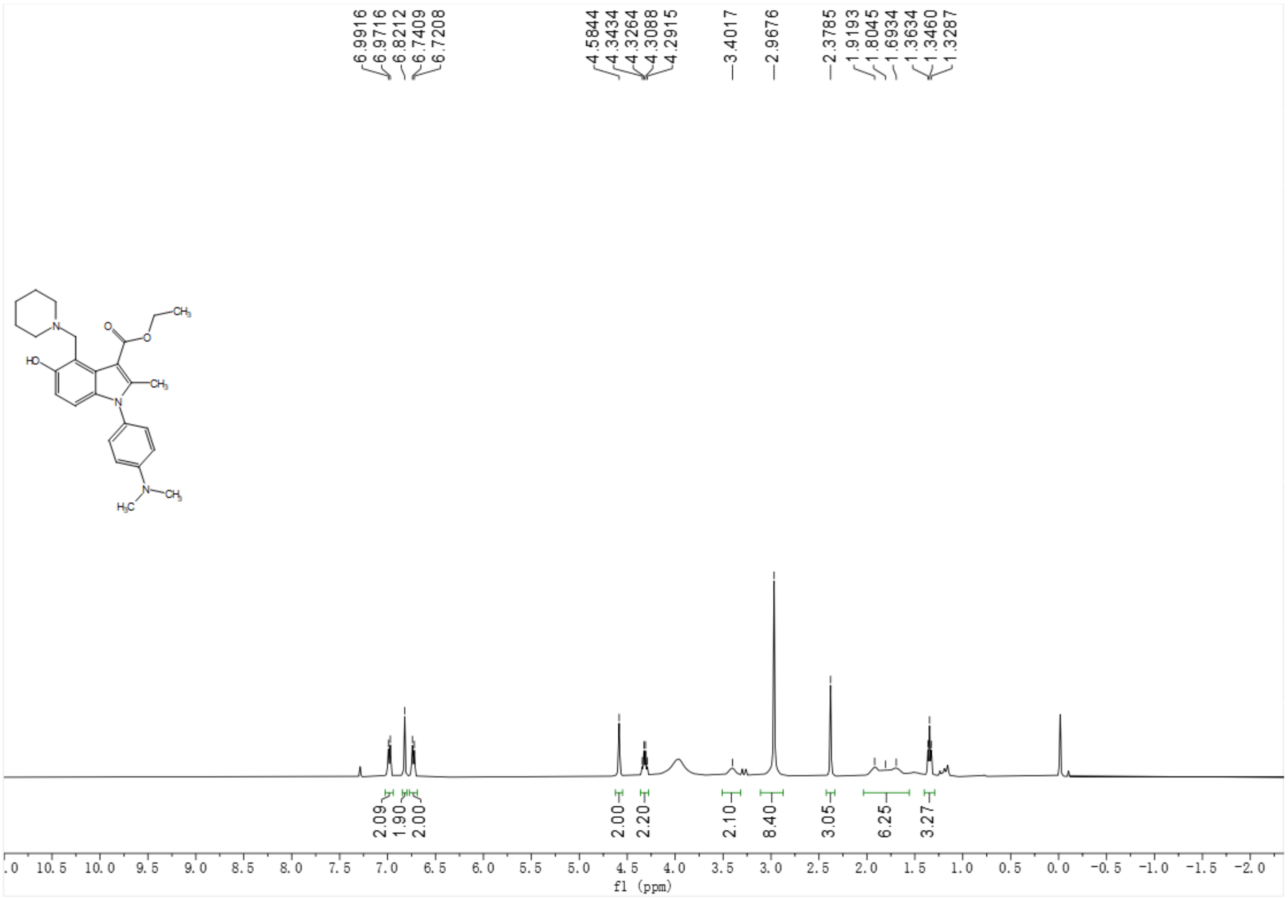
6.9916
6.9716
6.8212
6.7409
6.7208
4.5844
4.3434
4.3264
4.3088
4.2915
3.4017
2.9676
2.3785
1.9193
1.8045
1.6934
1.3634
1.3460
1.3287
HO
N
O
O
CH3
CH3
N
H3C
CH3
2.09
1.90
2.00
2.00
2.20
2.10
8.40
3.05
6.25
3.27
f1 (ppm)

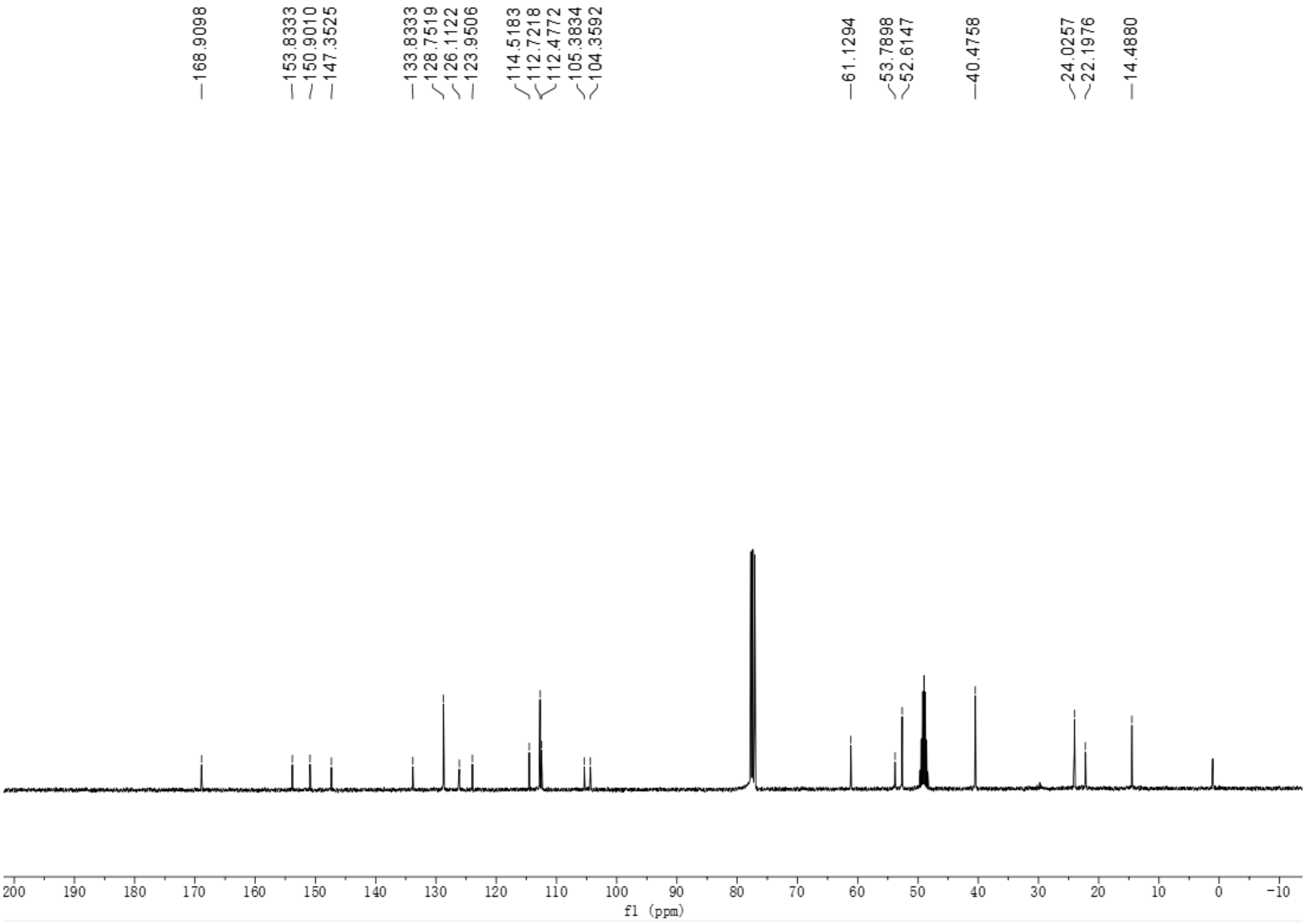
168.9098
153.8333
150.9010
147.3525
133.8333
128.7519
126.1122
123.9506
114.5183
112.7218
112.4772
105.3834
104.3592
61.1294
53.7898
52.6147
40.4758
24.0257
22.1976
14.4880
f1 (ppm)

50

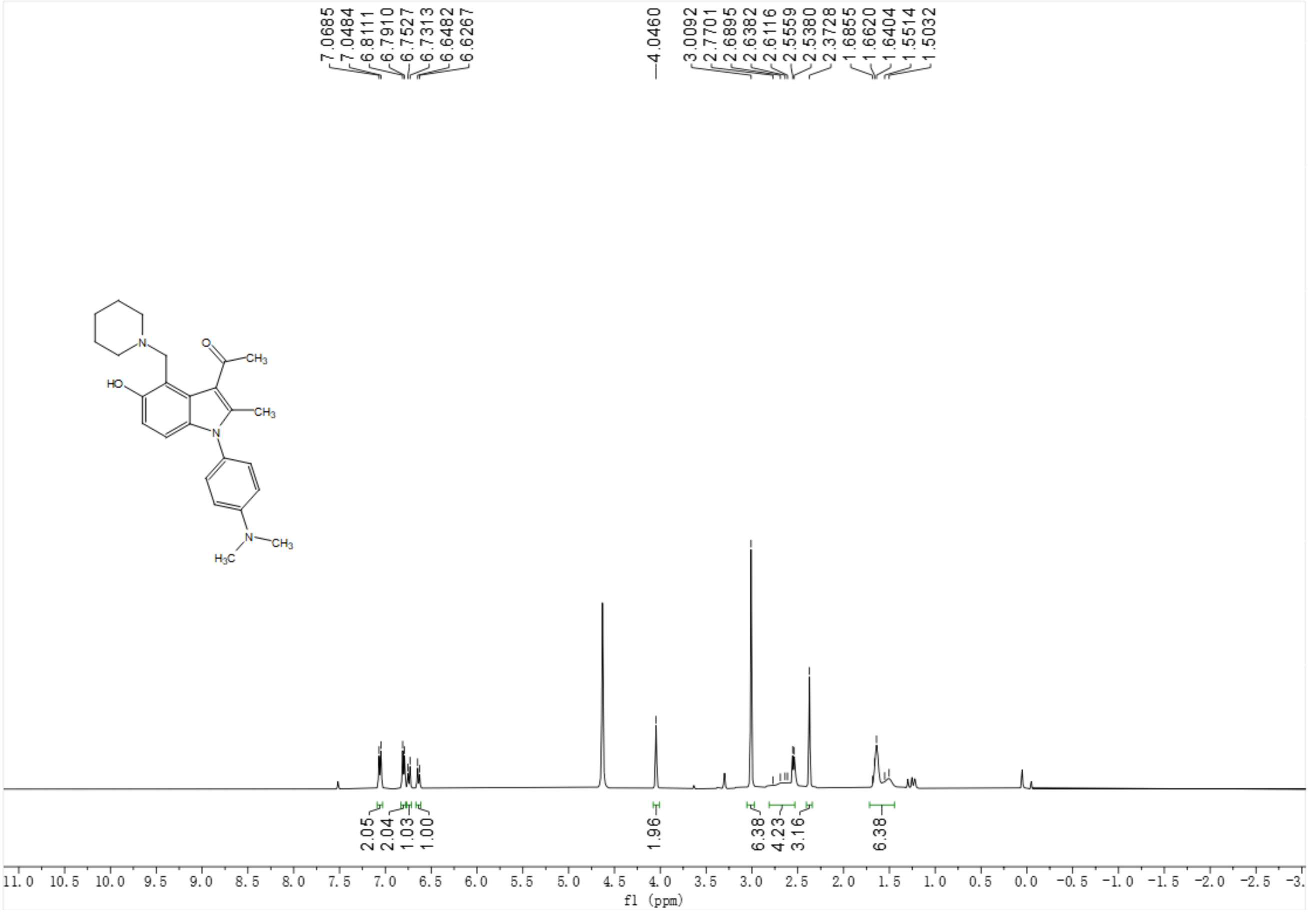

7.0685
7.0484
6.8111
6.7910
6.7527
6.7313
6.6482
6.6267
4.0460
3.0092
2.7701
2.6895
2.6382
2.6116
2.5559
2.5380
2.3728
1.6855
1.6620
1.6404
1.5514
1.5032
2.05
2.04
1.03
1.00
1.96
6.38
4.23
3.16
6.38
HO
N
O
CH3
CH3
N
CH3
H3C
f1 (ppm)

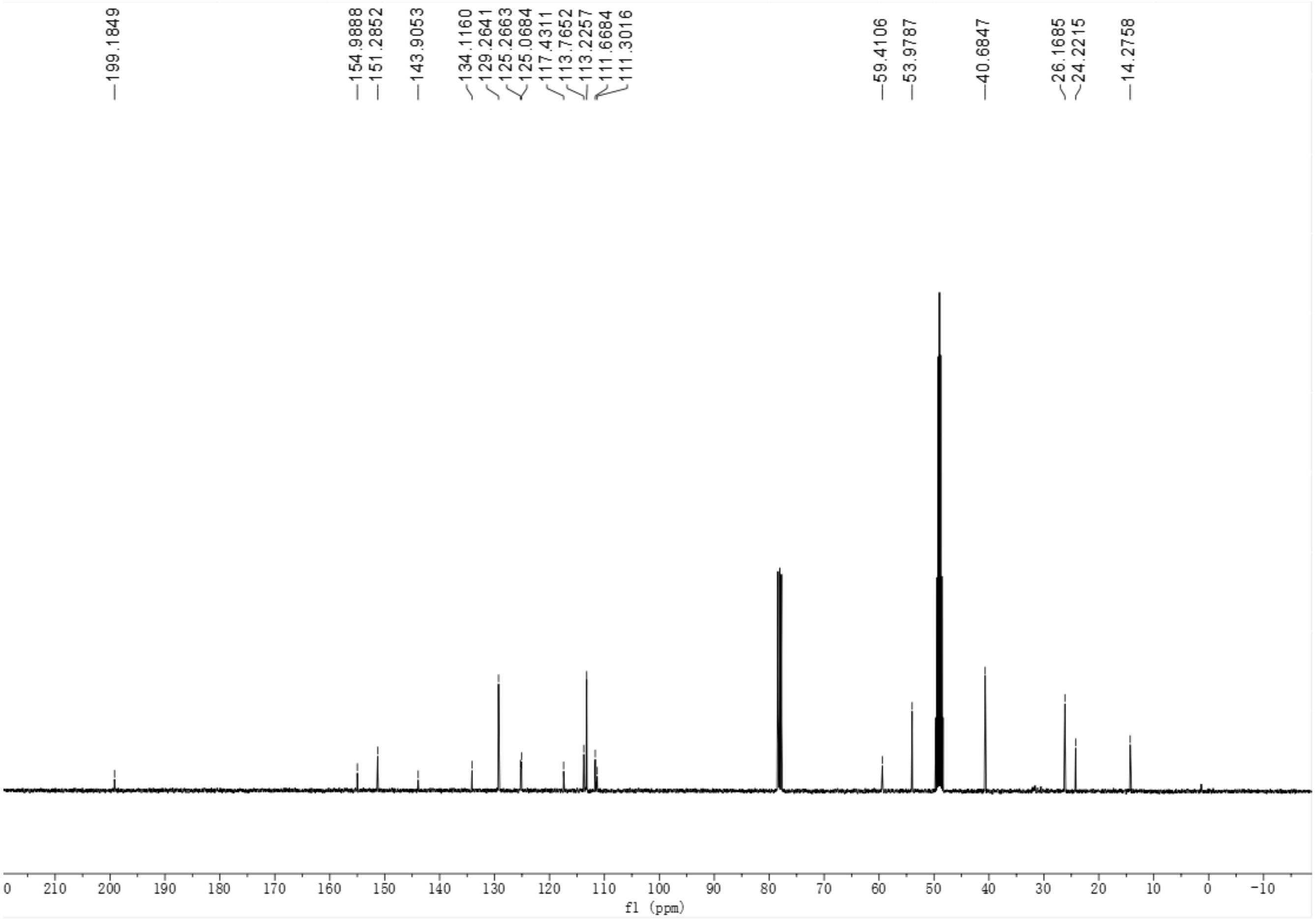

199.1849
154.9888
151.2852
143.9053
134.1160
129.2641
125.2663
125.0684
117.4311
113.7652
113.2257
111.6684
111.3016
59.4106
53.9787
40.6847
26.1685
24.2215
14.2758
f1 (ppm)

51

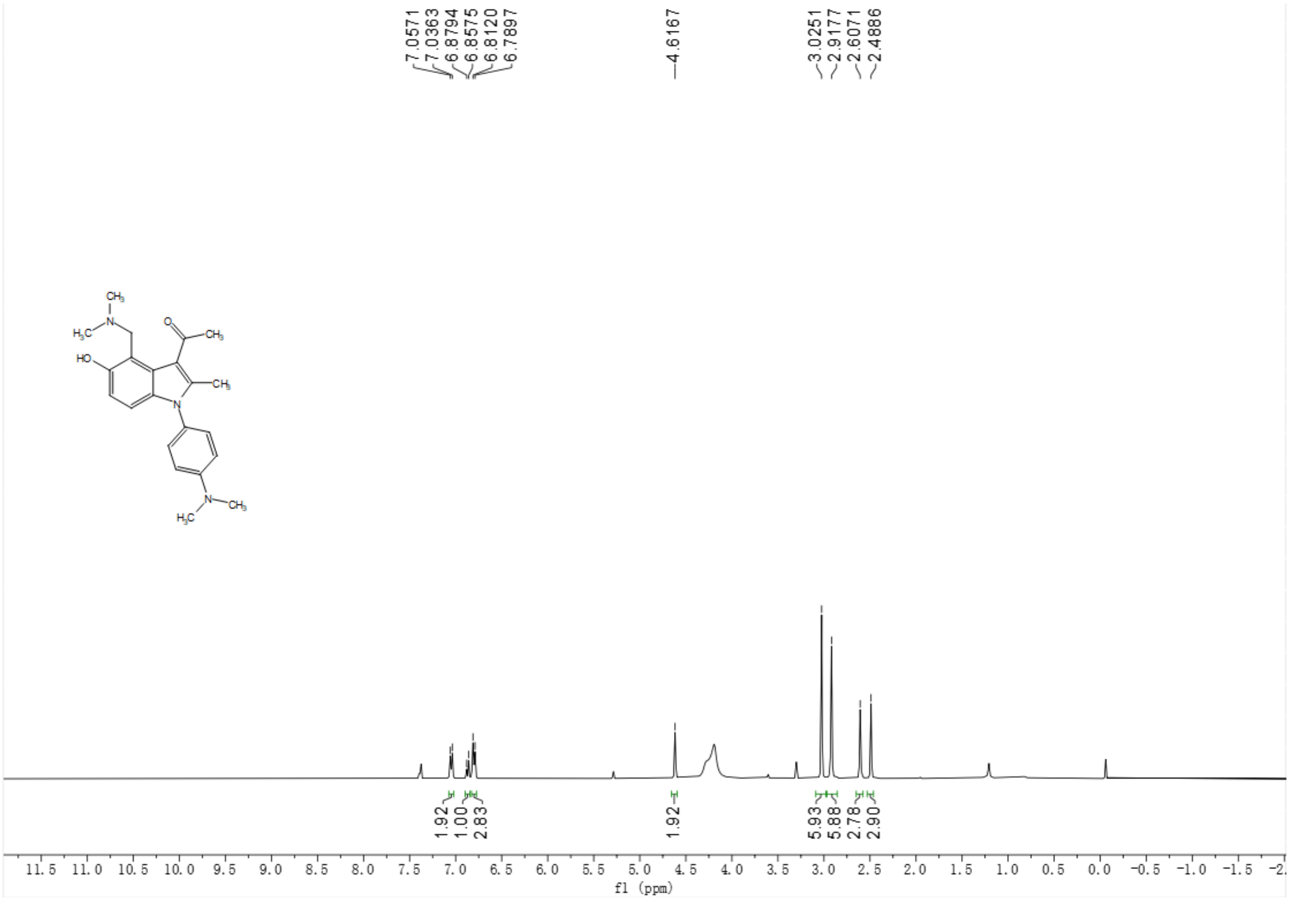
7.0571
7.0363
6.8794
6.8575
6.8120
6.7897
4.6167
3.0251
2.9177
2.6071
2.4886
1.92
1.00
2.83
1.92
5.93
5.88
2.78
2.90
f1 (ppm)

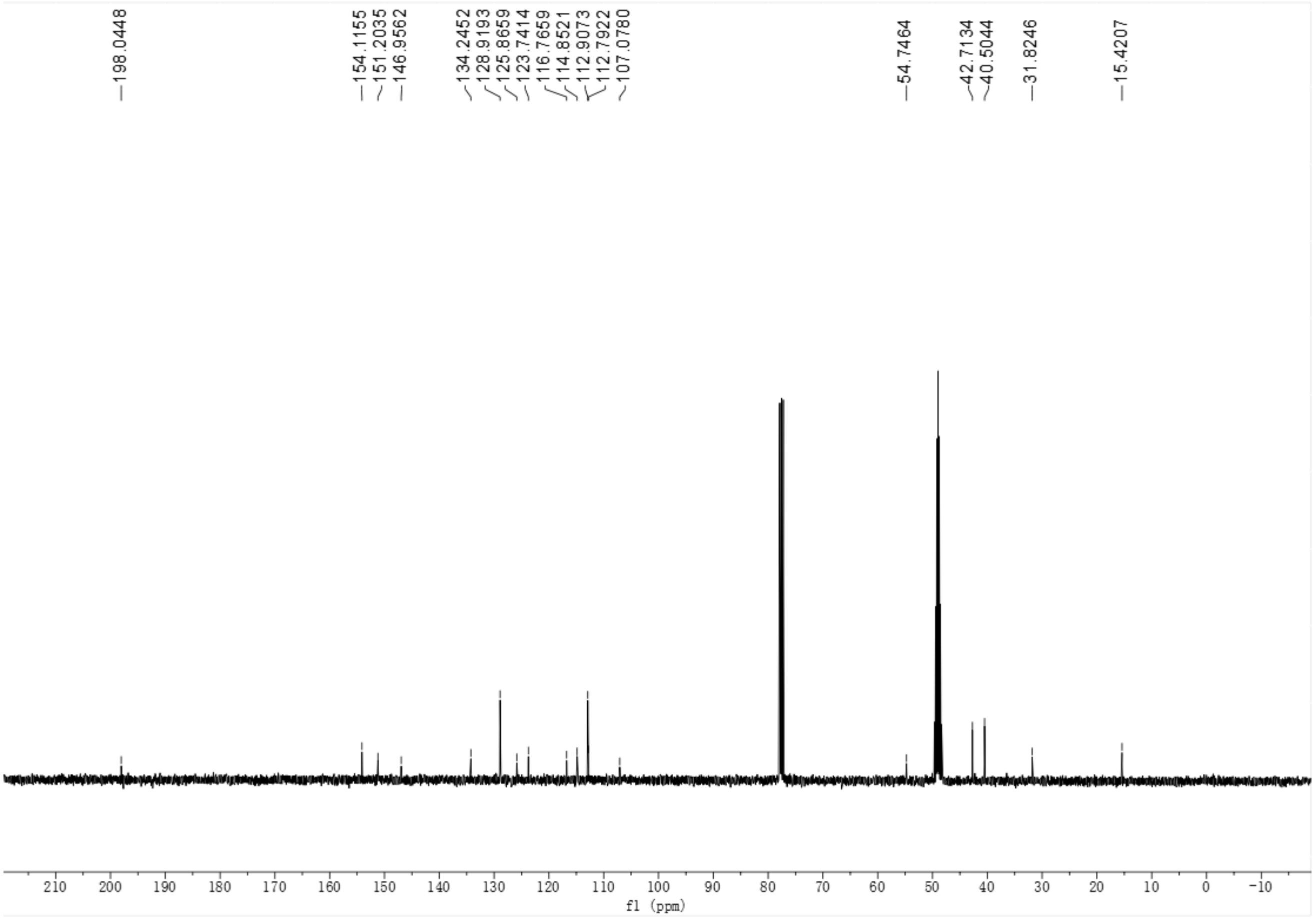
198.0448
154.1155
151.2035
146.9562
134.2452
128.9193
125.8659
123.7414
116.7659
114.8521
112.9073
112.7922
107.0780
54.7464
42.7134
40.5044
31.8246
15.4207
f1 (ppm)

52

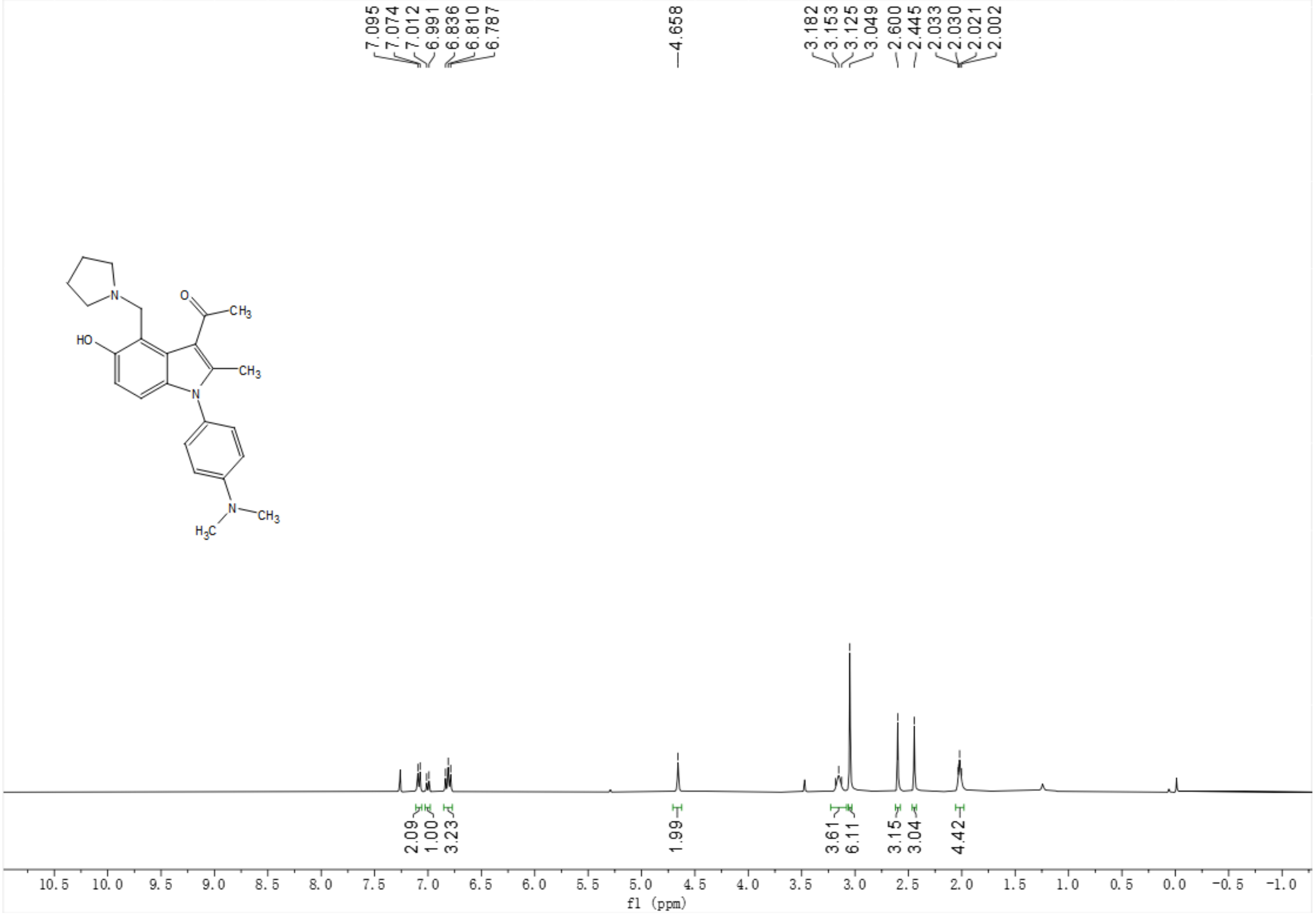
7.095
7.074
7.012
6.991
6.836
6.810
6.787
4.658
3.182
3.153
3.125
3.049
2.600
2.445
2.033
2.030
2.021
2.002
HO
O
CH3
CH3
N
N
H3C
CH3
2.09
1.00
3.23
1.99
3.61
6.11
3.15
3.04
4.42
10.5 10.0 9.5 9.0 8.5 8.0 7.5 7.0 6.5 6.0 5.5 5.0 4.5 4.0 3.5 3.0 2.5 2.0 1.5 1.0 0.5 0.0 -0.5 -1.0
f1 (ppm)

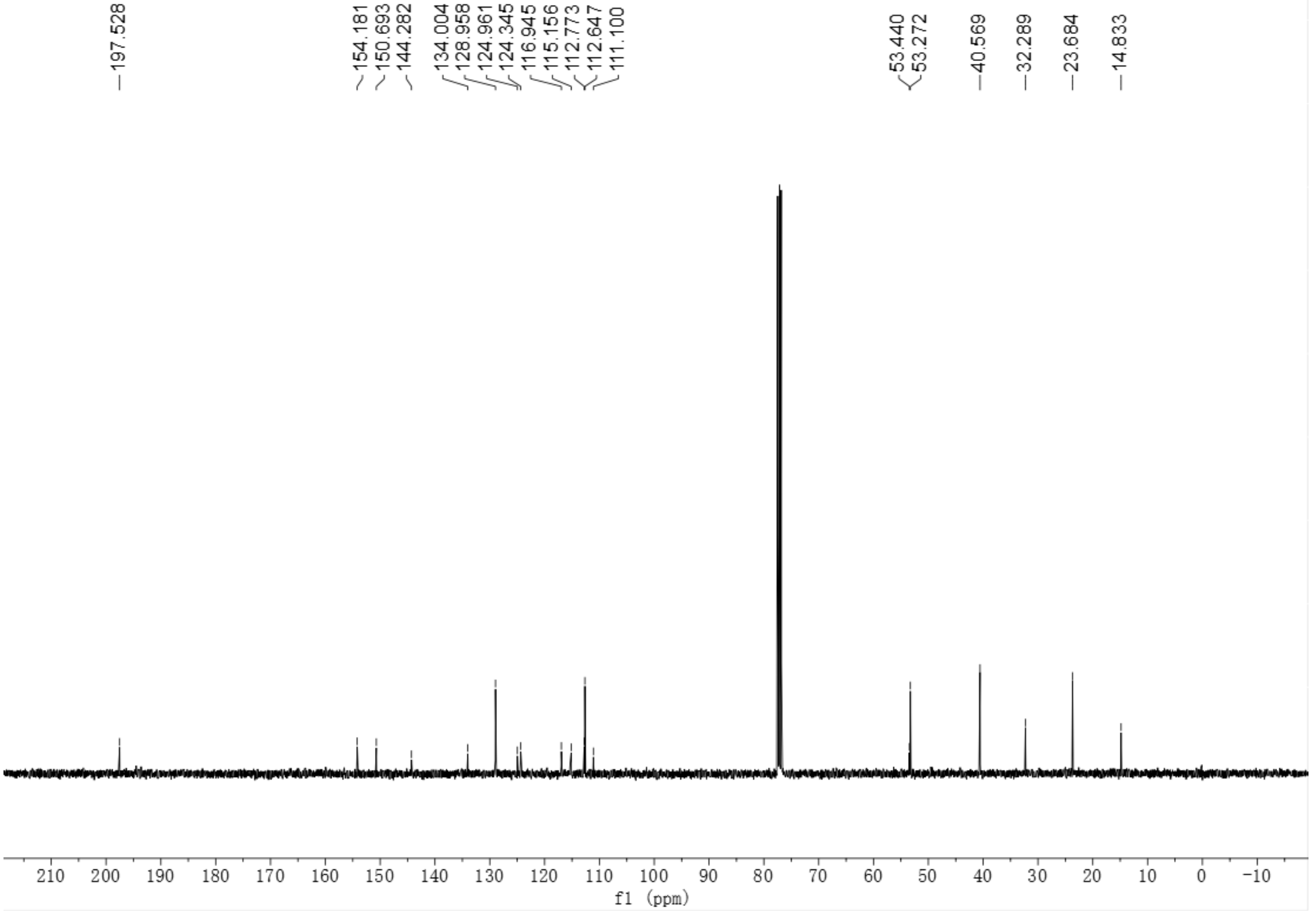
197.528
154.181
150.693
144.282
134.004
128.958
124.961
124.345
116.945
115.156
112.773
112.647
111.100
53.440
53.272
40.569
32.289
23.684
14.833
210 200 190 180 170 160 150 140 130 120 110 100 90 80 70 60 50 40 30 20 10 0 -10
f1 (ppm)

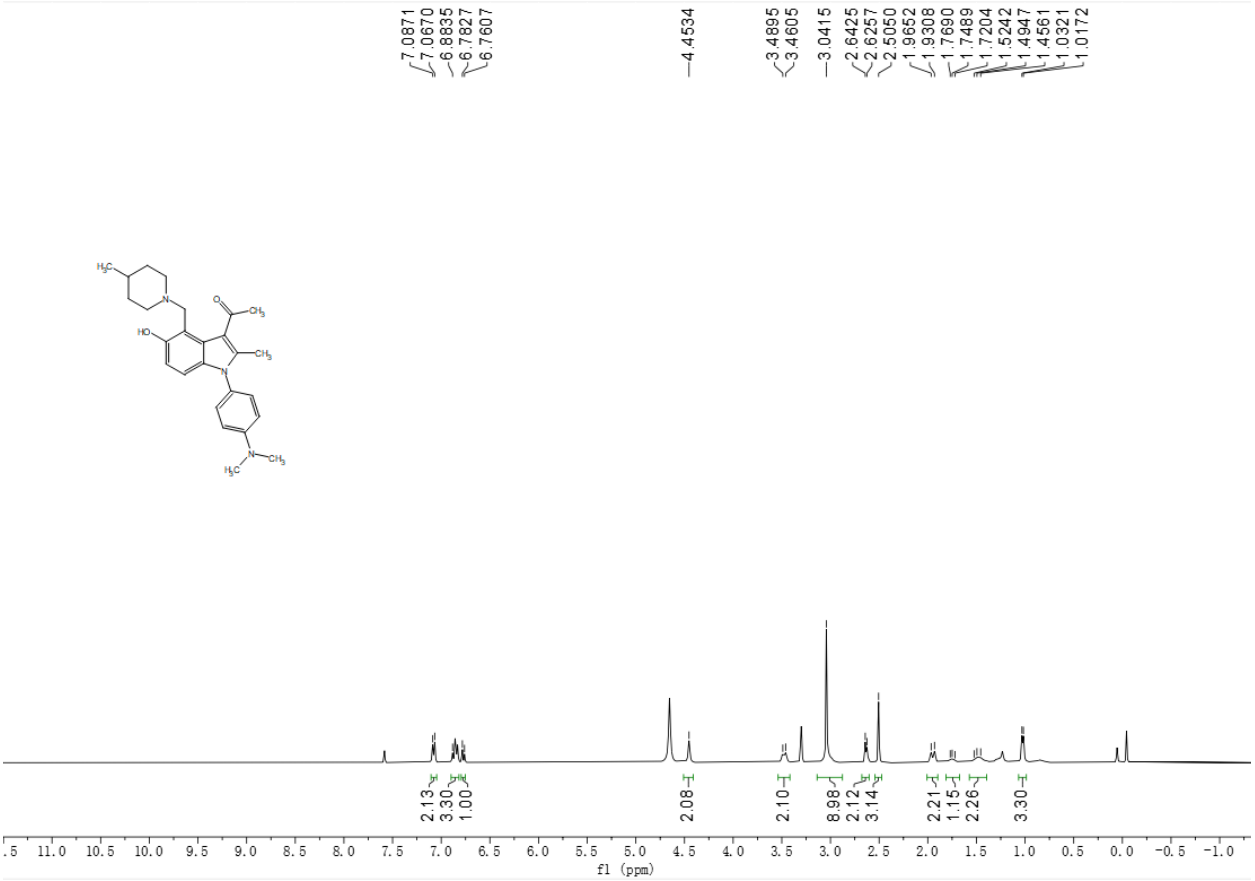
7.0871
7.0670
6.8835
6.7827
6.7607
4.4534
3.4895
3.4605
3.0415
2.6425
2.6257
2.5050
1.9652
1.9308
1.7690
1.7489
1.7204
1.5242
1.4947
1.4561
1.0321
1.0172
2.13
3.30
1.00
2.08
2.10
8.98
2.12
3.14
2.21
1.15
2.26
3.30
fl (ppm)

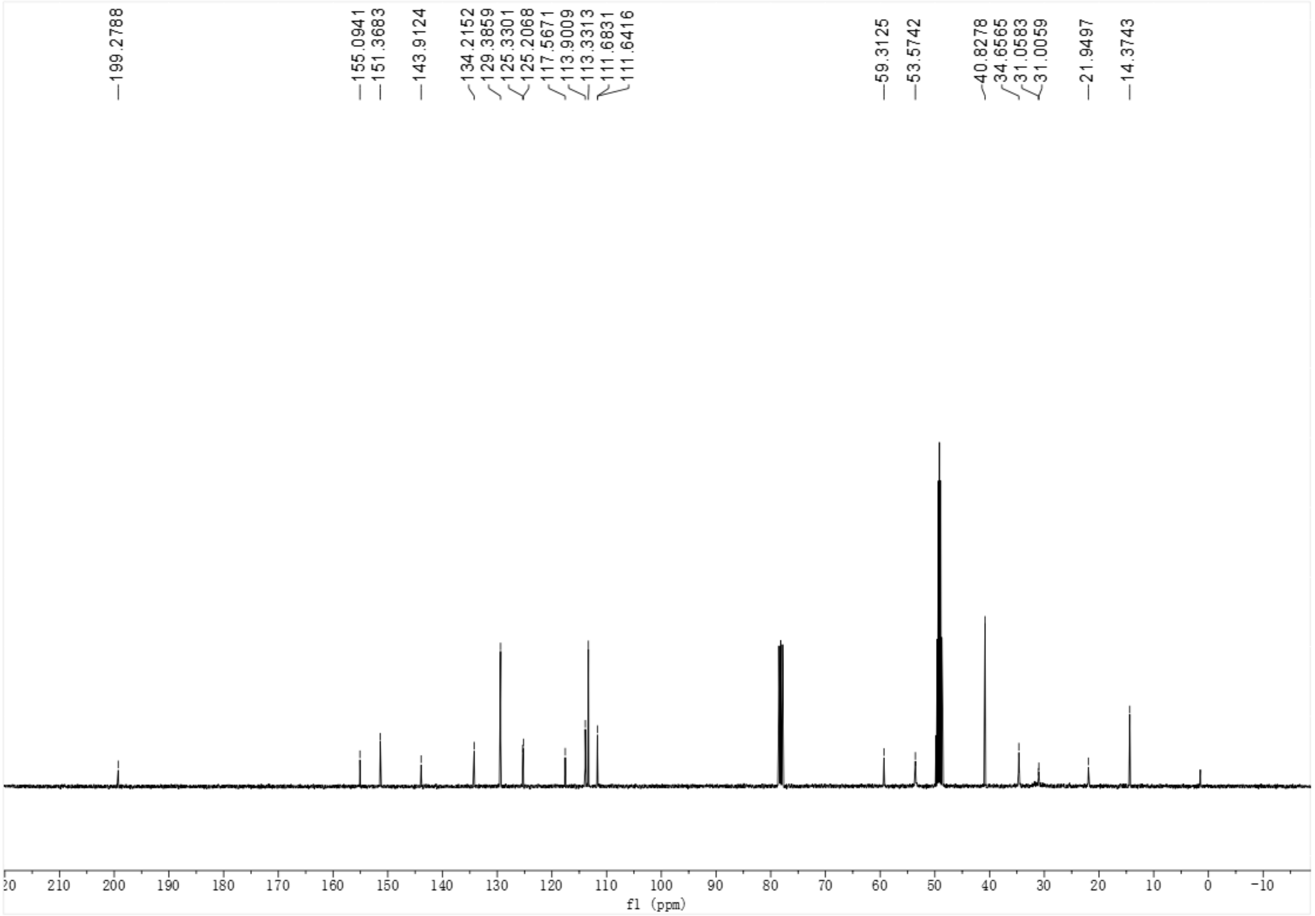
199.2788
155.0941
151.3683
143.9124
134.2152
129.3859
125.3301
125.2068
117.5671
113.9009
113.3313
111.6831
111.6416
59.3125
53.5742
40.8278
34.6565
31.0583
31.0059
21.9497
14.3743
fl (ppm)

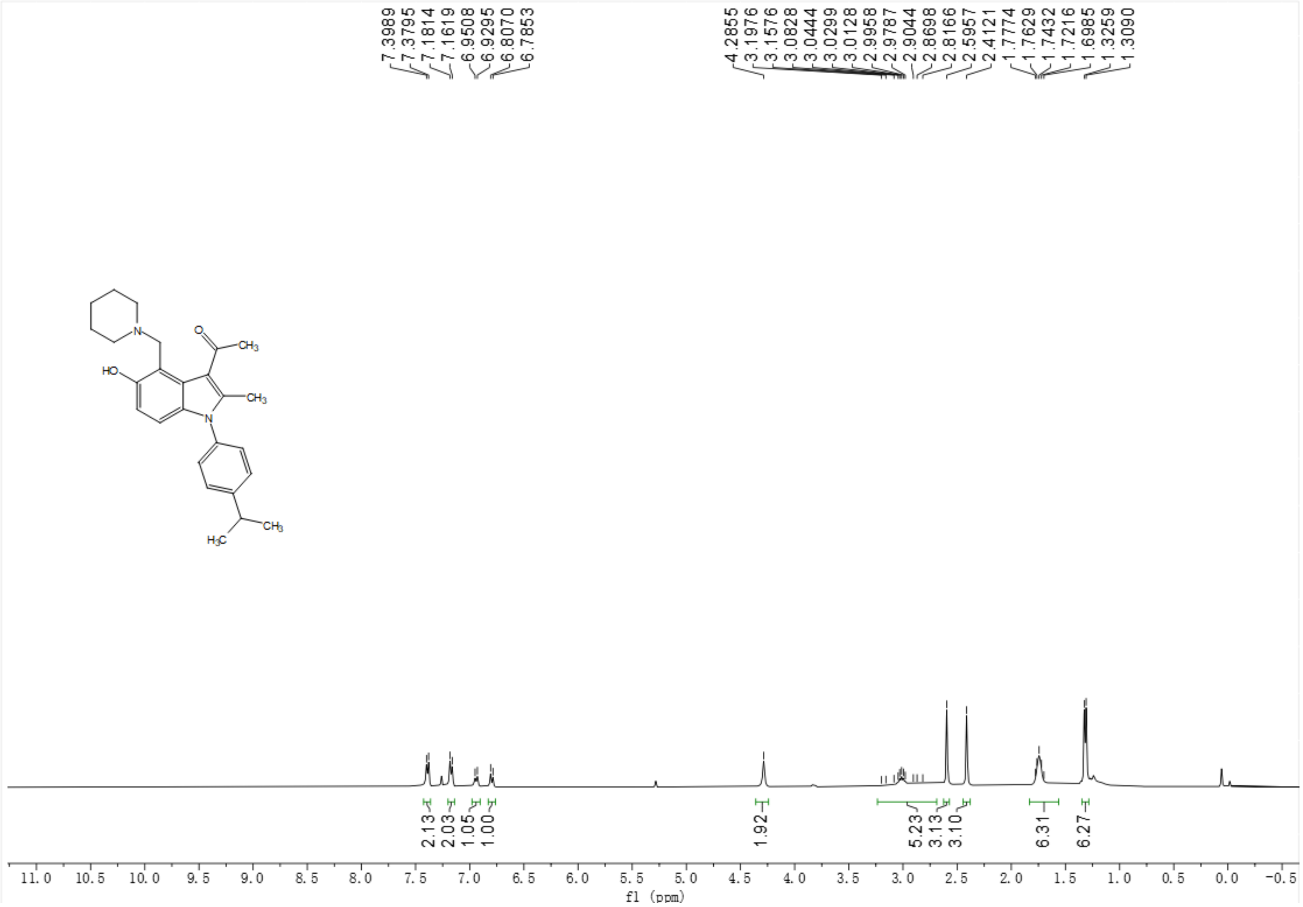
7.3989
7.3795
7.1814
7.1619
6.9508
6.9295
6.8070
6.7853
4.2855
3.1976
3.1576
3.0828
3.0444
3.0299
3.0128
2.9958
2.9787
2.9044
2.8698
2.8166
2.5957
2.4121
1.7774
1.7629
1.7432
1.7216
1.6985
1.3259
1.3090
HO
N
O
CH3
CH3
H3C
2.13
2.03
1.05
1.00
1.92
5.23
3.13
3.10
6.31
6.27
fl (ppm)

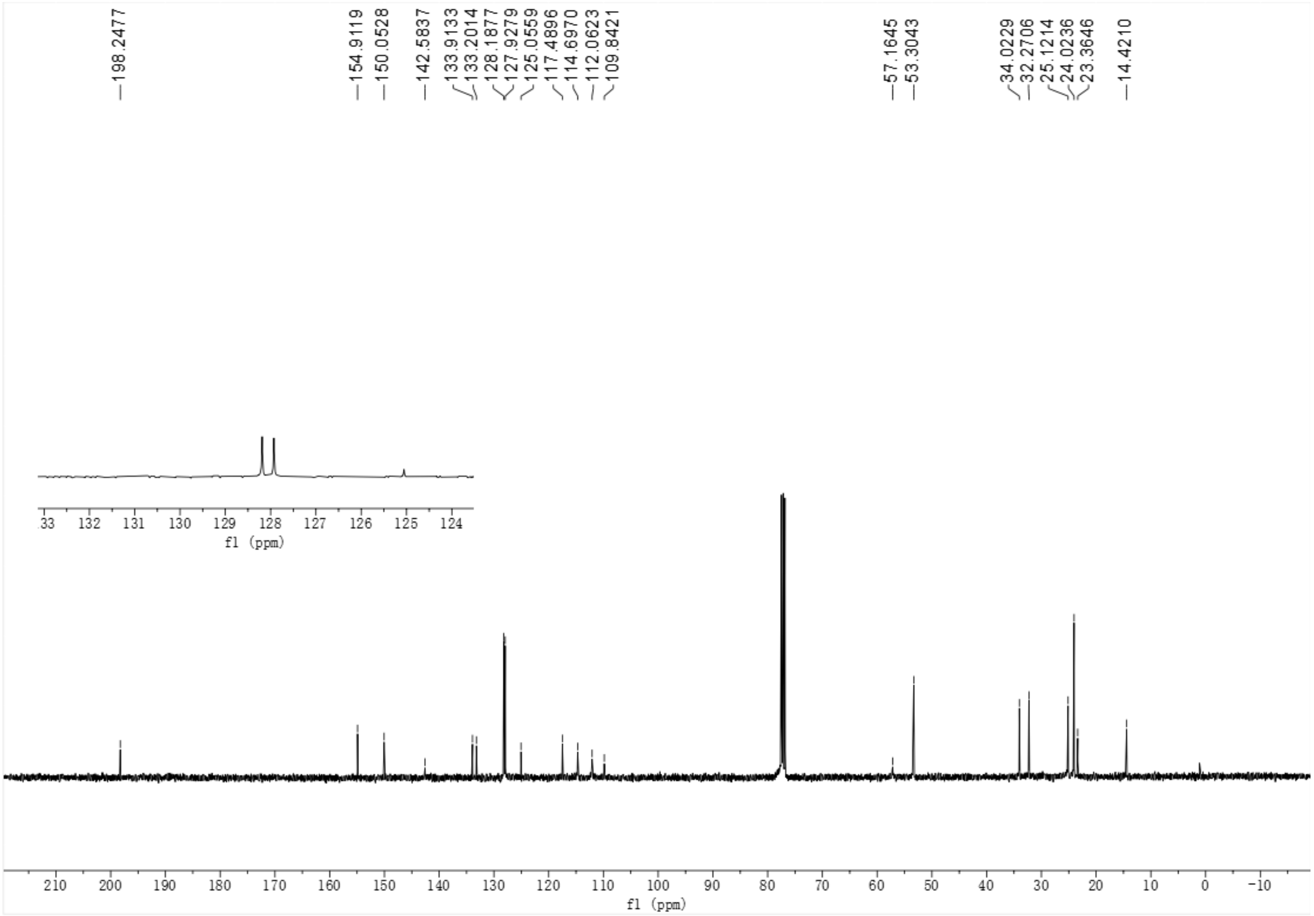
198.2477
154.9119
150.0528
142.5837
133.9133
133.2014
128.1877
127.9279
125.0559
117.4896
114.6970
112.0623
109.8421
57.1645
53.3043
34.0229
32.2706
25.1214
24.0236
23.3646
14.4210
fl (ppm)

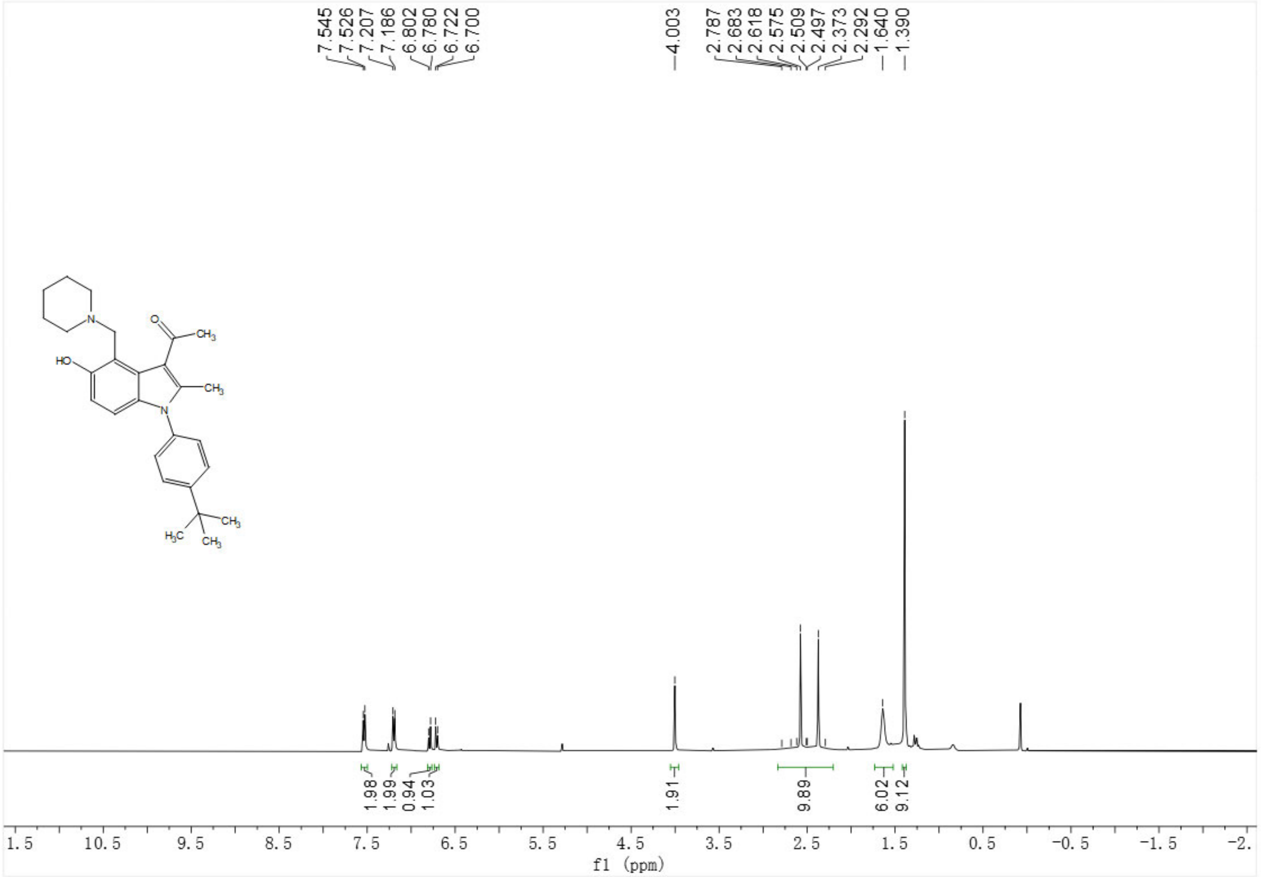
7.545
7.526
7.207
7.186
6.802
6.780
6.722
6.700
4.003
2.787
2.683
2.618
2.575
2.509
2.497
2.373
2.292
1.640
1.390
HO
N
O
CH3
CH3
H3C
CH3
CH3
1.98
1.99
0.94
1.03
1.91
9.89
6.02
9.12
1.5
10.5
9.5
8.5
7.5
6.5
5.5
4.5
3.5
2.5
1.5
0.5
-0.5
-1.5
-2.
f1 (ppm)

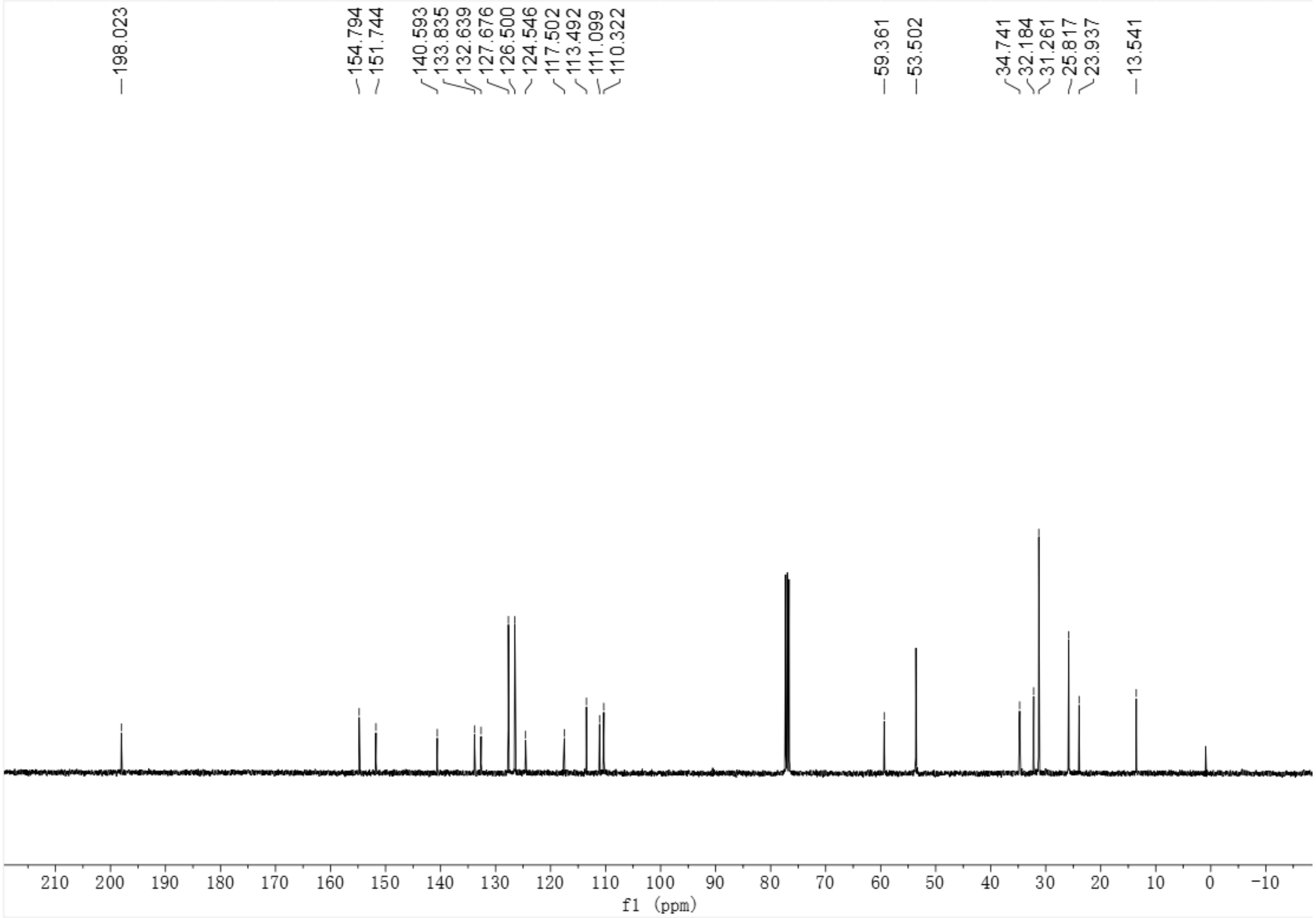
198.023
154.794
151.744
140.593
133.835
132.639
127.676
126.500
124.546
117.502
113.492
111.099
110.322
59.361
53.502
34.741
32.184
31.261
25.817
23.937
13.541
210
200
190
180
170
160
150
140
130
120
110
100
90
80
70
60
50
40
30
20
10
0
-10
f1 (ppm)

56

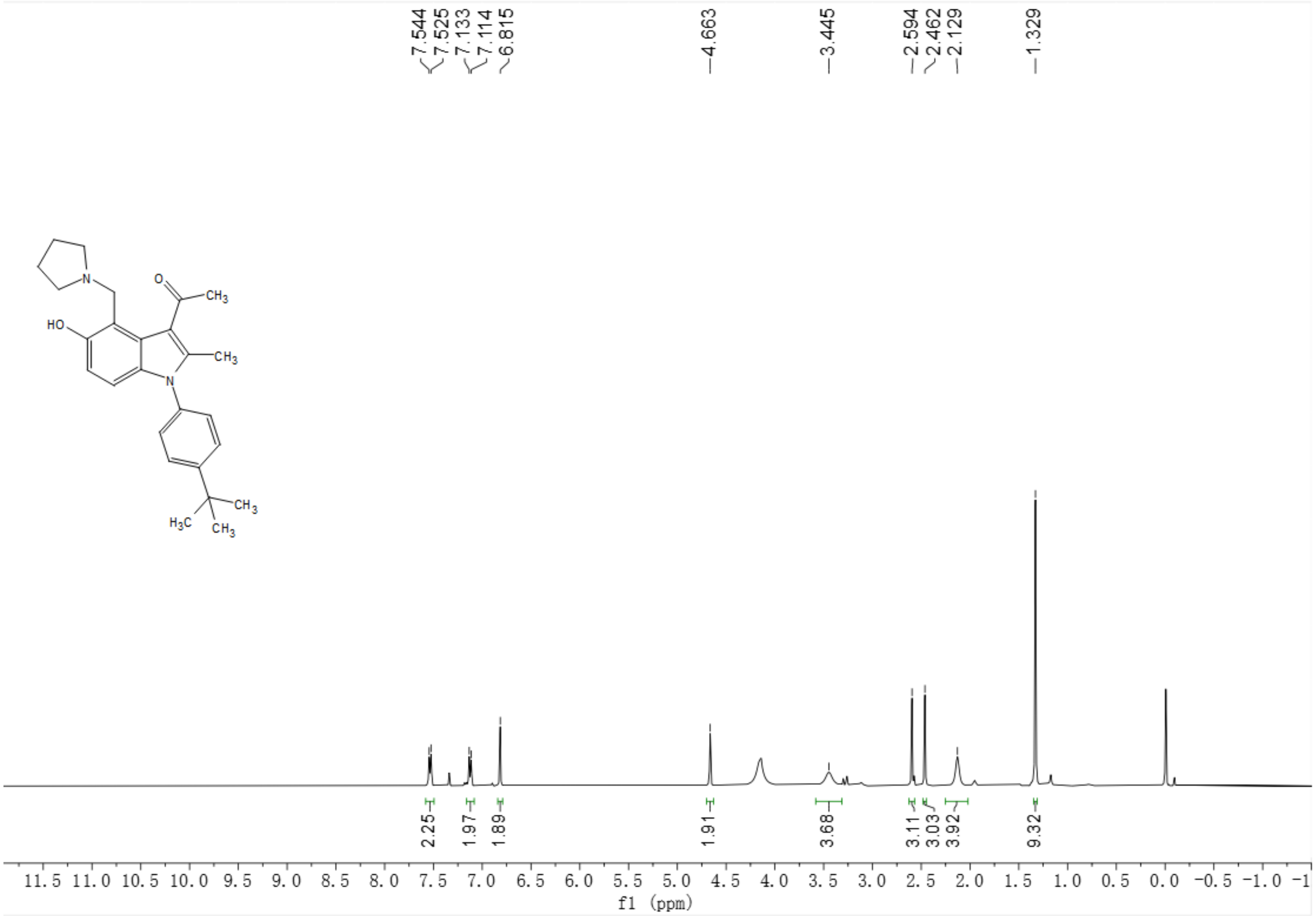

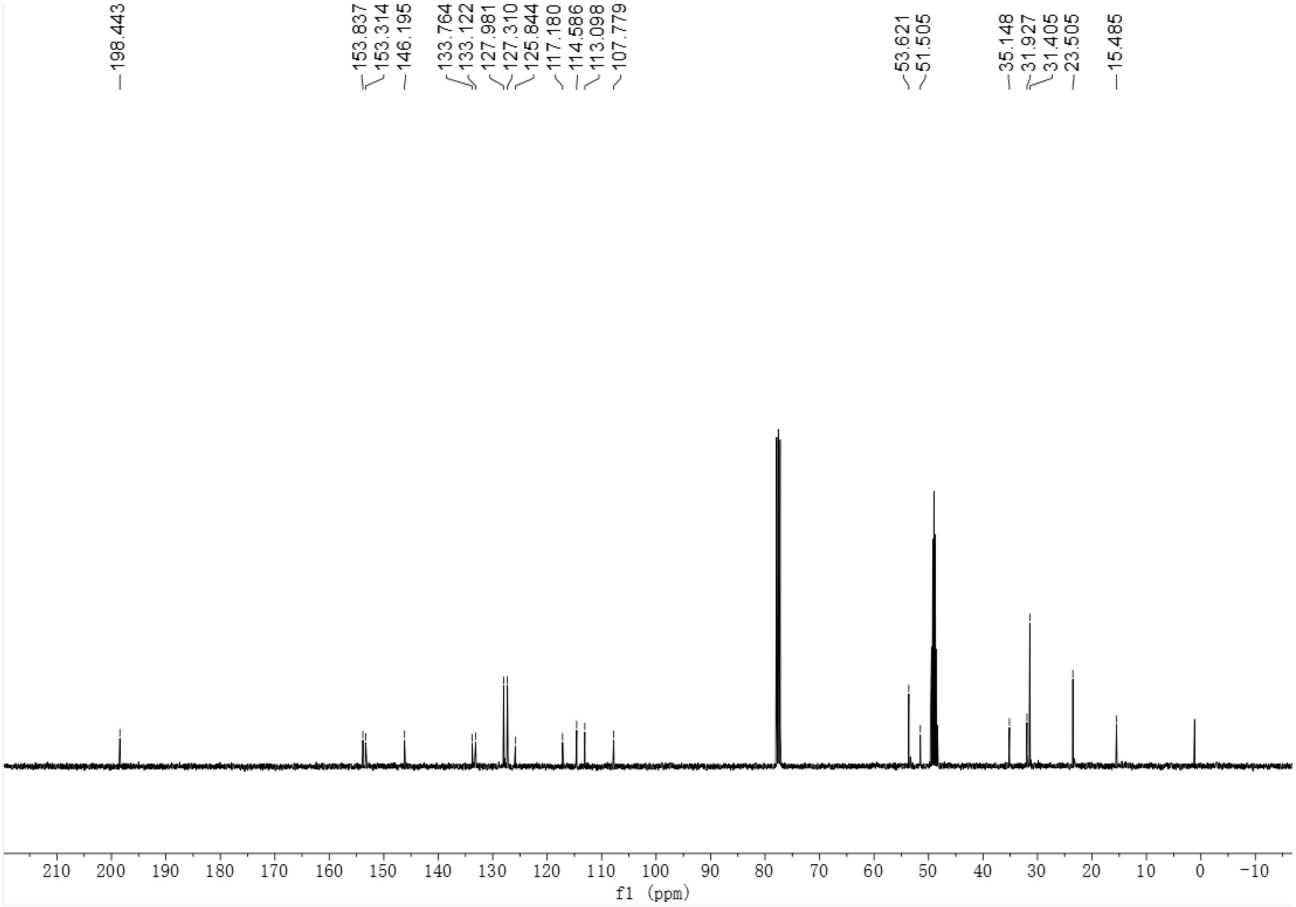

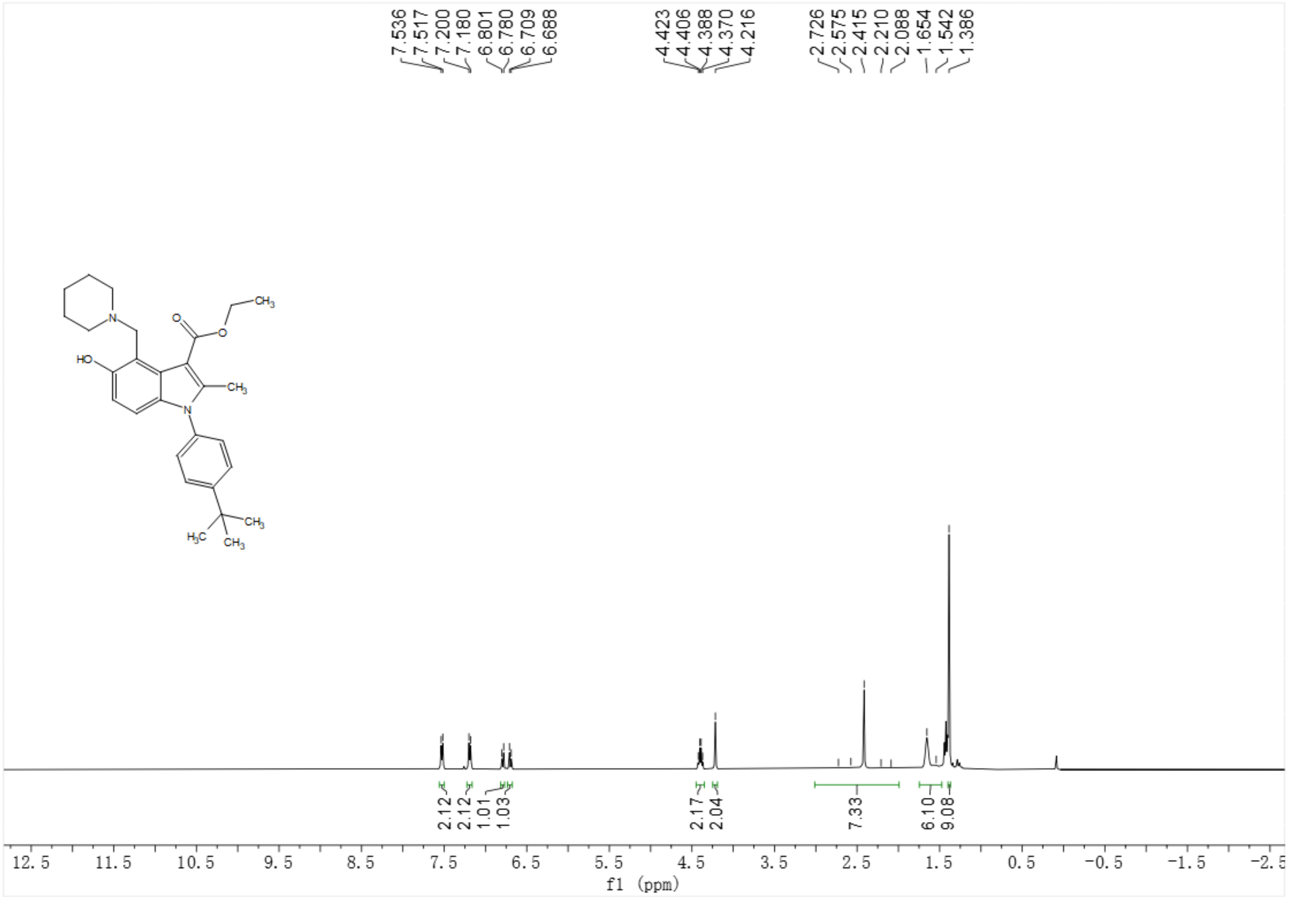
7.536
7.517
7.200
7.180
6.801
6.780
6.709
6.688
4.423
4.406
4.388
4.370
4.216
2.726
2.575
2.415
2.210
2.088
1.654
1.542
1.386
2.12
2.12
1.01
1.03
2.17
2.04
7.33
6.10
9.08
HO
N
O
O
CH3
CH3
H3C
f1 (ppm)

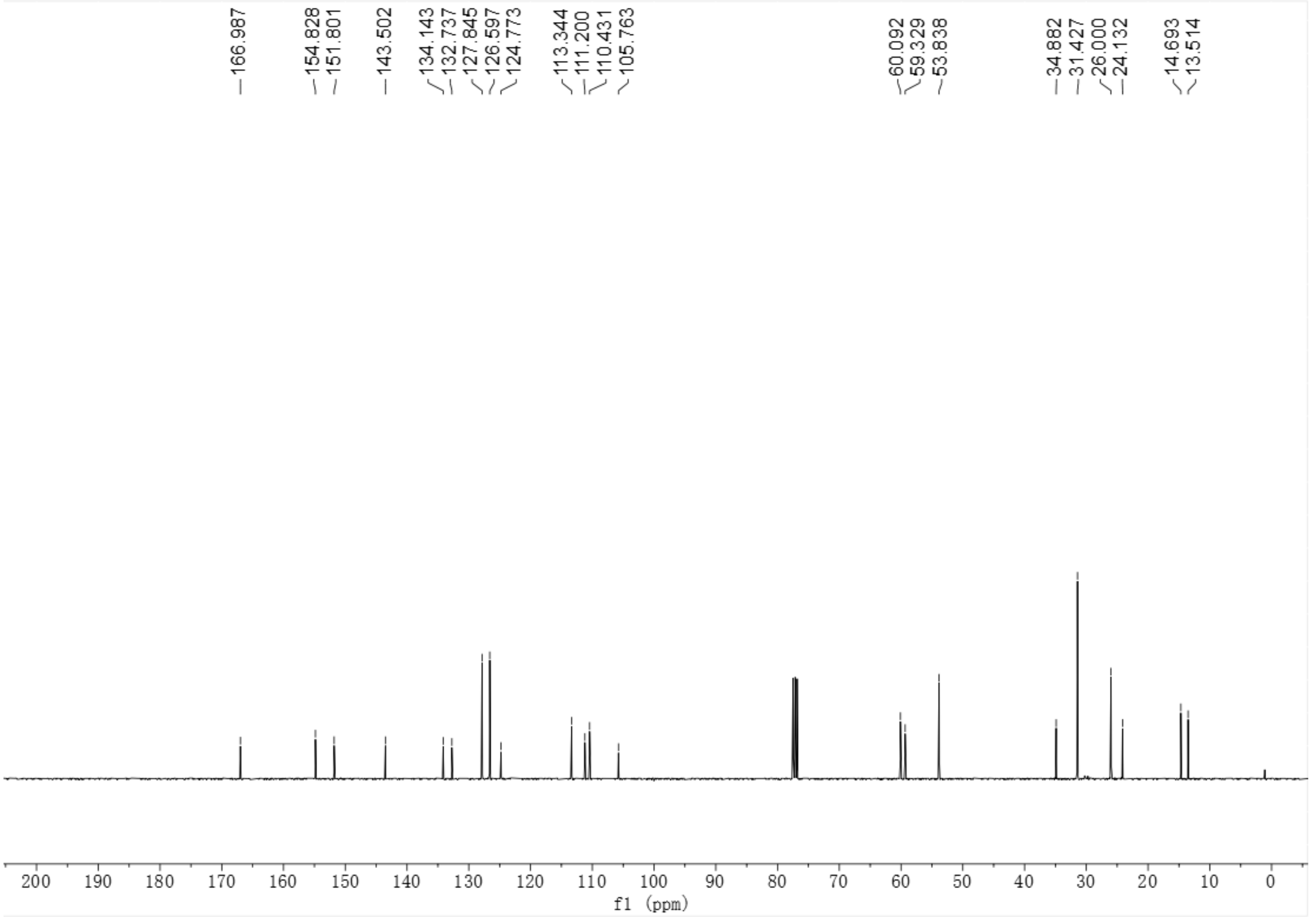
166.987
154.828
151.801
143.502
134.143
132.737
127.845
126.597
124.773
113.344
111.200
110.431
105.763
60.092
59.329
53.838
34.882
31.427
26.000
24.132
14.693
13.514
f1 (ppm)

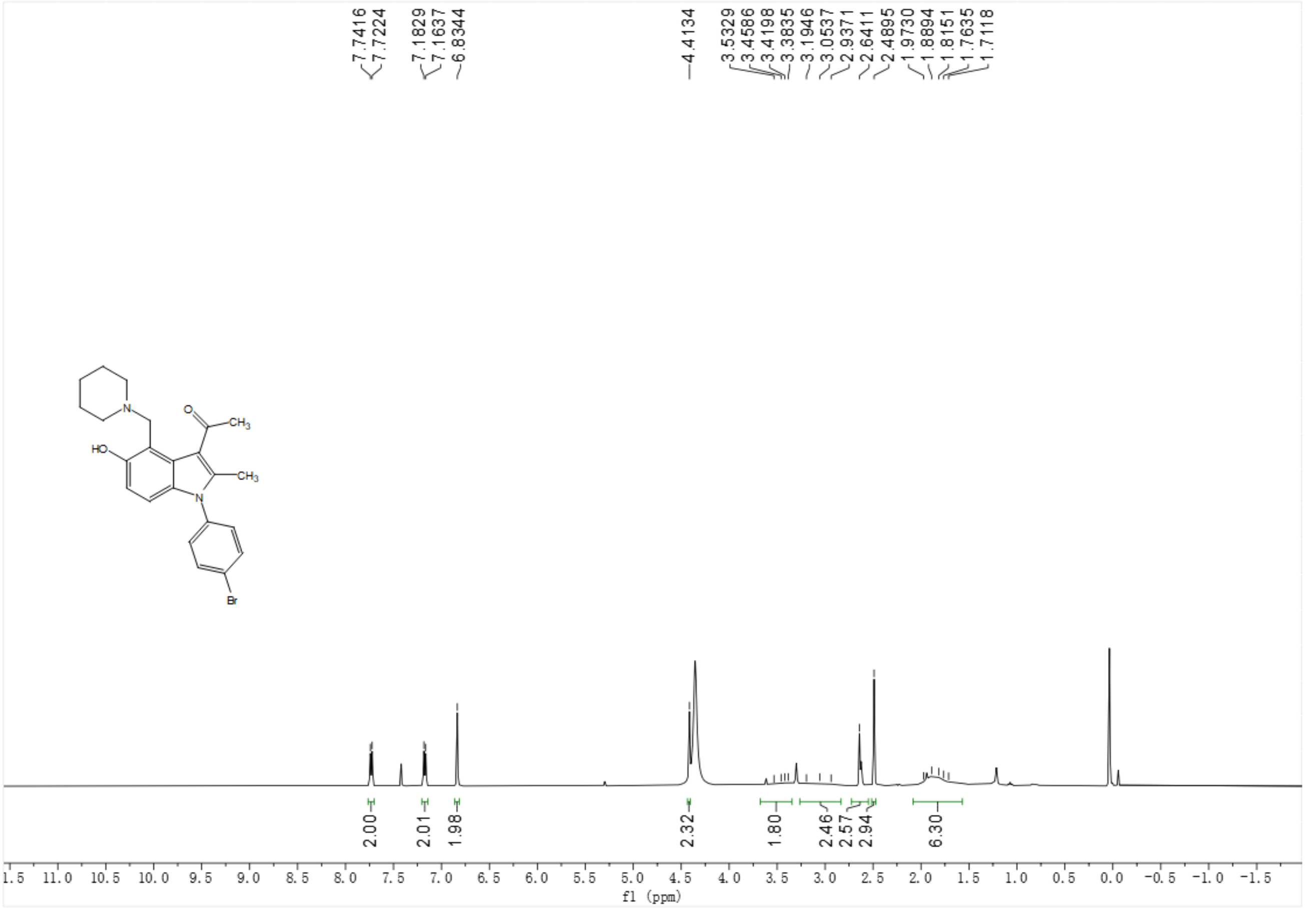

7.7416
7.7224
7.1829
7.1637
6.8344
4.4134
3.5329
3.4586
3.4198
3.3835
3.1946
3.0537
2.9371
2.6411
2.4895
1.9730
1.8894
1.8151
1.7635
1.7118
O
CH3
N
HO
CH3
N
Br
2.00
2.01
1.98
2.32
1.80
2.46
2.57
2.94
6.30
f1 (ppm)

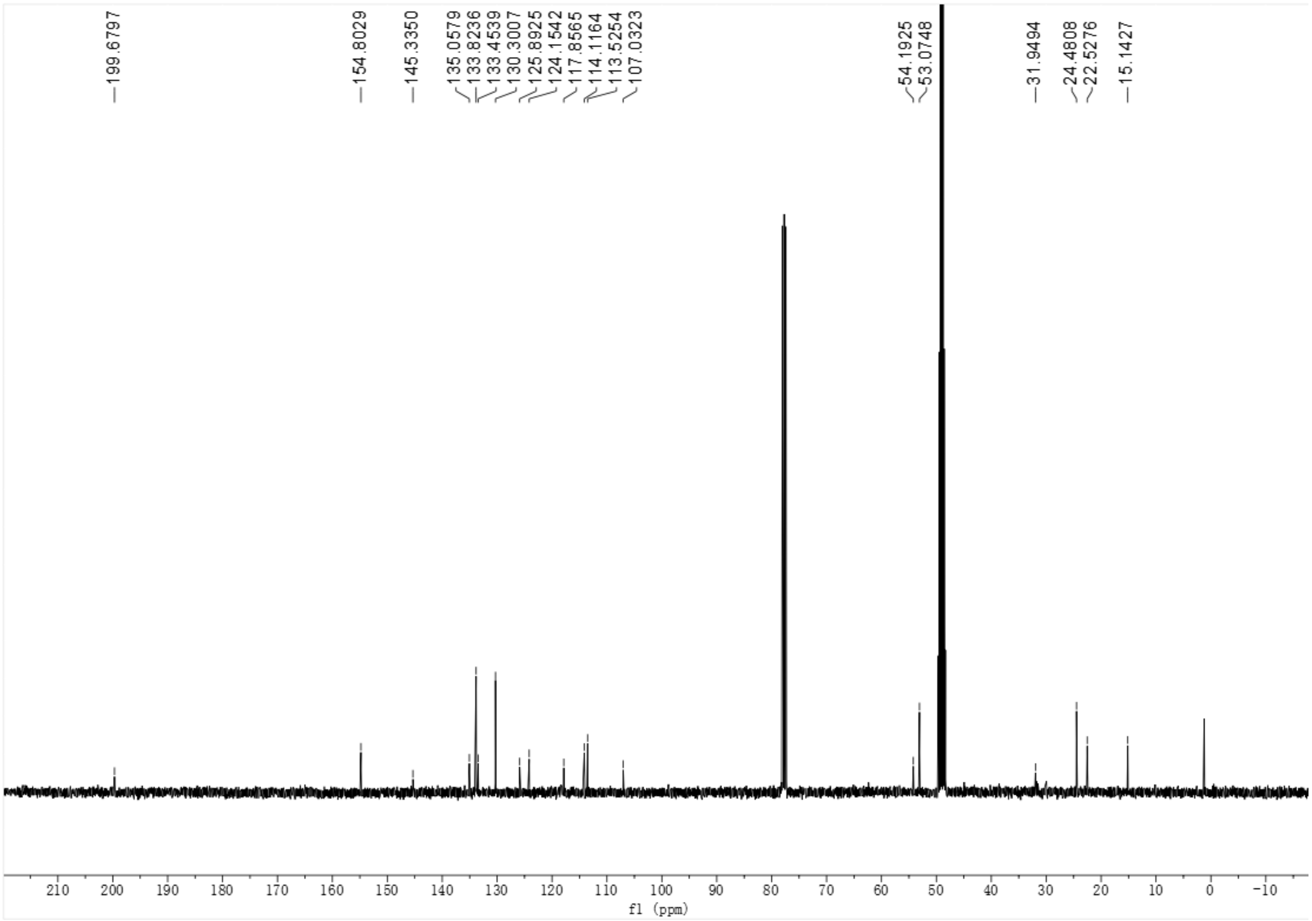

199.6797
154.8029
145.3350
135.0579
133.8236
133.4539
130.3007
125.8925
124.1542
117.8565
114.1164
113.5254
107.0323
54.1925
53.0748
31.9494
24.4808
22.5276
15.1427
f1 (ppm)

Table S1. Analytical HPLC purities of all final compounds.

**Compd**	**HPLC**			**Compd**	**HPLC**		
	**t_R (min)**	**Purity (%)**			**t_R (min)**	**Purity (%)**	
		254(nm)	280(nm)			254(nm)	280(nm)
**13**	13.348	96.91	96.67	**34**	14.132	96.77	95.40
**14**	12.186	97.67	95.75	**35**	13.815	96.58	95.75
**15**	12.770	98.63	97.99	**36**	14.042	97.28	95.081
**16**	13.245	99.72	97.20	**37**	13.955	95.75	95.11
**17**	13.491	95.95	95.00	**38**	13.755	97.10	96.13
**18**	13.569	96.03	95.18	**39**	13.090	99.29	96.92
**19**	14.350	97.72	96.62	**45**	14.322	99.31	98.40
**20**	13.476	96.91	97.12	**48**	12.877	97.20	95.81
**22**	14.535	96.57	95.73	**49**	14.355	97.01	95.87
**23**	13.946	96.28	95.36	**50**	13.253	95.38	95.01
**24**	12.590	99.30	97.46	**51**	12.306	97.35	96.93
**25**	13.035	98.26	95.60	**52**	13.004	95.01	95.01
**28**	14.049	98.29	96.17	**53**	13.829	97.11	96.82
**29**	12.981	98.71	95.06	**54**	14.206	95.66	95.86
**30**	13.090	99.29	96.92	**55**	14.447	98.82	95.27
**31**	14.411	97.64	96.59	**56**	14.721	99.23	97.33
**32**	14.057	97.91	96.55	**57**	15.108	99.64	99.64
**33**	13.989	99.26	98.21	**58**	13.557	99.50	98.44

HPLC purity determination for the target compounds

Method. Flow rate = 1.0 mL/min; gradient elution over 18 minutes, from 10% ACN-H_2O to 95% ACN-H_2O with 0.5% HCOOH, 280 nm, 254 nm; InertSustain-C18 column (5 μm 4.6×250 mm).

HPLC Traces for **45** (UV=254 nm)

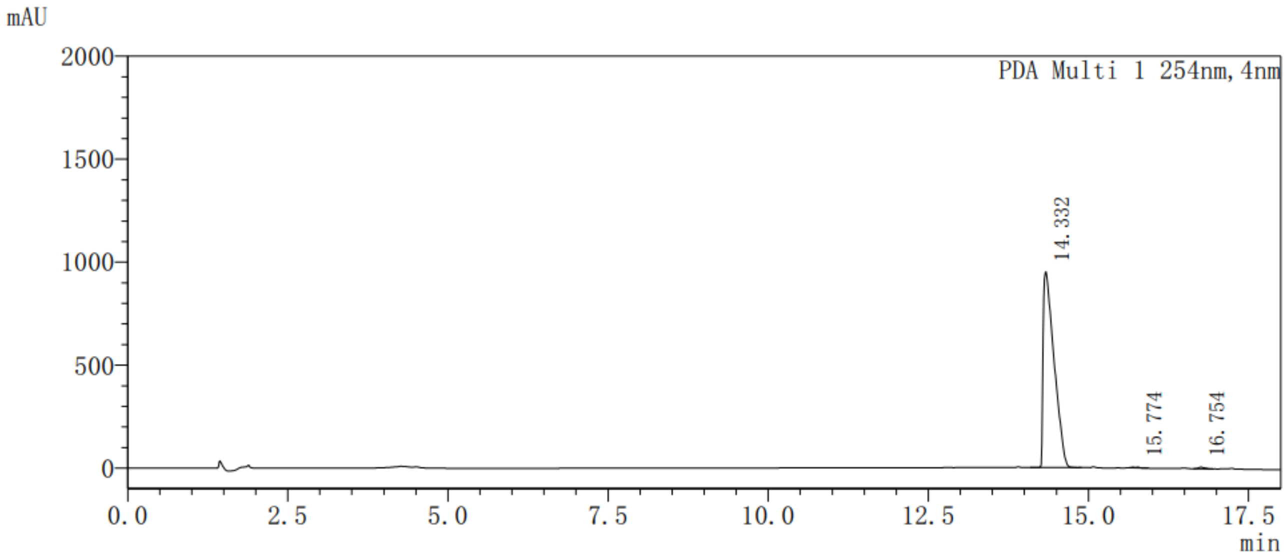

Peak Table

Peak	**Ret. time** (min)	**Area**	**Area** (%)
1	14.332	11279122	99.310
2	15.774	26585	0.234
3	16.754	51781	0.456

HPLC Traces for **45** (UV=280 nm).

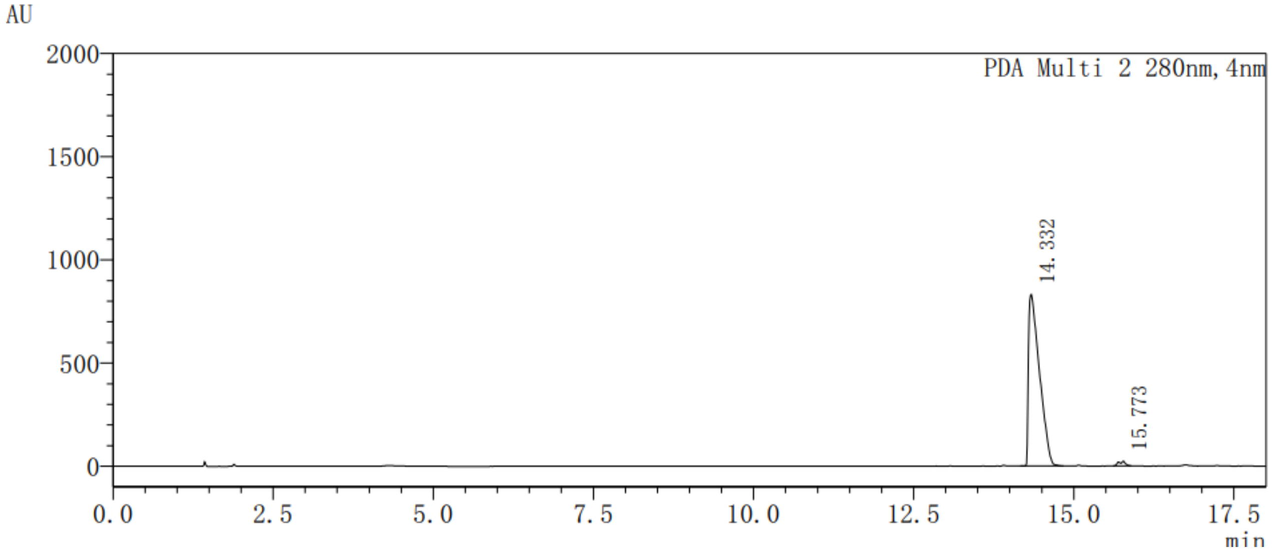

Peak Table

Peak	**Ret. time** (min)	**Area**	**Area** (%)
1	14.332	9946552	98.397
2	15.774	162088	1.603

Method. Flow rate = 1.0 mL/min; gradient elution over 18 minutes, from 10% ACN-H_2O to 95% ACN-H_2O with 0.5% HCOOH, 280 nm, 254 nm;

InertSustain-C18 column (5 μm 4.6×250 mm).

HPLC Traces for **58** (UV=254 nm)

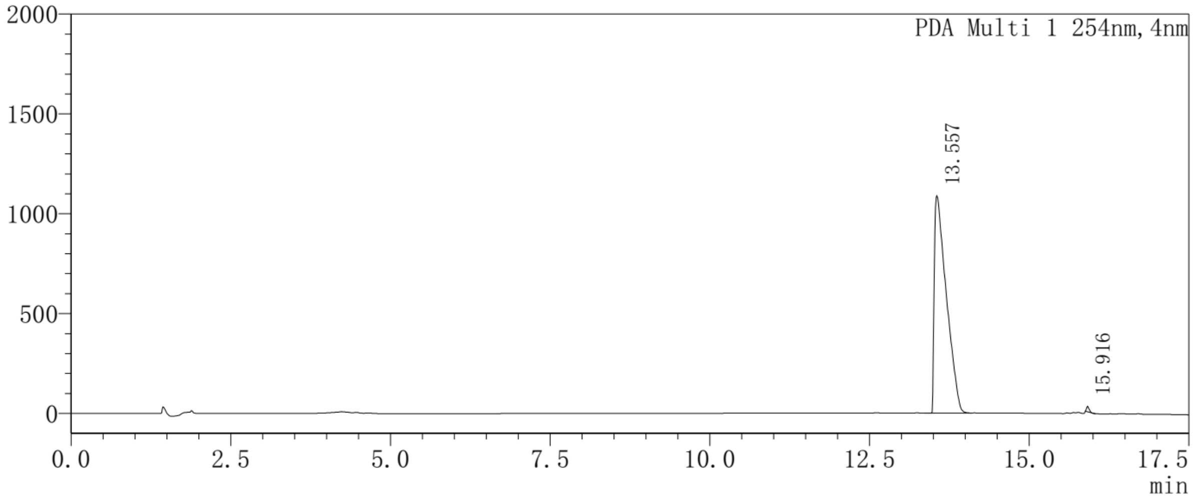

Peak Table

**Peak**	**Ret. time** (min)	**Area**	**Area** (%)
1	13.557	14408687	99.502
2	15.916	72043	0.498

HPLC Traces for **58** (UV=280 nm).

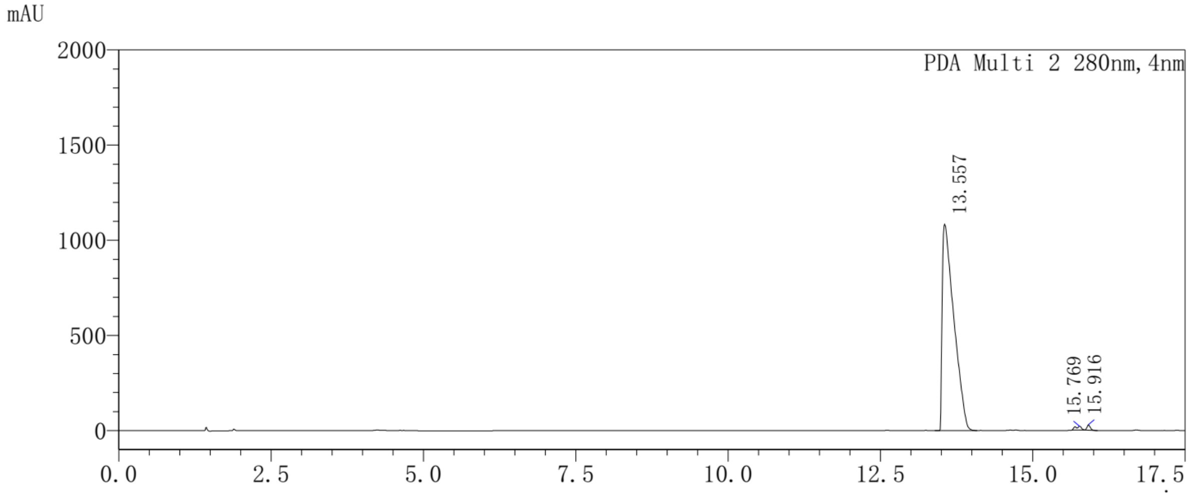

Peak Table

**Peak**	**Ret. time** (min)	**Area**	**Area** (%)
1	13.557	14464985	98.438
2	15.769	131683	0.896
3	15.916	97870	0.666

Method. Flow rate = 1.0 mL/min; gradient elution over 18 minutes, from 10% ACN-H_2O to 95% ACN-H_2O with 0.5% HCOOH, 280 nm, 254 nm;

InertSustain-C18 column (5 μm 4.6×250 mm).

HRMS for **45.**

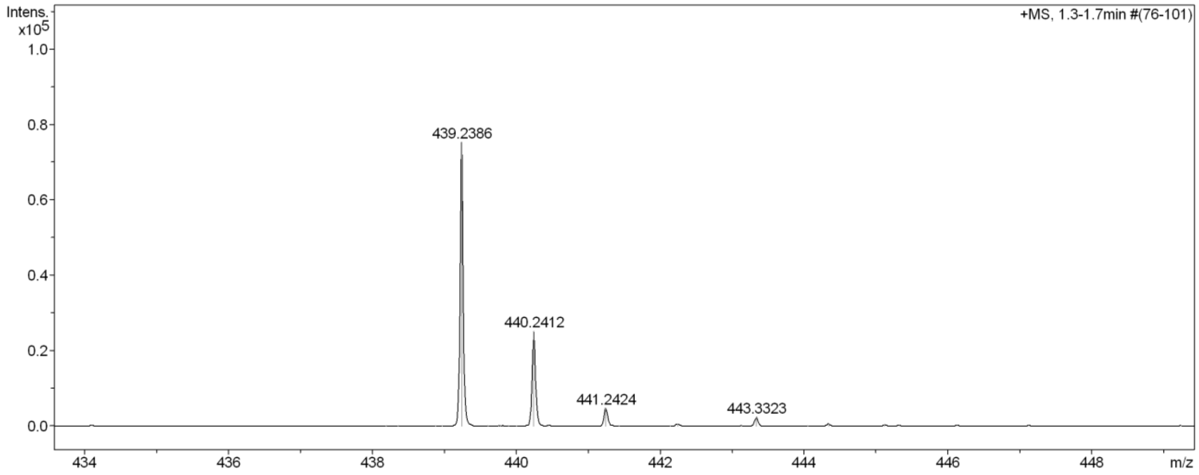

Meas. m/z	#	Formula	m/z	err [ppm]	Mean err [ppm]	rdb	N-Rule	e⁻ Conf	mSigma	Std I	Std Mean m/z	Std I VarNorm	Std m/z Diff	Std Comb Dev
439.2386														
	1	C 29 H 31 N 2 O 2	439.2380	-1.3	-0.5	15.5	ok	even	6.53	0.0121	0.0008	0.0042	0.0017	0.4160

HRMS for **58.**

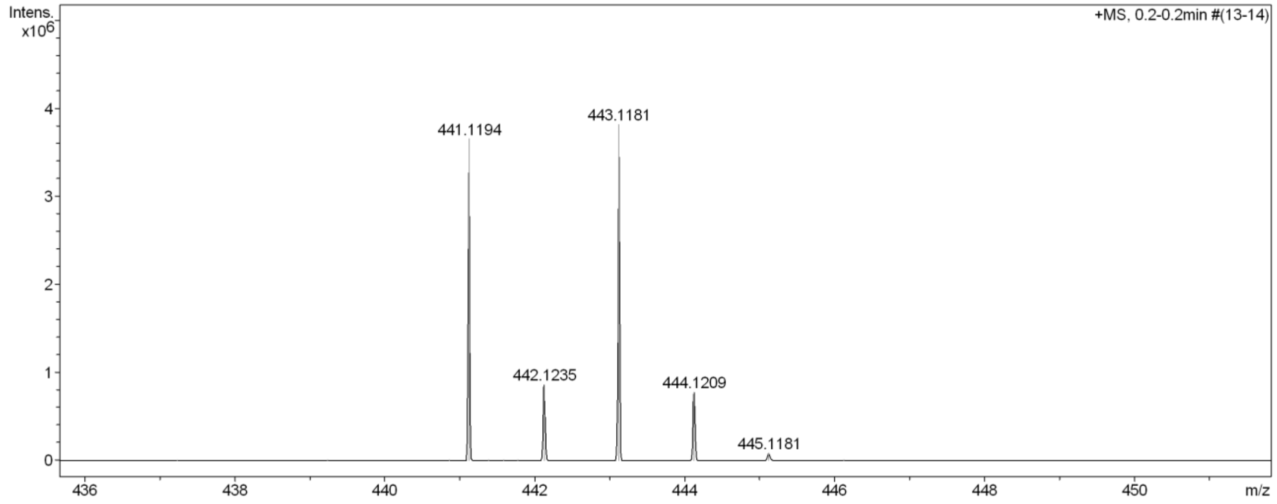

Meas. m/z	#	Formula	m/z	err [ppm]	Mean err [ppm]	rdb	N-Rule	e⁻ Conf	mSigma	Std I	Std Mean m/z	Std I VarNorm	Std m/z Diff	Std Comb Dev
441.1194														
	1	C 23 H 26 Br N 2 O 2	441.1172	-5.0	-5.6	11.5	ok	even	28.99	0.0262	0.0026	0.0095	0.0005	0.7810
